# Design, Synthesis, and Biological Evaluation of Novel Multitarget 7-Alcoxyamino-3-(1,2,3-triazole)-coumarins as Potent Acetylcholinesterase Inhibitors

**DOI:** 10.3390/ph18091398

**Published:** 2025-09-17

**Authors:** Nathalia F. Nadur, Larissa de A. P. Ferreira, Daiana P. Franco, Luciana L. de Azevedo, Lucas Caruso, Thiago da S. Honório, Priscila de S. Furtado, Alice Simon, Lucio M. Cabral, Tobias Werner, Holger Stark, Arthur E. Kümmerle

**Affiliations:** 1 Laboratory of Molecular Diversity and Medicinal Chemistry (LaDMol-QM), Graduate Program in Chemistry (PPGQ), Institute of Chemistry, Federal Rural University of Rio de Janeiro, Rio de Janeiro 23897-000, Brazil; nathaliafn18@gmail.com (N.F.N.); larissa.apf@live.com (L.d.A.P.F.); daiana_portella@yahoo.com.br (D.P.F.); luciana-la@hotmail.com (L.L.d.A.); lucascaruso4@gmail.com (L.C.); 2 Cell Culture Laboratory (LabCel), Department of Drugs and Pharmaceutics, Faculty of Pharmacy, Federal Rural University of Rio de Janeiro, Rio de Janeiro 21941-902, Brazil; honorio_thiago@yahoo.com.br (T.d.S.H.); prisouzafurtado@gmail.com (P.d.S.F.); alicesimon.pharma@yahoo.com (A.S.); lmcabral@pharma.ufrj.br (L.M.C.); 3 Institute of Pharmaceutical and Medicinal Chemistry, Heinrich Heine University Düsseldorf, 40225 Duesseldorf, Germany; t.werner@hhu.de (T.W.); stark@hhu.de (H.S.)

**Keywords:** Alzheimer disease, cholinesterase inhibitors, multitarget-directed ligands, click chemistry, H_3_R ligands

## Abstract

**Background**: Multitarget-directed ligands (MTDLs), particularly those combining cholinesterase inhibition with additional mechanisms, are promising candidates for Alzheimer’s disease (AD) therapy. Based on our previous identification of a dual-active coumarin derivative, we designed a new series of 7-alkoxyamino-3-(1,2,3-triazole)-coumarins. **Methods**: These compounds were synthesized by a new Sonogashira protocol and evaluated for AChE and BChE inhibition, enzymatic kinetics, molecular docking, neurotoxicity in SH-SY5Y cells, neuroprotection against H_2_O_2_-induced oxidative stress, and additional interactions with H_3_R and MAOs. **Results**: All derivatives inhibited AChE with IC_50_ values of 4–104 nM, displaying high selectivity over BChE (up to 686-fold). Kinetic and docking studies indicated mixed-type inhibition involving both CAS and PAS. The most potent compounds (**1h**, **1j**, **1k**, **1q**) were non-neurotoxic up to 50 µM, while **1h** and **1k** also showed neuroprotective effects at 12.5 µM. Selected derivatives (**1b**, **1h**, **1q**) demonstrated multitarget potential, including H_3_R affinity (K_i_ as low as 32 nM for **1b**) and MAO inhibition (IC_50_ of 1688 nM for **1q**). **Conclusions**: This series of coumarin–triazole derivatives combines potent and selective AChE inhibition with neuroprotective and multitarget activities, highlighting their promise as candidates for AD therapy.

## 1. Introduction

Alzheimer’s disease (AD) is a progressive neurodegenerative disorder characterized by neuronal degeneration, gradual memory loss, cognitive impairment, and behavioral disturbances [1,2]. Although its precise etiology remains elusive, AD is widely regarded as a multifactorial condition arising from the interplay of multiple pathogenic pathways, including cholinergic dysfunction, β-amyloid (Aβ) plaque accumulation, tau protein hyperphosphorylation, oxidative stress, histaminergic system dysregulation, and chronic neuroinflammation [1,3,4,5,6].

Acetylcholinesterase (AChE) plays a crucial role in the degradation of acetylcholine (ACh), and under pathological conditions, excessive AChE activity can lead to a deficiency of this critical neurotransmitter. While conventional AChE inhibitors fail to halt the progression of AD, multitarget-directed ligands (MTDLs) can simultaneously reduce Aβ plaque formation, and fight neuroinflammation, among other effects, positively impacting disease progression [6,7,8,9].

The active site of AChE consists of two primary regions: the catalytic active site (CAS) and the peripheral site (PAS). The CAS is located at the bottom of the cavity and contains a catalytic triad (Ser203, Glu334, and His447) [10,11], while the PAS is positioned at the cavity entrance that conducts the ACh to the CAS.

Coumarins represent a privileged scaffold for the design of multifunctional agents against AD due to their ability to interact with both CAS and PAS of AChE, as well as displaying a wide range of AD target modulations [12,13,14]. Likewise, 1,2,3-triazoles exhibit versatile pharmacological properties relevant to AD, including cholinesterase inhibition, suppression of Aβ aggregation, and neuroprotection [15,16]. The integration of these two moieties forms a robust foundation for MTDL design, enabling modulation of AChE alongside additional targets such as β-amyloid aggregation, oxidative stress, the histamine H_3_ receptor (H_3_R), and monoamine oxidases (MAOs) [17,18,19,20].

In this study, we pursued a dual-site inhibition strategy targeting both the CAS and PAS of AChE, as performed by the reference drug donepezil [21]. Our first objectives were to design, synthesize, and pharmacologically evaluate novel 7-alcoxyamino-3-(1,2,3-triazole)-coumarin derivatives (**1**) as selective inhibitors of AChE over butyrylcholinesterase (BChE).

**Figure 1:**
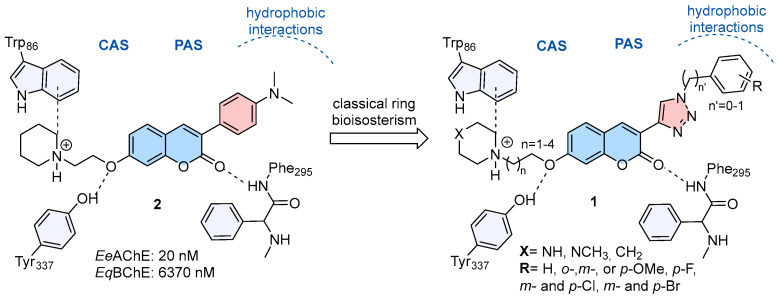
Structural design of 3-(1,2,3-triazole)-coumarin derivatives (**1**) as AChE inhibitors.

The compounds design was guided by a pharmacophore model based on a series of coumarins previously described by our research group (Figure 1) [22]. In this study, we aimed to retain the aminoalkoxy-coumarin scaffold of compound (**2**), known to interact with the CAS, while exploring various cyclic amines (X) and alkyl chain lengths (n) to optimize CAS binding. To improve interactions with PAS, a classical bioisosteric replacement of the phenyl ring in (**2**) with a 1,2,3-triazole group substituted with hydrophobic aromatic moieties was proposed, aiming at obtaining new interactions with the PAS.

Effects of conformational restrictions in these substituents were also planned with spacers of n’ = 0 or 1 for phenyl or benzyl substituents (R), respectively (Figure 1). Additionally, the best compounds were selected for further neurotoxicity and neuroprotection evaluations, as well as studies as MTDLs by modulating H_3_R and MAOs.

## 2. Results

### 2.1. Compounds Synthesis

**Scheme 1:**
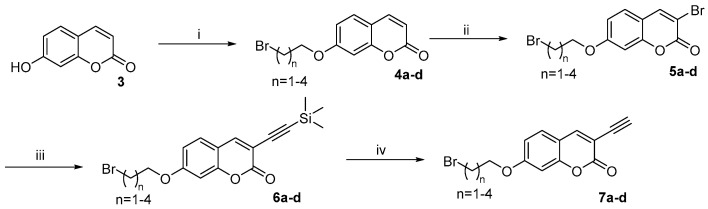
Synthesis of key acetylenes (**7a**–**d**) intermediates. Reagents and conditions: (i) Br(CH_2_)_n_Br (*n* = 2–5), K_2_CO_3_, acetone, 60 °C, 6–12 h, 46–71%; (ii) Br_2_, AcOH, NaOAc, r.t., 2 h, 83–93%; (iii) trimethylsilylacetylene, PdCl_2_(PPh_3_)_2_, CuI, Et_3_N, acetonitrile, 60 °C, 2 h, 54–66%; (iv) K_2_CO_3_, methanol, r.t., 1 h, 83–93%.

The synthetic pathway starts with the *O*-alkylation reaction of 7-hydroxycoumarin (**3**) with a series of dibromo alkanes with different chain lengths. Subsequently, the bromination of position 3 of the coumarin nucleus using Br_2_ in buffered medium of sodium acetate/acetic acid was performed via electrophilic substitution (ES) [22]. For the introduction of the acetylene group, the Sonogashira cross-coupling reaction was performed between the intermediate (**6a**–**d**) and trimethylsilyl acetylene, followed by deprotection of the acetylenes (**7a**–**d**) under basic conditions (Scheme 1).

**Scheme 2:**
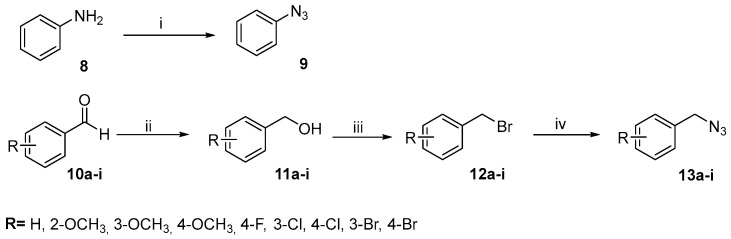
Synthesis of phenyl (**1**) and benzyl azides (**13a**–**i**). Reagents and conditions: (i) NaNO_2_, HCl, NaN_3_, 0 °C–r.t., 79%; (ii) NaBH_4_, ethanol, r.t., 3 h, 79–96% (iii) CBr_4_, PPh_3_, r.t. 2 h; (iv) NaN_3_, acetone/H_2_O, r.t., 30 min, 35–90%.

In parallel, phenyl azide (**9**) was obtained through the diazotization reaction between aniline (**8**) and sodium azide. The benzyl azides (**13a**–**i**) were prepared from benzaldehydes (**10a**–**i**), which were first reduced to their corresponding alcohols (**11a**–**i**) followed by a bromination reaction and finally to the formation of benzyl azides via nucleophilic substitution (S_N_2) (Scheme 2).

**Scheme 3:**
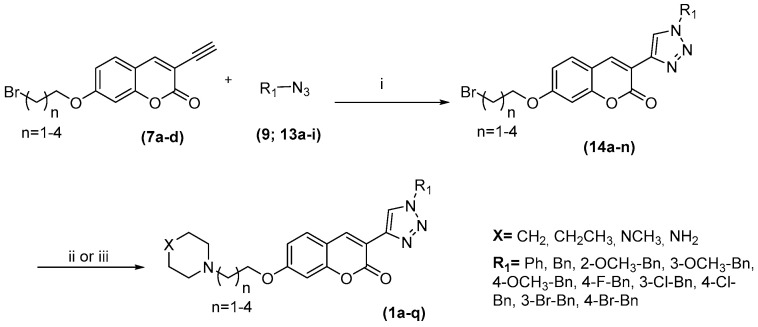
Synthesis of designed 7-alcoxyamino-3-(1,2,3-triazole)-coumarin derivatives (**1a**–**q**). Reagents and conditions: (i) CuSO_4_.5H_2_O, ascorbic acid, r.t., 24 h, 10–79%; (ii) piperidine or methyl piperidine or methyl piperazine, acetonitrile, 60 °C, 4 h, 40–94% or (iii) (1) piperazine-Boc, acetonitrile, 60 °C, 4 h, 83%; (2) TFA, dichloromethane, r.t, 24 h, 60%.

Then, a 1,3-dipolar cycloaddition reaction catalyzed by Cu(I) (“click chemistry”) was performed between the alkynes (**7a**–**d**) and the phenyl/benzyl azides (**9**, **13a**–**i**), leading to the formation of the 1,2,3-triazole heterocycles (**14a**–**n**). Finally, an amination reaction was performed in the alkyl chain with different cyclic amines, leading to the final compounds (**1a**–**q**) (Scheme 3).

### 2.2. Evaluation of Anticholinesterase Activity

The anticholinesterase activity of the compounds was evaluated using the Ellman method [23], with donepezil as the reference compound. Initial screening of compounds **1a**–**g** revealed potent *Ee*AChE inhibition, with IC_50_ values ranging from 50.0 nM to 4790 nM. In contrast, lower inhibitory potency was observed against *Eq*BChE, with IC_50_ values between 2414 nM and 11,130 nM (Table 1).

Analysis of compounds **1a**–**d** showed a decreasing *Ee*AChE inhibitory potency and selectivity with increasing alkyl chain length, with a two-methylene spacer providing the most favorable linker, consistent with previous reports for the prototype derivative (**2**) [22]. Conversely, increasing the spacer length led to improved inhibitory activity against BChE. Regarding changes in the cationic moiety nature (cyclic amines) (**1a**, **1e**–**g**), the unsubstituted piperidine derivative (**1a**) exhibited the best potency and selectivity toward *Ee*AChE. Despite the presence of the optimal linker, modifications in this region resulted in a significant reduction in *Ee*AChE inhibitory activity, likely due to shifts in the cyclic amine cationic moiety within the CAS (discussed in Section 2.3.2), an important pharmacophore point.

Following the optimization of the 7-aminoalkoxy-coumarin moiety resulting in compound **1a**, we investigated its benzyl analogue **1h**, to assess the influence of increased conformational flexibility in the PAS region. This modification significantly enhanced *Ee*AChE inhibition (IC_50_ = 6.03 nM) and selectivity over *Eq*BChE (IC_50_ = 3790 nM; SI = 632) when compared to **1a** (*Ee*AChE IC_50_ = 50.02 nM; *Eq*BChE IC_50_ = 8660 nM; SI = 173). Worth note, the reference drug donepezil showed an IC_50_ of 7.02 nM for *Ee*AChE and SI over *Eq*BChE of 341, highlighting the excellent results obtained for **1h**. The influence of this higher degree of freedom at molecular level is best discussed in Section 2.3.2.

Evaluation of benzyl substituted derivatives **1i**–**q** furnished notably potent cholinesterase inhibitors. The methoxylated derivatives **1j** (*Ee*AChE IC_50_ = 4.16 nM, *meta*-substituted) and **1k** (*Ee*AChE IC_50_ = 4.58 nM, *para*-substituted) demonstrated the strongest AChE inhibition, with compound **1j** also showing the highest selectivity (*Eq*BChE IC_50_ = 2880 nM; SI = 686). All data are summarized in Table 1.

The *Ee*AChE is a commonly used, readily available, and cost-effective alternative to human acetylcholinesterase (*h*AChE) for screening AChE inhibitors and for initial evaluations. However, due to interspecies differences and potential translational implications, compounds **1h**, **1j**, and **1q** were selected for testing against *h*AChE. The inhibitory potencies for *h*AChE are presented in Table 1, with activities for most of the chosen compounds aligning with the same order of magnitude as their *Ee*AChE inhibition.

### 2.3. Mechanism of Cholinesterase Inhibitions

#### 2.3.1. Kinetic Studies

**Figure 2:**
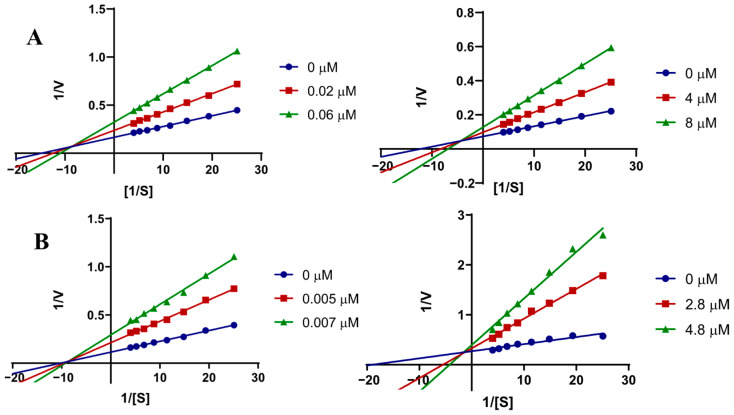
Lineweaver -Burk plots of the prevention kinetics in *Ee*AChE (**left**) and *Eq*BChE (**right**) of compounds **1a** (**A**) and **1h** (**B**).

To investigate the inhibition mechanism of AChE and BChE enzymes, compounds **1a** and **1h** were selected for detailed analysis. The Lineweaver-Burk plots, derived from the Michaelis-Menten equation, along with the kinetic parameters V_max_ and K_m_, were used to evaluate the inhibition profiles. The kinetic studies revealed that all studied compounds exhibit mixed-type inhibition mechanisms (decreased V_max_, increased K_m_, intersection of the straight lines in the 2nd quadrant of the Cartesian system) (Table 2 and Table S2, Figure 2).

These findings indicate that the inhibitors are able to bind to both the catalytic site (competitive inhibition), as well as the allosteric site (non-competitive inhibition) in AChE and BChE. Based on these results, the competitive (K_i_) and non-competitive (K_i’_) affinity constants were determined for compounds **1a**, **1h** (Table 2 and Table S2, Figure 2) and **1j** (Tables S2 and S3). Despite not yet confirmed, the mixed-type inhibition of cholinesterases can be interesting, as this mode of action was previously postulated to reduce β-amyloid peptide aggregation [25], suggesting an interesting profile for our compounds.

#### 2.3.2. Molecular Docking Studies

To better understand the compounds’ inhibitory effects on the enzymes, molecular docking simulations were performed for all compounds against *Ee*AChE and *Eq*BChE using the GoldScore fitness function in the GOLD program, as previously described [26]. The molecular docking studies with *Ee*AChE (PDB:1C2B) demonstrated that the inhibitors can occupy the CAS and PAS simultaneously, in agreement with the data obtained in the enzymatic kinetics study. Docking results for *Ee*AChE and *Eq*BChE are presented in Table S3 in Supplementary Material.

**Figure 3:**
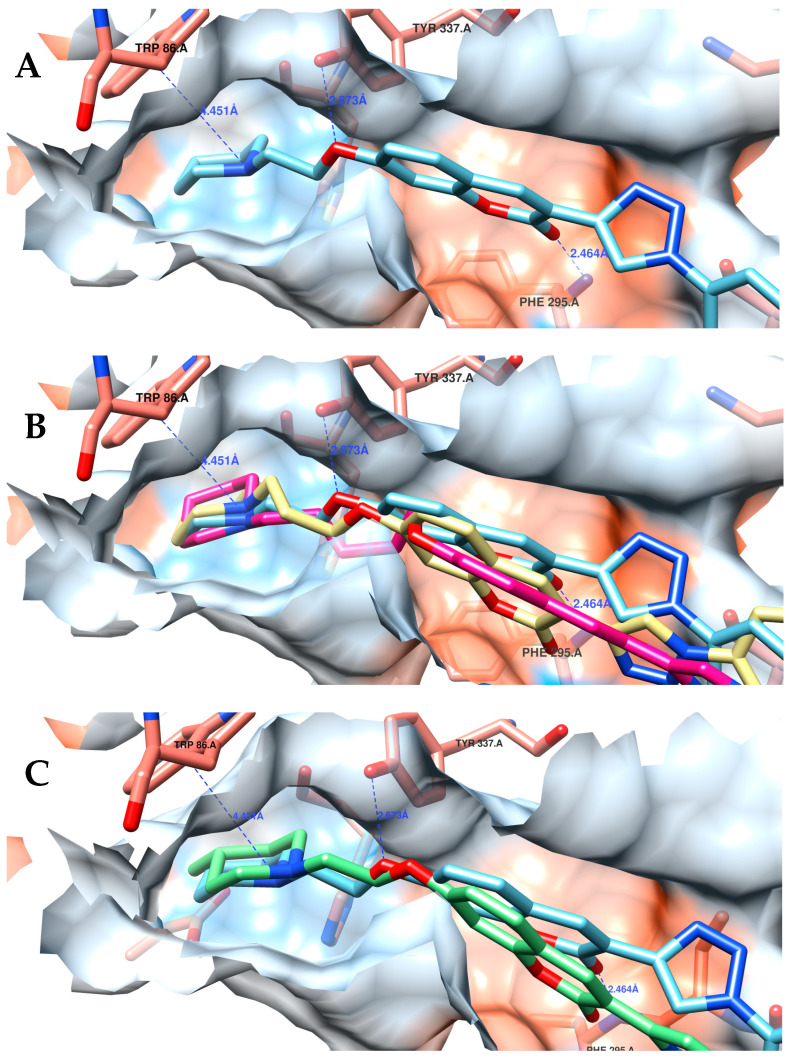
(**A**)—Key molecular interactions of compound **1a** (light blue) within the *Ee*AChE cavity. (**B**)—Superposition of compound **1a** with **1b** (3-methylene linker, light yellow) and **1d** (5-methylene linker, pink). (**C**)—Superposition of **1a** with methyl-substituted derivative **1e** (4-methyl-piperazinyl, green) in the *Ee*AChE binding site.

In the CAS, compounds containing a protonated piperidinyl group (**1a**–**d**, **1h**–**q**) are engaged in strong cation–π interactions with the Trp86 residue. Meanwhile, the remaining 7-alkoxy-coumarin moiety is oriented toward the enzyme’s main entrance (Figure 3A). Additionally, the two-methylene linker positions the coumarin nucleus within the active site channel, enabling hydrogen bonding interactions with the Tyr337 and Phe295 residues, while larger linkers torsion in an unnatural way the alcoxy-piperidinyl moiety (3-methylene) or hampered important interactions with the Tyr337 and Phe295 (4- or 5-methylene) (Figure 3B). For the 4-methyl-substituted (4-methyl-pyperidinyl (**1e**) and -piperazinyl (**1f**)) derivatives, adding a bulky group at this pharmacophoric region disrupts the cation–π interaction with the Trp86 and Tyr337 residues, shifting it away from the central binding site (Figure 3C).

**Figure 4:**
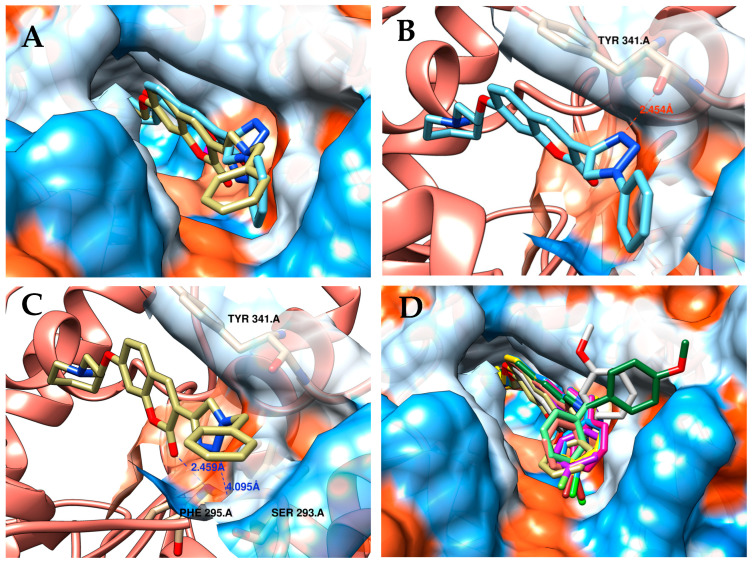
(**A**)—Coumarin derivatives **1a** (light blue) and **1h** (gold) docked within the cavity of the *Ee*AChE enzyme (PDB:1C2B), represented as a Van der Waals surface. (**B**)—Representation of conformational constraint of compound **1a** positioning the nitrogen groups through the carbonyl of Tyr341. (**C**)—Representation of compound **1h** and its additional interaction with Ser293. (**D**)—Structural superimposed of compounds **1h**–**1p** within the cavity of the *Ee*AChE enzyme.

In the PAS, the conformational freedom of the aromatic system linked to the coumarin nucleus significantly influences inhibitory potency. The constrained phenyl-1,2,3-triazole system in **1a** is confined to hydrophobic interactions within the cavity by the phenyl group (Figure 4A). Additionally, due to its rigidity, the 1,2,3-triazole group must rotate to fit within the PAS, adopting an orthogonal orientation relative to the coumarin nucleus and positioning its nitrogen atoms near the carbonyl group of Tyr341 at a distance of 2.45 Å (Figure 4B). However, the sp^3^ carbon in the benzyl group (**1h**) confers greater conformational flexibility to the triazole, enabling optimal orientation for hydrogen bonding with residue of Ser293 (Figure 4C). Furthermore, this flexibility allows the phenyl ring to occupy a hydrophobically favorable region of the PAS, accommodating all benzyl compounds, a feature reflected in the enhanced activity observed for most benzyl derivatives (Figure 4D).

**Figure 5:**
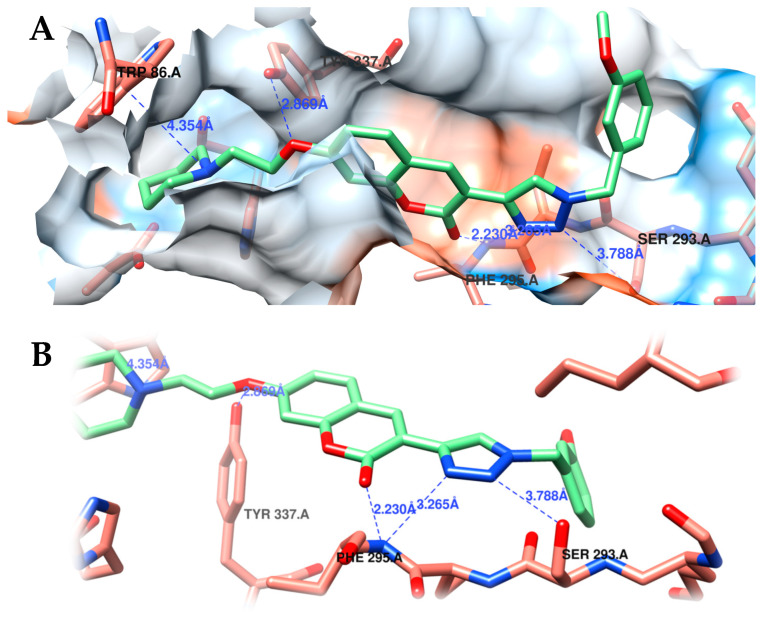
(**A**)—Key molecular interactions of compound **1h** within the *Ee*AChE cavity. (**B**)—Details of new H-bond interactions performed by **1j**, allowed by the conformational freedom of 1,2,3-triazol.

Besides the better fitness of benzyl group in the PAS, additional interactions were observed in the docking results for this superior homologue series, illustrated by compound **1j**, the most potent and selective compound (Figure 5A). In the CAS, compound **1j** is still performing the same interactions of other 7-alkoxypiperidinyl-coumarins, i.e., the protonated piperidinyl group engaged in strong cation–π interaction with the Trp86 residue, and hydrogen bonding interactions with the Tyr337 and Phe295 residues. In compound **1a**, the 1,2,3-triazole group is oriented toward an unfavorable region; however, in **1j**, the optimal positioning of this aza-heterocycle directs its nitrogen atoms toward Ser293 and Phe295, enabling the formation of two additional hydrogen bonds at distances of 3.79 Å and 3.26 Å, respectively (Figure 5B).

Despite small differences can be observed between *Ee*AChE and *Ee*AChE (Figures S15 and S16) in the CAS and PAS regions, similar pose and interaction profile (specially with Trp86) was observed for **1j,** when evaluated in *h*AChE,. Compound **1j** poses are very close to those in *Ee*AChE, showing some differences at the entrance of *h*AChE gorge, maybe due to the flexibility of benzyl ring (Figure S17).

For the enzyme *Eq*BChE, it has been demonstrated that inhibitors can occupy the CAS and PAS simultaneously. The comparison between inhibition profiles and docking results supports the influence of the BChE cavity volume on compound activity. The larger dimension of the BChE active site, while allowing the entry of bulkier ligands, compromises the formation of directional and stable interactions. This reduced efficiency in molecular interactions directly explains the lower inhibitory potency observed in vitro for BChE compared to AChE.

### 2.4. Cytotoxicity and In Vitro Neuroprotection Assay in SH-SY5Y Neuroblastoma Cells

**Figure 6:**
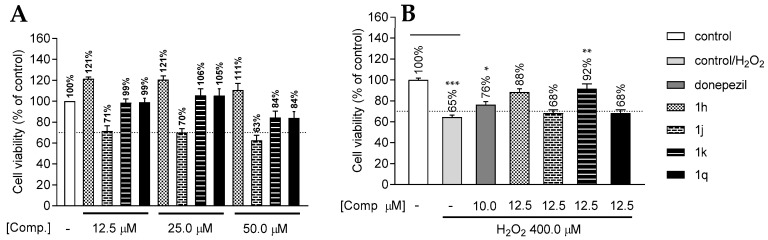
(**A**)—Cell viability (%) was accessed from pre-incubated SH-SY5Y cells with 12.5, 25.0 and 50.0 μM of compounds **1h**, **1j**, **1k** e **1q**. Results represent the average of 9 replicates (*n* = 9). (**B**)—Neuroprotective effects of compounds **1h**, **1j**, **1k** e **1q** (12.5 µM) in H_2_O_2_-damaged SH-SY5Y cells. Cells were pre-incubated with 12.5 μM of compounds or 10 μM of standard drug donepezil for 2 h and then exposed to H_2_O_2_ (400 μM) for 24 h. For both assays, cell viability was assessed using the MTT assay. Data are expressed as mean ± standard deviation (SD); (n = 9). (* *p* < 0.05; ** *p* < 0.01; *** *p* < 0.001). Statistical comparisons were performed between each compound and the control experiment with H_2_O_2_.

Safety assessment is essential in the development of new bioactive compounds. Much of the failure in the process of discovering new medicines (clinical trials) would be attributed to insufficient safety profiles [27]. Thus, neurotoxicity evaluations using the immortalized neuroblastoma cell line SH-SY5Y at concentrations of up to 50 μM (12.5, 25.0 and 50 μM) were performed (Figure 6A). The most potent AChE inhibitors, i.e., **1h**, **1k**, **1j** and **1q**, presented profiles considered non-neurotoxic up to 25 μM, as cell viability remained above or equal to 70%. Only **1j** reduced cell viability below this threshold (63%), and only at 50 μM. Furthermore, compounds **1h**, **1k** and **1q** presented cell viability at 50 μM comparable to pharmacological standards such as donepezil (80%) and tacrine (97%), tested under the same conditions [28,29].

Neurodegeneration is a key contributor to the cognitive and motor impairments observed in AD and is characterized by the progressive neuronal loss and dysfunction in the central nervous system (CNS). To investigate potential neuroprotective agents, alternative in vitro models have been developed to evaluate the efficacy of small molecules in preventing neuronal damage. One commonly used model involves neuronal cell lines exposed to exogenous oxidative stressors, such as hydrogen peroxide (H_2_O_2_), which induce an imbalance between the production and elimination of reactive oxygen species (ROS). The same compounds tested for neurotoxicity were evaluated for preventing neuronal cell death induced by H_2_O_2_ [30].

In this assay, the addition of H_2_O_2_ (400 µM) to the culture medium induced significant cell death, as demonstrated by a 65% reduction in cell viability compared to the control medium (DMEM:F12) (Figure 6B).

The reference drug and positive control donepezil [31], at 10 µM, increased cell viability by 11% (to 76%). Notably, **1h** and **1k,** demonstrated interesting neuroprotective ability, increasing cell viability by 23% (cell viability of 88%) and 27% (cell viability of 92%), respectively (Figure 6B). Although the compounds were inactive in DPPH and ORAC assays, ruling out direct radical scavenging, the neuroprotection is likely to involve indirect mechanisms, such as the activation of cellular defense pathways or the modulation of specific targets implicated in the oxidative stress response [22,32].

### 2.5. In Silico ADMET Physicochemical Profile Analysis

In order to evaluate the drug-likeness of tested compounds, the pharmacokinetic (PK) profile of the novel 1,2,3-triazole-coumarin derivatives (**1a**–**r**) was assessed via in silico studies on the on-line platform SwissADME [33,34,35], revealing favorable ADME properties for most compounds.

**Figure 7:**
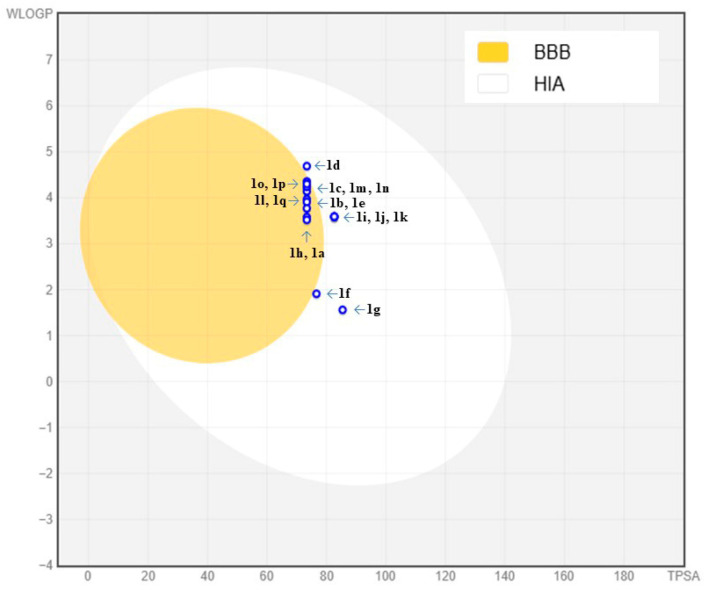
BOILED-Egg ADME model for coumarin compounds **1a**–**q** [35]. (HIA) gastrointestinal absorption; (BBB) brain penetration.

Key parameters analyzed included topological polar surface area (TPSA: 59.98–85.42 Å^2^), consensus Log P (2.5–4.60), LogS (−4.02 to −5.84, moderately soluble range), human intestinal absorption (HIA), blood–brain barrier (BBB) permeability, and drug-like similarity (Table 3 and Tables S4–S20). According to the BOILED-Egg model [35], moderate polarity (TPSA < 85 Å^2^) and lipophilicity placed most derivatives within the Okyellow region (Table 3 and Figure 6), suggesting high gastrointestinal absorption and CNS accessibility, critical for CNS acting therapeutics. Considering the most promising compounds, i.e., **1a** and **1h**–**1q**, only three were predicted to lack BBB permeability (**1i**–**1k**); notably, all the methoxy-substituted compounds. All predicted compounds’ PK parameters are inside the pink area of Radar Graphic (Tables S11–S20), which is the optimal range area for drug-likeness properties. However, it is notorious that TPSA’s values higher than 75 Å^2^ can negative influence most of the predicted compounds’ ability of crossing the BBB, as predicted for **1f**–**1g** and **1i**–**1k** (Table 3 and Figure 7). The prediction of P-glycoprotein substrate activity (P-gp^+^) raised concerns regarding the pharmacokinetic profile of the series. This finding requires future experimental verification. Since the compounds are intended to act at the CNS level, confirmed P-gp^+^ activity would warrant structural modifications, such as reducing polarity, or the strategic use of P-gp inhibitors in in vivo studies [36,37].

Compounds **1a**–**b**, **1e**–**h**, and **1n** adhered to Lipinski’s [38], Ghose [39], Veber [40], Egan [41], and Muegge [42] drug-likeness rules without violations, underscoring their potential as drug candidates.

**Table 3:** In silico predicted ADMET physicochemical profile for compounds **1a**–**1q**.

Compound	TPSA (Å^2^)	LogP ^a^	LogS ^b^	GI Absorption ^c^	BBB Permeant ^d^
**1a**	73.39	3.62	−4.96	High	Yes
**1b**	73.39	3.91	−5.19	High	Yes
**1c**	73.39	4.24	−5.42	High	Yes
**1d**	73.39	4.60	−5.65	High	No
**1e**	73.39	3.87	−5.31	High	Yes
**1f**	76.63	2.70	−4.39	High	No
**1g**	85.42	2.50	−4.02	High	No
**1h**	73.39	3.63	−4.93	High	Yes
**1i**	82.62	3.64	−5.00	High	No
**1j**	82.62	3.63	−5.00	High	No
**1k**	82.62	3.63	−5.00	High	No
**1l**	73.39	4.04	−5.09	High	Yes
**1m**	73.39	4.22	−5.52	High	Yes
**1n**	73.39	4.19	−5.52	High	Yes
**1o**	73.39	4.30	−5.84	High	Yes
**1p**	73.39	4.27	−5.84	High	Yes
**1q**	73.39	3.99	−5.15	High	Yes

^a^ Consensus Log *P*_o/w_ from predicted values of Log *P*_o/w_ (iLOGP, XLOGP3, WLOGP, MLOGP and SILICOS-IT); ^b^ calculated by the ESOL model [43]; ^c^ included in physicochemical space for HGI permeation; ^d^ included in physicochemical space for highly probable BBB permeation.

### 2.6. Evaluation of Compounds as MTDLs: hH_3_R Ligands and MAO-B Inhibitors

Our group recognized similarities between the structures of compounds described herein and pharmacophore models of H_3_R ligands [44]. These features are the cationic moiety (piperidinyl group) linked to a central core (coumarin) via a two- to three-methylene spacer and an arbitrary region (1,2,3-triazol group). Furthermore, the evaluation of MAO-A/B inhibition profiles is based on the fact that coumarins serve as scaffolds for MAO inhibition, while our compounds present aromatic groups at position 3, a region known to be critical for selective MAO-B inhibition [45].

Based on the ensemble of results, we decided to start with **1h** as lead compound (AChE IC_50_ = 6.03 nM), which is equipotent to compounds **1j** (IC_50_ = 4.16 nM) and **1k** (IC_50_ = 4.58 nM). Moreover, **1h** showed no neurotoxicity up to 50 µM, neuroprotection with increasing of 23% in cell viability (65 to 88%) and predicted good HIA and BBB crossing. On the other hand, **1j** and **1k** presented possible drawbacks in our studies such as lack of predicted BBB permeability for both compounds and neurotoxicity in high concentrations for **1j**.

**Figure 8:**
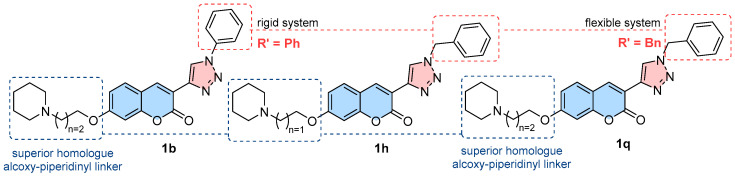
Compounds selected for evaluation as MTDLs.

We also decided to assay two other correlated compounds, **1b** and **1q.** Both compounds present closer similarity to the established H_3_R pharmacophore, i.e., three-methylene spacer (superior homologation in alcoxy-piperidinyl moiety), but with different flexibilities in 1,2,3-triazole moiety (Figure 8).

The affinity constants (*K_i_*) of compounds **1b**, **1h**, and **1q** for H_3_R were determined using a radioligand displacement assay [46], with pitolisant as reference compound. The *K_i_* values (Table 4) confirmed that all three compounds bind to H_3_R with *K_i_* values ranging from 32 to 558 nM. Clearly, the three-methylene spacer in **1b** (*K_i_* = 32 nM) and **1q** (*K_i_* = 151 nM) appears optimal for H_3_R interaction, consistent with literature reports [47]. Additionally, more rigid triazole moieties, as in **1b**, seem to enhance affinity since **1q** presented significantly lower H_3_R affinity (*K_i_* = 151 nM) (Table 4).

The inhibitory activity of the compounds against the MAO-A/B isoforms was evaluated using a fluorometric assay that quantifies hydrogen peroxide (H_2_O_2_), a byproduct of the MAO-catalyzed reaction, with rasagiline as the reference compound, es described previously [46]. Among compounds **1b**, **1h**, and **1q**, only compound **1q** demonstrated potent and selective inhibition of MAO-B, with an IC_50_ value of 1688 nM and a MAO-B/A selectivity ratio greater than 18-fold. This profile is pharmacologically relevant, as selective MAO-B inhibitors are prioritized in the treatment of neurodegenerative diseases, such as Alzheimer’s disease, due to their lower risk of side effects associated with MAO-A inhibition (e.g., serotonin syndrome).

Based on the results for cholinesterase and MAO inhibition, as well as H_3_R affinity, although the number of examples is limited (n = 3), it is possible to infer that variations in the substitution pattern of the compounds can lead to distinct activity profiles. For compound **1h** (n = 1, R = Bn), the primary effect is potent AChE inhibition (IC_50_ = 6.03 nM), with complementary H_3_R modulation (*K_i_* = 558 nM). In the case of compound **1q** (n = 2, R = Bn), a superior homolog of **1h**, the activity profile is inverted, showing a preference for H_3_R modulation (*K_i_* = 151 nM) while still maintaining complementary inhibitory activity against AChE (IC_50_ = 1950 nM) and MAO-B (IC_50_ = 1688 nM). Finally, compound **1b** (n = 2, R = Ph), and with reduced flexibility in the 1,2,3-triazole moiety, emerged as a promising hit, exhibiting excellent H_3_R affinity (*K_i_* = 32 nM) and good AChE inhibitory potency (IC_50_ = 1330 nM).

Taken together, these findings suggest that this series represents a valuable model for the development of MTDLs compounds for Alzheimer’s disease models, a concept that will be further explored in future investigations of our group.

## 3. Materials and Methods

### 3.1. Chemicals, Reagents, and Equipment

All reagents and solvents were supplied by Sigma-Aldrich (Saint Louis, MO, USA). To structurally characterize the chemical compounds, nuclear magnetic resonance spectra of hydrogen (^1^H NMR) and carbon (^13^C NMR) were acquired by a 400 MHz or 500 MHz Bruker Advance spectrometer, with DMSO-*d*_6_ or CDCl_3_ as solvents and TMS as an internal standard. High-performance liquid chromatography (HPLC) analysis was carried out using a Prominence-Shimadzu with a loop of 10 μL and column Phenomenex C18 (100 mm × 4.6 mm × 3 μm) in MeOH (70%): 30% H_2_O (0.1% HCO_2_H). Mass spectrometry was performed on the CG-MS 2020 (Shimadzu, Kyoto, Japan).

The melting points were recorded with a Fisatom model 431D fusiometer and are uncorrected. Thin-layer chromatography (TLC) with 0.25 mm thick Merk 60 F254 silica gel plates were used to follow reactions.

### 3.2. Synthesis of the New Compounds

#### 3.2.1. Synthetic Procedures and Characterization Data for Trimethylsilyl Ethynyl (**6a**–**d**)

In a sealed reaction tube, a mixture containing 3 mmol of 3-bromo-7-bromoalkoxycoumarin derivatives (**5a**–**d**), 0.12 mmol CuI, 0.14 mmol PdCl_2_(PPh_3_)_2_, and 6 mmol triethylamine was dissolved in 30 mL acetonitrile. The system was purged with N_2_ to establish an inert atmosphere and heated to 60 °C. Subsequently, 4.95 mmol of TMS-A was added, and the reaction mixture was stirred at this temperature for 2 h. After complete consumption of the **5a**–**d,** monitored by TLC, the solution was filtered through a sintered funnel with Celite and washed with ethyl acetate. The solvent was removed in a rotary evaporator, and the products were purified using an Isolera equipment (Biotage, Uppsala, Sweden, model ISO-4SV) with a 10 g silica gel cartridge eluted with a mixture of hexane: ethyl acetate in a concentration gradient ranging from 10 to 30% of the more polar solvent.

7-(2-Bromoethoxy)-3-((trimethylsilyl)ethynyl)-2*H*-chromen-2-one (**6a**). Beige solid. 54% yield. mp: 155–158 °C. ^1^H NMR (500 MHz, CDCl_3_): δ = 7.87 (s, 1H), 7.39 (d, J = 8.5 Hz, 1H), 6.9 (dd, J = 8.7, 2.5 Hz, ^1^H), 6.82 (d, J = 1.9 Hz, 1H), 4.37 (t, J = 6.00 Hz, 2H), 3.69 (t, J = 6.00 Hz, 2H), 0,29 (s, 9H). ^13^C NMR (125 MHz, CDCl_3_): δ = 161.9, 159.8, 155.4, 146.1, 113.2, 129.2, 113.6, 109.9, 101.8, 101.3, 98.5, 68.5, 28.5.

7-(3-Bromopropoxy)-3-((trimethylsilyl)ethynyl)-2*H*-chromen-2-one (**6b**). Light brown solid, 62% yield. mp: 105–109 °C. ^1^H NMR (500 MHz, CDCl_3_): δ = 7.85 (s, 1H), 7.36 (d, J = 8.5, 1H), 6.86 (dd, J = 8.6, 2.0 Hz, 1H), 6.82 (d, J = 1.9 Hz, 1H), 4.18 (t, J = 5.7 Hz, 2H), 3.61 (t, J = 6.1 Hz, 2H), 2.36 (m, J = 6.0 Hz, 2H), 0.27 (s, 9H). ^13^C NMR (125 MHz, CDCl_3_): δ = 162.1, 159.4, 155.0, 145.7, 128.5, 113.0, 112.3, 109.0, 101.2, 100.6, 98.1, 65.7, 31.6, 29.2.

7-(4-Bromobutoxy)-3-((trimethylsilyl)ethynyl)-2*H*-chromen-2-one (**6c**). Light yellow solid, 64% yield. mp: 141–143 °C. ^1^H NMR (500 MHz, CDCl_3_): δ = 7.87 (s, 1H), 7.36 (d, J = 8.5, 1H), 6.86 (dd, J1 = 8.7, J2 = 2 Hz, 1H), 6.80 (d, J = 2 Hz, 1H), 4.08 (t, J = 5.7 Hz, 2H), 3.51 (t, J = 6.5 Hz, 2H), 2.12–2.07 (m, J = 6.5 Hz, 2H), 2.07–2.02 (m, 2H), 0.28 (s, 9H). ^13^C NMR (125 MHz, CDCl_3_): δ = 162.6, 159.7, 155.3, 146.1, 128.8, 113.5, 112.4, 109.1, 101.2, 100.7, 98.4, 67.6, 33.1, 29.2, 27.6, 20.2

7-((5-Bromopentyl)oxy)-3-((trimethylsilyl)ethynyl)-2*H*-chromen-2-one (**6d**). Light brown solid, 71% yield. mp: 73–76 °C. ^1^H NMR (500 MHz, CDCl_3_): δ = 7.85 (s, 1H), 7.34 (d, J = 8.5, 1H), 6.84 (dd, J = 8.7, 2.4 Hz, 1H), 6.78 (d, J = 2.2 Hz, 1H), 4.03 (t, J = 6.30 Hz, 2H), 3.45 (t, J = 6.60 Hz, 2H), 1.95 (m, 2H), 1.86 (m, 2H), 1.68–1.63 (m, 2H), 0.27 (s, 9H). ^13^C NMR (125 MHz, CDCl_3_): δ = 162.7, 159.7, 155.4, 146.1, 128.7, 113.5, 112.4, 109.0, 101.2, 100.7, 98.5, 68.3, 33.4, 32.3, 28.1, 24.7.

#### 3.2.2. Synthesis Procedure for Deprotection (**7a**–**d**)

To a solution of compound **6a**–**d** (1mmol) in methanol (15mL), K_2_CO_3_ (1.53 mmol) was added. The reaction mixture was stirred for 30 min at the room temperature. Next the reaction mixture was concentrated on the rotary evaporator, the residue was dissolved in 20 mL of ethyl acetate, the organic layer was washed with water (3 × 20 mL), dried over sodium sulfate and evaporated.

#### 3.2.3. Synthesis Procedures for Phenyl Azide (**9**)

In a reaction tube, a solution containing 5.4 mmol of arylamine (**8**), 10 mL of ethyl acetate, and 1.25 mL of water was prepared, cooled to 0 °C, and treated with 3 mL of concentrated hydrochloric acid under magnetic stirring for 10 min. In a separate 25 mL beaker, 9.2 mmol of NaNO_2_ was dissolved in 2 mL of water and added to the reaction mixture over 3 min; the resulting mixture was stirred at 0 °C for 30 min. A pre-prepared solution of 9.2 mmol of NaN_3_ in 2 mL of water was then added dropwise over 5 min, followed by stirring for 1 h while allowing the temperature to rise gradually to room temperature. The reaction was diluted with 12.5 mL of water and extracted with ethyl acetate (2 × 12.5 mL). The combined organic phase was washed sequentially with 12.5 mL of 0.1 M sodium hydroxide solution and 12.5 mL of water, dried over anhydrous Na_2_SO_4_, and concentrated in vacuo to afford azide **9**.

#### 3.2.4. Synthetic Procedures for Benzyl Azides (**13a**–**i**)

Step1: benzyl alcohols (**11a**–**i**):

In a reaction tube, NaBH_4_ (1.5 mmol) was added to a solution of benzylic aldehyde (**10a**–**i**) (1 mmol) in EtOH (1 mL) at 0 °C. The mixture was allowed to warm to room temperature and stirred. After complete consumption of the aldehyde starting material, the reaction was quenched with 10% NaOH and stirred for an additional ~10 min. Then, the crude mixture was evaporated under vacuum to remove EtOH and the remaining aqueous mixture was extracted with dichloromethane (3 ×). Finally, the combined organic layer was dried over sodium sulfate and evaporated. The alcoholic product obtained was taken to the next additional purification step.

Step2: benzyl bromides (**12a**–**i**):

To a solution of benzyl alcohols (**11a**–**i**) (1.75 mmol) in dichloromethane (8.6 mL), carbon tetrabromide (1.75 mmol) and triphenyl phosphine (1.75 mmol) were added at room temperature. The reaction mixture was stirred for 2 h and then the solvent was removed by evaporation under reduced pressure. The product obtained was taken to the next additional purification step.

Step3: benzyl azides (**13a**–**i**):

To a solution of the corresponding benzyl bromide (**12a**–**i**) (1.75 mmol) in water/acetone (1:3, 0.25 M), NaN_3_ (3.5 mmol) was added. The reaction mixture was stirred for 30 min at the room temperature. Then, the mixture was diluted with DCM and washed with water. The organic phase was dried with anhydrous Na_2_SO_4_ and the solvent removed in a evaporation under vacuum. The products were purified using an Isolera equipment (Biotage, model ISO-4SV) with a 10 g silica gel cartridge eluted with a mixture of hexane: ethyl acetate in a concentration gradient ranging from 10 to 30% of the more polar solvent.

#### 3.2.5. Synthetic Procedures for Triazoles (**14a**–**m**)

In a 25 mL reaction tube, 1 mmol of 3-(ethenyl)-7-(bromoalkoxy)coumarin (**7a**–**d**) and 1.3 mmol of arylazide (**9**, **13a**–**i**):were dissolved in a 1:1 *v*/*v* water/ethanol mixture. A pre-prepared solution of 0.2 mmol sodium ascorbate in 2 mL water was added, followed by a solution of 0.05 mmol CuSO_4_·5H_2_O (7.5% *w*/*v* in 2 mL water). The reaction was stirred vigorously for 24 h under dark conditions at room temperature. After completion, the ethanol was removed under reduced pressure, and the residue was poured onto ice. The resulting solid was vacuum-filtered and washed with ice-cold water. The crude products (**14a**–**m**) were purified via flash column chromatography (Isolera system, silica gel stationary phase) using a hexane/ethyl acetate gradient (40–70% ethyl acetate) as the mobile phase.

7-(2-Bromoethoxy)-3-(1-phenyl-1*H*-1,2,3-triazol-4-yl)-2*H*-chromen-2-one **14a**. Light brown solid, 61% yield. mp: 222–224 °C. ^1^H NMR (500 MHz, CDCl_3_): δ = 8.74 (s, 1H), 8.71 (s, 1H), 7.77 (d, J = 7.3 Hz, 1H), 7.53–7.48 (m, 4H), 7.40 (t, 1H), 6.89 (d, J = 8.2 Hz, 1H), 6.82 (s, 1H), 4.32 (m, 2H), 3.63 (m, 2H). ^13^C NMR (125 MHz, CDCl_3_): δ = 160.5, 159.8, 158.4, 146.5, 136.9, 130.6, 129.8, 129.7, 128.9, 121.4, 120.6, 114.8, 113.5, 101.4, 68.3, 28.4.

7-(3-Bromopropoxy)-3-(1-phenyl-1*H*-1,2,3-triazol-4-yl)-2*H*-chromen-2-one **14b**. Brown solid, 63% yield. mp: 192–193 °C. ^1^H NMR (500 MHz, CDCl_3_): δ = 8.83 (s, 1H), 8.80 (s, 1H), 7.98 (m, 4H), 7.85 (d, J = 7.6 Hz, 1H), 7.49 (t, 1H), 6.95 (d, J = 8.8 Hz, 1H), 6.93 (s, 1H), 4.32 (t, 2H), 3.65 (t, 2H), 2.40 (m, 2H). ^13^C NMR (125 MHz, CDCl_3_): δ = 161.7, 159.6, 156.9, 137.8, 136.7, 129.5, 129.3, 128.5, 121.0, 120.2, 114.3, 113.2, 112.9, 101.0, 65.7, 31.7, 29.3.

7-(4-Bromobutoxy)-3-(1-phenyl-1*H*-1,2,3-triazol-4-yl)-2*H*-chromen-2-one **14c**. Light brown solid, 79% yield. mp: 173–175 °C. ^1^H NMR (500 MHz, CDCl_3_): δ = 8.81 (s, 1H), 8.78 (s, 1H), 7.84 (d, J = 7.9 Hz, 1H), 7.58–7.55 (m, 4H), 7.47 (t, J = 7.3 Hz, 1H), 6.92 (d, J = 8.8 Hz, 1H), 6.88 (sl, 1H), 4.1 (t, J = 5.7 Hz, 2H), 3.52 (t, J = 6.3 Hz. 2H), 2.14–2.08 (m, 2H), 2.04–2.08 (m, 2H). ^13^C NMR (125 MHz, CDCl_3_): δ = 169.2, 159.9, 155.0, 141.6, 138.1, 136.9, 129.8, 129.5, 128.8, 121.2, 120.5, 114.4, 113.5, 113.0, 101.0, 67.6, 33.2, 29.3, 27.6.

7-((5-Bromopentyl)oxy)-3-(1-phenyl-1*H*-1,2,3-triazol-4-yl)-2*H*-chromen-2-one **14d**. Light brown solid, 61% yield. mp: 175–178 °C. ^1^H NMR (500 MHz, CDCl_3_): δ = 8.78 (s, 1H), 8.81 (s, 1H), 7.85 (d, J = 6.9 Hz, 1H), 7.56 (m, 4H), 7.47 (m, 1H), 6.93 (d, J = 7.6 Hz, 1H), 6.88 (s, 1H), 4.07 (t, 2H), 3.47 (t, 2H), 1.97 (m, 2H), 1.88 (m, 2H), 1.26 (sl, 2H). ^13^C NMR (125 MHz, CDCl_3_): δ = 162.6, 160.2, 155.3, 138.4, 137.2, 130.0, 129.7, 129.0, 121.5, 120.7, 114.6, 113.8, 113.1, 101.3, 68.5, 33.7, 32.6, 28.4, 25.0.

3-(1-Benzyl-1*H*-1,2,3-triazol-4-yl)-7-(2-bromoethoxy)-2*H*-chromen-2-one **14e**. Dark brown oil, 57% yield. ^1^H NMR (500 MHz, CDCl_3_): δ = 8.62 (s, 1H), 8.26 (s, 1H), 7.54 (d, J = 8.8 Hz, 1H), 7.35–7.31 (m, 4H), 7.25 (m, 1H), 6.90 (d, J_1_ = 8.5, 2.2 Hz, 1H), 6.82 (s, J = 1.9 Hz, 1H), 5.52 (s, 2H), 4.33 (t, 2H), 3.64 (t, 2H). ^13^C NMR (125 MHz, CDCl_3_): δ = 161.0, 159.6, 154.4, 140.9, 137.5, 134.1, 129.3, 128.8, 128.5, 127.7, 123.2, 114.4, 113.1, 113.1, 101.0, 67.9, 54.0, 28.1.

7-(2-Bromoethoxy)-3-(1-(2-methoxybenzyl)-1*H*-1,2,3-triazol-4-yl)-2*H*-chromen-2-one **14f**. Yellow solid, 46% yield. mp: 180–182 °C. ^1^H NMR (500 MHz, CDCl_3_) δ = 8.67 (s, 1H), 8.32 (s, 1H), 7.53 (d, J = 8.6 Hz, 1H), 7.34 (t, J = 7.3 Hz, 1H), 7.25 (t, J = 7.3 Hz, 1H), 6.99–6.88 (m, 1H), 6.85 (d, J = 2.1 Hz, 1H), 5.60 (s, 2H), 4.36 (t, J = 6.2 Hz, 2H), 3.89 (s, 3H), 3.68 (t, J = 6.1 Hz, 2H). ^13^C NMR (125 MHz, CDCl_3_): δ = 161.4, 160.1, 157.6, 155.2, 137.6, 130.8, 130.7, 129.9, 124.3, 123.3, 121.3, 115.9, 114.0, 113.7, 111.2, 101.8, 77.2, 68.6, 55.9, 55.8, 49.7, 28.8.

7-(2-Bromoethoxy)-3-(1-(3-methoxybenzyl)-1*H*-1,2,3-triazol-4-yl)-2*H*-chromen-2-one **14g**. White solid, 37% yield. mp: 188–191 °C. ^1^H NMR (500 MHz, CDCl_3_) δ = 8.68 (s, 1H), 8.28 (s, 1H), 7.51 (d, J = 8.7 Hz, 1H), 7.29 (t, J = 7.9 Hz, 1H), 6.89 (dd, J = 14.2, 7.2 Hz, 3H), 6.84 (d, J = 14.0 Hz, 2H), 5.54 (s, 2H), 4.17 (t, J = 5.9 Hz, 2H), 3.78 (s, 3H), 2.81 (t, J = 5.9 Hz, 2H). ^13^C NMR (125 MHz, CDCl_3_): δ = 162.5, 160.5, 160.3, 155.3, 141.8, 138.1, 136.4, 130.6, 129.7, 123.8, 120.7, 115.1, 114.7, 114.0, 113.4, 101.6, 77.7, 77.4, 77.2, 77.2, 67.0, 55.7, 55.5, 26.3.

7-(2-Bromoethoxy)-3-(1-(4-methoxybenzyl)-1*H*-1,2,3-triazol-4-yl)-2*H*-chromen-2-one **14h**. Beige solid, 21% yield. mp: 197–200 °C. ^1^H NMR (500 MHz, CDCl_3_): δ = 8.68 (s, 1H), 8.25 (s, 1H), 7.54 (d, J = 8.7 Hz, 1H), 7.28–7.22 (m, 2H), 6.93–6.89 (m, 3H), 6.85 (d, J = 2.0 Hz, 1H), 5.55 (s, 2H), 4.36 (t, J = 6.1 Hz, 2H), 3.81 (s, 3H), 3.68 (t, J = 6.1 Hz, 2H). ^13^C NMR (125 MHz, CDCl_3_): δ = 161.3, 160.1, 159.8, 155.0, 141.4, 137.5, 129.8, 129.7, 126.7, 123.4, 115.5, 114.7, 113.7, 113.5, 101.6, 68.4, 55.5, 54.0, 28.5.

7-(2-Bromoethoxy)-3-(1-(4-fluorobenzyl)-1*H*-1,2,3-triazol-4-yl)-2*H*-chromen-2-one **14i**. Yellow solid, 10% yield. mp: 193–196 °C. ^1^H NMR (500 MHz, CDCl_3_): δ = 8.69 (s, 1H), 8.29 (s, 1H), 7.55 (d, J = 8.6 Hz, 1H), 7.31 (dd, J = 8.3, 5.3 Hz, 1H), 7.07 (t, J = 8.5 Hz, 1H), 6.93 (dd, J = 8.6, 2.2 Hz, 1H), 6.86 (d, J = 2.0 Hz, 1H), 5.55 (s, 2H), 4.37 (t, J = 6.1 Hz, 1H), 3.68 (t, J = 6.1 Hz, 1H). ^13^C NMR (125 MHz, CDCl_3_): δ = 161.2, 159.7, 154.8, 141.4, 137.6, 130.0, 130.0, 129.6, 123.3, 116.3, 116.1, 115.1, 113.5, 113.4, 101.4, 77.3, 77.0, 76.8, 68.2, 53.6, 28.4.

7-(2-Bromoethoxy)-3-(1-(3-chlorobenzyl)-1*H*-1,2,3-triazol-4-yl)-2*H*-chromen-2-one **14j**. Beige solid, 12% yield. mp: 177–179 °C. ^1^H NMR (500 MHz, CDCl_3_) δ = 8.70 (s, 1H), 8.31 (s, 1H), 7.55 (d, J = 8.6 Hz, 1H), 7.33–7.30 (m, 3H), 7.19 (d, J = 6.7 Hz, 1H), 6.93 (dd, J = 8.6, 2.2 Hz, 1H), 6.86 (d, J = 1.8 Hz, 1H), 5.56 (s, 2H), 4.37 (t, J = 6.0 Hz, 1H), 3.68 (t, J = 6.1 Hz, 1H). ^13^C NMR (125 MHz, CDCl_3_): δ = 161.4, 159.9, 155.0, 141.7, 137.8, 136.6, 135.2, 130.6, 129.8, 129.3, 129.2, 128.3, 126.2, 123.6, 115.2, 113.6, 113.6, 101.5, 68.4, 53.7, 28.4.

7-(2-Bromoethoxy)-3-(1-(4-chlorobenzyl)-1*H*-1,2,3-triazol-4-yl)-2*H*-chromen-2-one **14k**. Yellow solid, 44% yield. mp: 177–179 °C. ^1^H NMR (500 MHz, CDCl_3_): δ = 8.69 (s, 1H), 8.29 (s, 1H), 7.55 (d, J = 8.6 Hz, 1H), 7.35 (d, J = 8.3 Hz, 2H), 7.28–7.22 (m, 2H), 6.93 (dd, J = 8.6, 2.1 Hz, 2H), 6.86 (d, J = 1.9 Hz, 1H), 5.55 (s, 2H), 4.37 (t, J = 6.1 Hz, 3H), 3.68 (t, J = 6.1 Hz, 4H). ^13^C NMR (125 MHz, CDCl_3_): δ = 161.1, 159.6, 154.7, 141.4, 137.5, 134.8, 132.9, 129.5, 129.3, 123.3, 115.0, 113.4, 113.3, 101.3, 77.2, 68.2, 53.4, 28.2.

7-(2-Bromoethoxy)-3-(1-(3-bromobenzyl)-1*H*-1,2,3-triazol-4-yl)-2*H*-chromen-2-one **14l**. Yellow solid, 12% yield. mp: 185–189 °C. ^1^H NMR (500 MHz, CDCl_3_): δ = 8.73 (s, 1H), 8.34 (s, 1H), 7.58 (d, J = 8.6 Hz, 1H), 7.51 (d, J = 7.0 Hz, 1H), 6.96 (dd, J = 8.6, 2.3 Hz, 1H), 6.88 (d, J = 2.1 Hz, 1H), 5.58 (s, 2H), 4.39 (t, J = 6.1 Hz, 2H), 3.71 (t, J = 6.1 Hz, 2H). ^13^C NMR (125 MHz, CDCl_3_): δ = 161.1, 159.6, 154.7, 141.4, 137.6, 136.6, 131.9, 130.9, 130.6, 129.5, 126.4, 123.4, 123.0, 114.9, 113.3, 113.3, 101.2, 77.2, 68.1, 53.4, 28.2.

7-(2-Bromoethoxy)-3-(1-(4-bromobenzyl)-1*H*-1,2,3-triazol-4-yl)-2*H*-chromen-2-one **14m**. Orange solid, 16% yield. mp: 211–214 °C. ^1^H NMR (500 MHz, CDCl_3_): δ = 8.70 (s, 1H), 8.30 (s, 1H), 7.74–7.39 (m, 3H), 7.20 (s, 2H), 6.93 (d, J = 7.8 Hz, 1H), 6.86 (s, 1H), 5.54 (s, 2H), 4.37 (s, 2H), 3.68 (s, 2H). ^13^C NMR (125 MHz, CDCl_3_): δ = 161.6, 160.1, 155.2, 141.9, 138.1, 133.9, 132.7, 130.1, 130.0, 128.0, 123.8, 123.4, 115.4, 113.8, 101.7, 77.2, 68.6, 54.0, 28.8.

#### 3.2.6. Synthetic Procedures for Amines (**1a**–**q**)

In a borosilicate tube, 1 mmol of 3-(1-R-benzyl-1*H*-1,2,3-triazol-4-yl)-7-(bromoalkoxy) coumarin derivatives (**14a**–**m**) was dissolved in 15 mL acetonitrile, followed by the addition of 3 mmol cyclic amine (**15a**–**d**). The tube was sealed, and the mixture was stirred magnetically at 60 °C for 4 h. After complete consumption of the starting material (monitored by TLC; CH_2_Cl_2_/MeOH 10:1 *v*/*v*), the acetonitrile was evaporated under reduced pressure. The crude products were purified via flash column chromatography (Isolera system, silica gel stationary phase) using a CH_2_Cl_2_/MeOH gradient (0–20% MeOH) as the mobile phase.

3-(1-Phenyl-1*H*-1,2,3-triazol-4-yl)-7-(2-(piperidin-1-yl)ethoxy)-2*H*-chromen-2-one **1a**. Yellow solid, 53% yield. mp: 176–178 °C. ^1^H NMR (500 MHz, CDCl_3_): δ = 8.80 (s, 1H), 8.75 (s, 1H), 7.83 (d, 1H), 7.56 (m, 4H), 7.48 (t, 1H), 6.95 (d, 1H), 6.90 (s, 1H), 4.49 (sl, 2H), 3.23 (t, 2H), 2.98 (sl, 6H). ^13^C NMR (125 MHz, CDCl_3_): δ = 161.0, 159.7, 154.8, 141.5, 137.9, 136.9, 129.8, 129.7, 128.8, 121.3, 120.5, 114.9, 113.5, 113.0, 101.7, 70.5, 56.6, 54.5, 29.6, 22.6. HPLC purity: 98.8%.

3-(1-Phenyl-1*H*-1,2,3-triazol-4-yl)-7-(3-(piperidin-1-yl)propoxy)-2*H*-chromen-2-one **1b**. Brown solid, 63% yield. mp: 250–253 °C. ^1^H NMR (500 MHz, CDCl_3_): δ = 8.79 (s, 1H), 8.74 (s, 1H), 7.82 (d, J = 7.6 Hz, 1H), 7.58–7.53 (q, 4H), 7.46 (t, J = 7.3 Hz 1H), 6.9 (d, J = 7.9 Hz, 1H), 6.85 (sl, 1H), 4.19 (sl, 2H), 3.24 (sl, 2H), 2.73 (sl, 2H), 2.52 (sl, 2H), 2.29 (m, 2H). ^13^C NMR (125 MHz, CDCl_3_): δ = 161.4, 159.7, 154.8, 141.5, 138.0, 136.8, 129.8, 129.7, 128.8, 121.3, 120.4, 114.7, 113.4, 112.9, 101.4, 70.4, 65.7, 53.6, 29.6, 23.7, 22.5. HPLC purity: 95.6%.

3-(1-Phenyl-1*H*-1,2,3-triazol-4-yl)-7-(4-(piperidin-1-yl)butoxy)-2*H*-chromen-2-one **1c**. Yellow solid, 69% yield. mp: 190–192 °C. ^1^H NMR (500 MHz, CDCl_3_): δ = 8.76 (s, 1H), 8.69 (s, 1H), 7.78 (d, 1H), 7.56–7.50 (m, 4H), 7.42 (sl, 1H), 6.89 (d, 1H), 6.83 (sl, 1H), 4.06 (sl, 2H), 3.54 (sl, 2H), 3.05 (sl, 2H), 2.71–2.67 (t, 2H), 2.13–2.05 (m, 4H), 1.87 (sl, 6H). ^13^C NMR (125 MHz, CDCl_3_): δ = 162.0, 159.9, 154.8, 138.3, 136.7, 129.7, 129.6, 128.8, 121.3, 120.4, 114.2, 113.3, 113.0, 101.0, 67.5, 56.8, 53.1, 29.5, 26.1, 22.5, 21.8. HPLC purity: 96.1%.

3-(1-Phenyl-1*H*-1,2,3-triazol-4-yl)-7-((5-(piperidin-1-yl)pentyl)oxy)-2*H*-chromen-2-one **1d.** Brown solid, 76% yield. mp: 277–280 °C. ^1^H NMR (500 MHz, CDCl_3_): δ = 8.74 (s, 1H), 8.67 (s, 1H), 7.75 (sl, 1H), 7.54–7.48 (m, 4H), 7.41 (t, 1H), 6.86 (d, 1H), 6.81 (sl, 1H), 4.01 (sl, 2H), 3.29 (sl, 2H). ^13^C NMR (125 MHz, CDCl_3_): δ = 162.6, 160.7, 156.6, 138.6, 136.9, 129.9, 129.8, 129.1, 121.5, 120.6, 113.3, 114.8, 114.2, 101.2, 68.1, 57.2, 53.3, 29.7, 28.4, 23.5, 22.7, 22.0. HPLC purity: 95.2%.

7-(2-(4-Methylpiperidin-1-yl)ethoxy)-3-(1-phenyl-1*H*-1,2,3-triazol-4-yl)-2*H*-chromen-2-one **1e**. Orange solid, 52% yield. mp: 149–151 °C. ^1^H NMR (500 MHz, CDCl_3_): δ = 8.84 (s, 1H), 8.74 (s, 1H), 7.85 (d, J = 8.1, 1H), 7.65–7.58 (m, 4H), 7.51 (t, 1H), 6.99 (d, J = 8.5, 1H), 6.94 (s, 1H), 4.26 (sl, 2H), 3.66 (sl, 2H), 3.05 (d, 2H), 2.23 (t, 2H), 1.71 (d, 2H), 1.31 (t, 2H), 1.27 (s, 1H), 0.97 (d, 3H). ^13^C NMR (125 MHz, CDCl_3_): δ = 162.5, 160.5, 155.2, 141.9, 138.9, 137.1, 130.1, 130.1, 129.4, 121.9, 120.8, 114.4, 114.0, 113.4, 101.5, 70.7, 57.2, 54.5, 33.8, 30.5, 21.8. HPLC purity: 95.7%.

7-(2-(4-Methylpiperazin-1-yl)ethoxy)-3-(1-phenyl-1*H*-1,2,3-triazol-4-yl)-2*H*-chromen-2-one **1f**. Light brown oil, 64% yield. ^1^H NMR (500 MHz, CDCl_3_): δ = 8.71 (s, 1H), 8.69 (s, 1H), 7.79 (d, J = 8.2, 1H), 7.50–7.43 (m, 4H), 7.37 (t, 1H), 6.83 (d, J = 8.2, 1H), 6.79 (sl, 1H), 4.14 (sl, 2H), 2.34 (t, 4H), 2.29 (t, 4H), 2.21 (s, 3H). ^13^C NMR (125 MHz, CDCl_3_): δ = 168.2, 161.7, 159.7, 141.3, 138.1, 129.6, 129.5, 128.7, 125.5, 120.2, 114.0, 113.4, 112.8, 100.9, 68.8, 60.8, 54.8, 53.7, 45.5. HPLC purity: 96.2%.

3-(1-Phenyl-1*H*-1,2,3-triazol-4-yl)-7-(2-(piperazin-1-yl)ethoxy)-2*H*-chromen-2-one **1g**. Dark brown oil, 60% yield. ^1^H NMR (500 MHz, CDCl_3_): δ = 8.77 (s, 1H), 8.71 (s, 1H), 7.79 (d, J = 7.5, 1H), 7.59–7.51 (m, 4H), 7.43 (t, 1H), 6.91 (d, J = 7.9, 1H), 6.87 (s, 1H), 4.23 (sl, 2H). ^13^C NMR (125 MHz, CDCl_3_): δ = 162.5, 161.6, 155.0, 141.7, 138.4, 130.4, 130.0, 129.3, 126.3, 121.0, 120.7, 114.3, 113.7, 113.6, 101.4, 65.5, 55.5, 56.4, 45.8. HPLC purity: 95.3%.

3-(1-Benzyl-1*H*-1,2,3-triazol-4-yl)-7-(2-(piperidin-1-yl)ethoxy)-2*H*-chromen-2-one **1h**. Brown oil, 40% yield. ^1^H NMR (500 MHz, CDCl_3_): δ = 8.60 (s, 1H), 8.25 (s, 1H), 7.51 (d, J = 8.5, 1H), 7.35 -7.30 (m, 4H), 7.26 (m, 1H), 6.87 (d, J = 8.7, 2 Hz. 1H), 6.82 (s, J = 1.6, 1H), 4.16 (t, 2H), 2.55 (sl, 4H), 2.83 (t, 2H), 1.51 (m, 4H), 1.42 (m, 2H). ^13^C NMR (125 MHz, CDCl_3_): δ = 161.5, 159.6, 154.5, 141.0, 137.6, 134.1, 129.2, 128.7, 128.4, 127.7, 123.1, 114.1, 113.2, 112.7, 100.8, 68.5, 56.9, 54.4, 53.9, 24.8, 23.8. HPLC purity: 96.1%.

3-(1-(2-Methoxybenzyl)-1*H*-1,2,3-triazol-4-yl)-7-(2-(piperidin-1-yl)ethoxy)-2*H*-chromen-2-one **1i**. Light brown solid, 55% yield. mp: 140 -142 °C. ^1^H NMR (500 MHz, CDCl_3_): δ = 8.66 (s, 1H), 8.31 (s, 1H), 7.50 (d, J = 8.6 Hz, 1H), 7.33 (t, J = 7.9 Hz, 1H), 7.25 (t, J = 7.9 Hz, 1H), 6.94 (m, J = 14.5, 2H), 6.90 (dd, J = 8.7, 2.3 Hz, 1H), 6.85 (d, J = 1.9 Hz, 1H), 5.60 (s, 2H), 4.18 (t, J = 5.9 Hz, 2H), 3.89 (s, 3H), 2.82 (t, J = 5.7 Hz, 2H), 2.53 (s, 4H), 1.77–1.54 (m, 4H), 1.46 (s, 1H). ^13^C NMR (125 MHz, CDCl_3_): δ = 162.1, 160.1, 157.4, 155.1, 141.1, 137.6, 130.5, 130.4, 129.4, 123.9, 123.1, 121.1, 115.2, 113.7, 113.2, 111.0, 101.4, 77.2, 66.8, 57.7, 55.7, 55.2, 49.4, 26.0, 24.3. HPLC purity: 96.2%.

3-(1-(3-Methoxybenzyl)-1*H*-1,2,3-triazol-4-yl)-7-(2-(piperidin-1-yl)ethoxy)-2*H*-chromen-2-one **1j**. Yellow solid, 44% yield. mp: 141–143 °C. ^1^H NMR (500 MHz, CDCl_3_): δ = 8.68 (s, 1H), 8.28 (s, 1H), 7.51 (d, J = 8.7 Hz, 1H), 7.29 (t, J = 7.9 Hz, 1H), 6.89 (td, J = 8.3, 2.0 Hz, 3H), 6.84 (d, J = 14.0 Hz, 2H), 5.54 (s, 2H), 4.17 (t, J = 5.9 Hz, 2H), 3.78 (s, 3H), 2.81 (t, J = 5.9 Hz, 2H), 2.51 (s, 4H), 1.61 (dt, J = 11.0, 5.5 Hz, 4H), 1.45 (s, 2H). ^13^C NMR (125 MHz, CDCl_3_): δ = 162.2, 160.2, 160.0, 155.1, 141.6, 137.9, 136.1, 130.4, 129.5, 123.5, 120.4, 114.9, 114.4, 113.7, 113.1, 101.3, 77.2, 66.8, 57.7, 55.4, 55.2, 54.4, 26.0, 24.2. HPLC purity: 98.5%.

3-(1-(4-Methoxybenzyl)-1*H*-1,2,3-triazol-4-yl)-7-(2-(piperidin-1-yl)ethoxy)-2*H*-chromen-2-one **1k**. Yellow solid, 85% yield. mp: 131–133 °C. ^1^H NMR (500 MHz, CDCl_3_): δ = 8.67 (s, 1H), 8.24 (s, 1H), 7.50 (d, J = 8.7 Hz, 1H), 7.27 (d, J = 9.1 Hz, 3H), 6.89 (d, J = 8.5 Hz, 3H), 6.85 (d, J = 1.8 Hz, 1H), 5.50 (s, 2H), 4.17 (t, J = 5.9 Hz, 2H), 3.80 (s, 3H), 2.81 (t, J = 5.9 Hz, 2H), 2.52 (s, 4H), 1.68–1.56 (m, J = 11.0, 5.5 Hz, 4H), 1.45 (sl, 2H). ^13^C NMR (125 MHz, CDCl_3_): δ = 162.2, 160.1, 160.0, 155.0, 141.5, 137.8, 129.8, 129.5, 126.7, 123.3, 114.9, 114.6, 113.7, 113.1, 101.3, 77.2, 66.8, 57.7, 55.5, 55.2, 54.0, 26.0, 24.2. HPLC purity: 96.1%.

3-(1-(4-Fluorbenzyl)-1*H*-1,2,3-triazol-4-yl)-7-(2-(piperidin-1-yl)ethoxy)-2*H*-chromen-2-one **1l**. Yellow solid, 45% yield. mp: 167–169 °C. ^1^H NMR (500 MHz, CDCl_3_): δ = 8.69 (s, 1H), 8.28 (s, 1H), 7.58–7.45 (m, 2H), 7.36–7.28 (m, 2H), 7.07 (t, J = 8.3 Hz, 2H), 6.91 (d, J = 8.3 Hz, 2H), 6.86 (s, 1H), 5.55 (s, 2H), 4.18 (s, 2H), 2.82 (s, 2H), 2.52 (sl, 4H), 1.62 (sl, 4H), 1.45 (sl, 2H). ^13^C NMR (125 MHz, CDCl_3_): δ = 162.3, 160.0, 155.1, 141.7, 138.0, 135.4, 133.4, 130.1, 130.1, 129.5, 123.4, 116.4, 116.2, 114.7, 113.8, 113.1, 101.3, 77.2, 66.8, 57.7, 55.2, 53.7, 26.0, 24.2. HPLC purity: 97.3%.

3-(1-(4-Chlorobenzyl)-1*H*-1,2,3-triazol-4-yl)-7-(2-(piperidin-1-yl)ethoxy)-2*H*-chromen-2-one **1m**. Yellow solid, 94% yield. mp: 143–144 °C. ^1^H NMR (500 MHz, CDCl_3_): δ = 8.69 (s, 1H), 8.31 (s, 1H), 7.51 (d, J = 8.4 Hz, 1H), 7.35–7.28 (m, 3H), 7.18 (d, J = 5.9 Hz, 1H), 6.91 (d, J = 8.0 Hz, 1H), 6.86 (sl, 1H), 5.55 (s, 2H), 4.17 (s, 2H), 2.81 (s, 2H), 2.52 (s, 4H), 1.61 (m, 4H), 1.45 (sl, 2H). ^13^C NMR (125 MHz, CDCl_3_): δ = 162.3, 160.0, 155.1, 141.8, 138.0, 136.6, 130.6, 129.5, 129.2, 128.2, 126.2, 123.5, 114.7, 113.8, 113.1, 101.3, 77.2, 66.8, 57.7, 55.2, 53.7, 25.9, 24.2. HPLC purity: 96.7%.

3-(1-(4-Chlorobenzyl)-1*H*-1,2,3-triazol-4-yl)-7-(2-(piperidin-1-yl)ethoxy)-2*H*-chromen-2-one **1n**. Yellow solid, 87% yield. mp: 157–158 °C. ^1^H NMR (500 MHz, CDCl_3_): δ = 8.68 (s, 1H), 8.29 (s, 1H), 7.51 (d, J = 8.6 Hz, 1H), 7.35 (d, J = 8.3 Hz, 2H), 7.25 (d, J = 8.4 Hz, 2H), 6.91 (dd, J = 8.6, 1.9 Hz, 1H), 6.86 (s, 1H), 5.55 (s, 2H), 4.17 (t, J = 5.9 Hz, 2H), 2.81 (t, J = 5.8 Hz, 2H), 2.51 (s, 4H), 1.64–1.57 (m, 4H), 1.45 (s, 2H). ^13^C NMR (125 MHz, CDCl_3_): δ = 162.6, 160.3, 155.4, 142.0, 138.2, 135.3, 133.5, 129.8, 129.8, 123.7, 115.0, 114.1, 113.4, 101.7, 77.2, 67.2, 58.0, 55.5, 53.9, 26.3, 24.5. HPLC purity: 98.1%.

3-(1-(3-Bromobenzyl)-1*H*-1,2,3-triazol-4-yl)-7-(2-(piperidin-1-yl)ethoxy)-2*H*-chromen-2-one **1o**. Yellow solid, 74% yield. mp: 143–145 °C. ^1^H NMR (500 MHz, CDCl_3_): δ = 8.69 (s, 1H), 8.31 (s, 1H), 7.62–7.41 (m, 3H), 7.25 (d, J = 7.2 Hz, 1H), 6.91 (d, J = 8.4 Hz, 1H), 6.86 (sl, 1H), 5.54 (s, 2H), 4.17 (t, J = 5.6 Hz, 2H), 2.81 (t, J = 5.6 Hz, 3H), 2.51 (sl, 4H), 1.74–1.52 (m, 4H), 1.45 (sl, 2H). ^13^C NMR (125 MHz, CDCl_3_): δ = 162.3, 160.0, 155.1, 141.8, 138.0, 136.9, 132.1, 131.1, 130.8, 129.5, 126.7, 123.5, 123.3, 114.7, 113.8, 113.1, 101.3, 77.2, 66.8, 57.7, 55.2, 53.6, 26.0, 24.2. HPLC purity: 95.1%.

3-(1-(4-Bromobenzyl)-1*H*-1,2,3-triazol-4-yl)-7-(2-(piperidin-1-yl)ethoxy)-2*H*-chromen-2-one **1p**. Yellow solid, 73% yield. mp: 153–154 °C. ^1^H NMR (500 MHz, CDCl_3_): δ = 8.68 (s, 1H), 8.29 (s, 1H), 7.51 (dd, J = 8.5, 2.4 Hz, 3H), 7.19 (d, J = 8.2 Hz, 2H), 6.91 (dd, J = 8.6, 1.7 Hz, 1H), 6.86 (sl, 1H), 5.53 (s, 2H), 4.17 (t, J = 5.9 Hz, 2H), 2.80 (t, J = 5.8 Hz, 2H), 2.51 (sl, 4H), 1.63–1.59 (m, 4H), 1.45 (sl, 2H). ^13^C NMR (125 MHz, CDCl_3_): δ = 162.3, 160.0, 155.1, 141.7, 138.0, 133.7, 132.5, 129.8, 129.5, 123.4, 123.1, 114.7, 113.8, 113.1, 101.3, 77.2, 66.9, 57.7, 55.2, 53.7, 26.0, 24.3. HPLC purity: 97.5%.

3-(1-Benzyl-1*H*-1,2,3-triazol-4-yl)-7-(3-(piperidin-1-yl)propoxy)-2*H*-chromen-2-one **1q**. Yellow solid, 64% yield. mp: 158–160 °C. ^1^H NMR (500 MHz, CDCl_3_): δ = 8.68 (s, 1H), 8.28 (s, 1H), 7.51 (d, J = 8.7 Hz, 1H), 7.39–7.35 (m, 3H), 7.32–7.30 (m, 2H), 6.88 (dd, J = 8.6, 2.2 Hz, 1H), 6.84 (d, J = 2.0 Hz, 1H), 5.58 (s, 2H), 4.10 (t, J = 6.1 Hz, 2H), 2.67–2.61 (m, 6H), 2.15 (sl, 2H), 1.73 (sl, 4H), 1.51 (sl, 2H). ^13^C NMR (125 MHz, CDCl_3_): δ = 162.5, 160.3, 155.3, 141.8, 138.1, 135.0, 129.8, 129.5, 129.2, 128.5, 123.8, 115.1, 113.7, 113.4, 101.5, 77.2, 67.2, 56.0, 54.7, 54.7, 26.2, 25.4, 24.2. HPLC purity: 95.2%.

### 3.3. Biological In Vitro Assays

#### 3.3.1. Anticholinesterase Activity Assays

The anticholinesterase activity was determined using Ellman’s method [23], adapted as follows [22,48]. All solutions were prepared at 20 °C in 0.02 M Tris-HCl buffer (pH 7.5; ionic strength ~0.02 M), with stock solutions of test compounds (**1a**–**q**) prepared in a DMSO:EtOH (7:3 *v*/*v*) mixture (20 mM). Experiments were conducted in triplicate in a flat-bottomed 96-well transparent plate (path length of 0.6 cm) by adding 150 μL of serially diluted inhibitor solutions (eight concentrations, 50–0.00078 μM for AChE and 50–0.11719 μM for BChE, dilution factor = 2), alongside negative controls (buffer only) and vehicle controls consisting of the DMSO:EtOH (7:3 *v*/*v*) mixture at the same final concentration present in the corresponding test wells (≤0.2% *v*/*v* DMSO). Subsequently, 60 μL of 5,5′-dithiobis(2-nitrobenzoic acid) (DTNB, 1.1 mM) and 30 μL of enzyme solution (electric eel AChE [EeAChE], human AChE [hAChE], or equine serum BChE [EqBChE], 0.20 U/mL) containing 1 mg/mL bovine serum albumin (BSA) were added. Initial absorbance was recorded at λ = 415 nm using an iMark microplate reader (Bio-Rad, Hercules, CA, USA) to establish a blank reference. After 10 min incubation at 30 °C, 24 μL of substrate (2.75 mM acetylthiocholine iodide [ACTI] or S-butyrylthiocholine iodide [BCTI]) was added, and absorbance was measured three times at 30 s intervals under the same conditions. Enzyme activity was calculated as the percentage relative to untreated controls, subtracting the blank reference. IC_50_ values were derived via non-linear regression analysis of dose–response curves (Figure S4) using GraphPad Prism v7.0.

#### 3.3.2. Enzymatic Kinetic Study

The enzymatic kinetics of cholinesterase inhibition were determined according to an adapted Ellman’s method [23]. All of the solutions were prepared at 20 °C in 0.02 M Tris-HCl buffer (pH 7.5; ionic strength ~0.02 M), stock solutions of test compounds were prepared in DMSO:EtOH (7:3) mixture (20 mM), and the experiment was conducted in triplicate. To a flat-bottom 96-well transparent plate (path length of 0.6 cm) were added 150 μL of treatment solutions with inhibitors **1a**, **1h,** and **1j** at two different concentrations (Table S1) distributed in eight sets of triplicate each. Eight sets of untreated triplicates were used as a negative control. Following, there were added 60 μL of 5,5′-Dithiobis(2-nitrobenzoic acid) (DTNB, Ellman’s reagent) at 1.1 mM and 30 μL of electric eel acetylcholinesterase (EeAChE) or equine serum butyrylcholinesterase (EqBuChE) at 0.20 U/mL in the presence of 1 mg/mL bovine serum albumin (BSA). Absorbance was then recorded using an iMark plate reader (Bio-Rad) equipped with a λ = 415 nm light filter, and this measure was used as a blank reference. After 10 min incubation at room temperature, 24 μL of acetylthiocholine iodide (ACTI) or butyrylthiocholine iodide (BCTI) at eight concentrations serially diluted (factor = 1.3) from 2.75 to 0.44 mM (final concentration: 0.25–0.04 mM) were added to all wells and the absorbance recorded at room temperature at λ = 415 nm after 2 min of incubation until completing 10 min with an interval between readings of 60 s. The Lineweaver–Burk reciprocal plots were obtained by plotting a 1/velocity versus 1/[substrate] graph for two different inhibitor concentrations and an untreated control. The linear regression of each data set shows convergent behavior, in that the region to which the curves converge determines the type of inhibition observed. Ki, Ki’ (competitive and noncompetitive inhibition constants, respectively), Km (Michaelis-Menten constant), and Vmax values were calculated with Graphpad Prism v7.0 using the non-linear regression models for enzyme kinetics inhibition and enzyme kinetics–substrate vs. velocity.

#### 3.3.3. Evaluation of H_3_R Affinity

HEK-293 cells (human embryonic kidney cells) stably expressing *h*H_3_R were washed and harvested in PBS buffer (phosphate-buffered saline). The cells were then centrifuged (3000× *g*, 10 min, 4 °C) and homogenized using an Ultra-Turrax^®^ homogenizer in ice-cold H_3_R binding buffer (12.5 mM MgCl_2_, 100 mM NaCl, and 75 mM Tris/HCl, pH 7.4). The cell membrane homogenate was centrifuged (20,000× *g*, 20 min, 4 °C), and the resulting pellet was resuspended in binding buffer and stored at −80 °C until use [49].

Prior to experiments, the cell membranes were thawed, homogenized by sonication at 4 °C, and kept in ice-cold binding buffer. Crude membrane extracts (20 μg/well in a final volume of 0.2 mL binding buffer) were incubated with [^3^H]-*N*-α-methylhistamine (2 nM; 78.3 Ci/mmol) and varying concentrations of test compounds. Assays were performed in duplicate, with compound concentrations ranging from 0.01 nM to 100 μM. Incubations proceeded for 90 min at room temperature under continuous agitation. Nonspecific binding was determined in the presence of 10 μM pitolisant.

Bound radioligand was separated from free radioligand by filtration through GF/B filters pretreated with 0.3% (*w*/*v*) polyethyleneimine, using a cell harvester. Radioactivity was quantified by liquid scintillation counting. Data were analyzed using GraphPad Prism v8 (San Diego, CA, USA) software, employing non-linear regression analysis [46].

#### 3.3.4. Monoamine Oxidase A/B Inhibitory Activity

The activity of human recombinant membrane-bound monoamine oxidase isoforms A and B (MAO-A/B) (Sigma-Aldrich, Gillingham, UK) in 50 mM potassium phosphate buffer (pH 7.5) was determined by measuring hydrogen peroxide production coupled to the fluorescent dye Amplex Red (Sigma-Aldrich, UK) at a final concentration of 50 μM, in the presence of horseradish peroxidase (HRP, 2.5 U/mL). This coupling reaction generates the fluorescent product resorufin, which was quantified at 30 °C using a fluorescence plate reader. Under the assay conditions, the K_m_ for tyramine was 0.4 mM for MAO-A and 0.16 mM for MAO-B. The tested compounds did not interfere with resorufin fluorescence or inhibit the coupling enzyme (HRP).

IC_50_ values for MAO-A and MAO-B were determined for each compound (>10 concentrations, in duplicate) at 30 °C. IC_50_ values were calculated from reaction rates measured at varying inhibitor concentrations, using a substrate concentration of 2 × K_m_. Reactions were initiated by adding the substrate and dye after a 30 min preincubation of the inhibitor with the enzyme. Data were analyzed using a three-parameter equation in GraphPad Prism 7.0 (San Diego, USA). Each reported value represents the mean of at least two independent determinations.

### 3.4. Cytotoxicity in SH-SY5Y Neuroblastoma Cell Line

#### 3.4.1. Culture Conditions for the SH-SY5Y Neuroblastoma Cell Line

The immortalized human neuroblastoma cell line (SH-SY5Y–ATCC: CRL-2226; BCRJ:0223) was used to evaluate cytotoxicity. The cell line was cultured in Dulbecco’s Modified Eagle’s Medium and F12 in a 1:1 ratio (DMEM:F12; Sigma-Aldrich code 0697, Brazil) supplemented with 10% fetal bovine serum (FBS; Sigma-Aldrich code F2442, Brazil) and 1% streptomycin/penicillin (10,000 U/mL and 10,000 μg/mL, respectively; Nova Biotecnologia code BR30110-01, Cotia, SP, Brazil) and incubated at 37 °C with 5% CO_2_. The culture medium was replaced every 48 h, until the cells reached semi-confluence (≥70%). Cell passage was carried out with the aid of trypsin-EDTA/0.25% solution (Nova Biotecnologia, Brazil) and divided between 5 or 8 mL culture volumes depending on the respective bottles (25 or 75 cm^2^) used.

#### 3.4.2. Preparation of Samples for the MTT Cytotoxicity Assay

For each sample under study, a stock solution in dimethyl sulfoxide (DMSO) was prepared and diluted using culture medium to concentrations of 12.5, 25 and 50 µM. The sample representing the blank/vehicle was prepared using 20 µL of pure DMSO and 1980 µL of culture medium, representing 1% DMSO. Vehicle and pure DMSO were used as negative and positive controls, respectively.

#### 3.4.3. Cytotoxicity Assay Using the MTT Method

The in vitro cytotoxicity assay evaluated cell viability using the [3-(4,5-dimethylthiazol-2yl)-2,5-diphenyl tetrazolium] bromide (MTT) reduction assay. Cells were seeded at an initial density of 5.0 × 10^4^ cells/well in 96-well plates in a volume of 200 µL. The plates were incubated at 37 °C containing 5% CO_2_ for 24 h. In the next step, the culture medium was replaced by the sample solution previously described (Section 3.4.2). After 24 h exposure, the medium was replaced with 100 μL of MTT in phosphate-buffer saline (PBS), pH 7.4, at a concentration of 0.5 mg.mL^−1^/well and the plates were incubated for an additional 3 h. Then, the MTT solution was removed, and 100 μL of DMSO was added to solubilize the formazan crystals. The cell viability was measured by absorbance at 562 nm using a Microplate Absorbance Reader (Kasuaki, Brazil). All concentrations tested were evaluated in nine replicates (n = 9). The variation in results was observed using ANOVA followed by Tukey’s test for multiple comparisons through GraphPad Prism v5^TM^ with a significance level of *p* < 0.05.

### 3.5. In Vitro Neuroprotection Assay Against SH-SY5Y Neuroblastoma Cell Line

To evaluate the neuroprotective activity of compounds **1h**, **1k**, **1j,** and **1q**, a stock solution at a concentration of 1.25 mM was prepared in DMSO and diluted using culture medium to a concentration of 12.5 µM. Donepezil is an acetylcholinesterase inhibitor and was used as a positive control at a concentration of 10 µM. Hydrogen peroxide solution (H_2_O_2_ 400 µM) was the compound used to induce damage due to oxidative stress in the SH-SY5Y cell line [50,51]. For the neuroprotection assay, the cells previously cultured (Section 3.4.1) were seeded in 96-well plates at a density of 5 × 10^4^ cells/well in a volume of 200 µL. The plates were incubated at 37 °C containing 5% CO_2_ for 24 h. After 24 h, the medium was replaced with the test solutions. Compounds **1h**, **1k**, **1j,** and **1q** at a concentration of 12.5 µM, and the positive control were added and maintained in contact with cells for 2 h. Finally, the test solutions were removed, the cells were washed with PBS pH 7.4, and the H_2_O_2_ solution was added and maintained in contact with the cells for 24 h. After 24 h exposure, the neuroprotective capacity was assessed through cell viability using the MTT assay protocol previously described in Section 3.4.3 [52,53].

### 3.6. Molecular Modeling

A molecular docking study was implemented with the acetylcholinesterase of the electric eel (*Electrophorus electricus*) (EeAChE) and with the horse (*Equus caballus*) butyrylcholinesterase (EqBChE) to obtain insight into possible reasons for the observed enzyme inhibition data at the molecular level.

For EeAChE, the crystallographic structure (PDB code: 1C2B, resolution: 4.5 Å) was retrieved from the Protein Data Bank [54]. For context and comparison, the high-resolution structure of human acetylcholinesterase (hAChE, PDB: 4EY7, resolution: 2.35 Å) was also used for **1j** [55]. For EqBChE, there is no available crystallographic structure, so it was necessary to construct a 3D model from a sequence available in the UniProtKB/Swiss-Prot protein sequence database (entry Q9N1N9), with human BChE (PDB code: 4BDS, resolution: 2.1 Å) [56] as the template. The final model exhibited 89.89% sequence identity and 87% coverage relative to the human enzyme. To validate the docking protocol, redocking experiments were performed using GOLD v5.6 (CCDC), assessing its ability to reproduce reliable binding poses for cholinesterases. The radius of the binding sites for the enzymes was 15 Å around suitable amino acid atoms, selected based on literature information for each binding site, and large enough to cover CAS and PAS (Figure S14). The structural water molecules were removed.

Spartan’14 program (Wavefunction, Inc., Irvine, CA, USA) was utilized to construct and optimize the molecules in study, with the PM6 method.

In the GOLD docking program, the docking functions yield the “fitness scores”, which are dimensionless values. In each case, the score values are a guide to how good the docking pose is, with a higher score indicating a better docking result.

## 4. Conclusions

In this work, we synthesized seventeen novel 7-alkoxyamino-3-(1,2,3-triazolyl)coumarin derivatives via a straightforward Sonogashira cross-coupling reaction applied for the first time at position 3 of the coumarin nucleus, enabling the construction of 1,2,3-triazole moieties. The AChE/BChE inhibition assays revealed this series as potent and highly selective mixed-type AChE inhibitors, with IC_50_ values as low as 4 nM for eeAChE and 13 nm for hAChE, and selectivity ratios of up to 686-fold over BChE, surpassing the profile of reference drug donepezil. SAR analyses highlighted the importance of conformational freedom introduced by the benzylic substituents on the 1,2,3-triazol nucleus for the improvement of AChE modulation. Kinetic studies and molecular docking further indicated that the compounds interact with both the CAS and PAS in a dual-active mode, similar to that of donepezil. Regarding their multi-target profile, it can be inferred that variations in the substitution pattern may result in distinct activity profiles. Changes in the length of the alkoxy-piperidinyl chain or in the conformational flexibility of the 1,2,3-triazole moiety can shift the primary activity toward either AChE inhibition or H_3_R modulation, depending on the specific substitution pattern.

Finally, the activity profile for compound **1h** (AChE IC_50_ = 6.03 nM, H_3_R K_i_ = 558 nM), compound **1q** (H_3_R K_i_ = 151 nM, AChE IC_50_ = 1950 nM, and MAO-B IC_50_ = 1688 nM), and compound **1b** (H_3_R K_i_ = 32 nM, AChE IC_50_ = 1330 nM), summed to the complete absence of neurotoxicity up to 25 µM, and the ability of neuroprotection for some compounds, suggests that this series can represent a valuable model for the development of new candidates of MTDLs for the treatment of Alzheimer’s disease. To achieve this objective, our forthcoming studies’ goals include experimental ADME profiling to prioritize compounds based on kinetic solubility, metabolic stability, and CYP inhibition for in vivo assays.

## Figures and Tables

**Table 1:** Results of half maximum inhibitory concentration obtained for compounds **1a**–**q** against *Ee*AChE and *Eq*BChE.

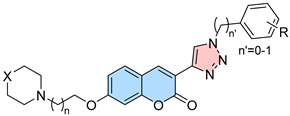							
					IC_50_(nM) ± SD ^a^		
Compound	n	X	n’	R	AChE ^b,c^	BChE ^d^	SI ^e^
**1a**	1	-CH_2_	0	Ph	50.02 ± 2.04 ^b^	8660 ± 150	173
**1b**	2	-CH_2_	0	Ph	1330 ± 45 ^b^	3970 ± 94	3
**1c**	3	-CH_2_	0	Ph	1110 ± 10 ^b^	2410 ± 29	~2
**1d**	4	-CH_2_	0	Ph	4790 ± 290 ^b^	3540 ± 81	~1
**1e**	1	-CHCH_3_	0	Ph	1690 ± 75 ^b^	11,130 ± 800	~7
**1f**	1	-NCH_3_	0	Ph	3160 ± 76 ^b^	8140 ± 890	3
**1g**	1	-NH	0	Ph	1060 ± 74 ^b^	9840 ± 130	~8
**1h**	1	-CH_2_	1	Ph	6.03 ± 0.62 ^b^/ 115.0 ± 8.1 ^c^	3790 ± 400	632/33 ^f^
**1i**	1	-CH_2_	1	2-OCH_3_-Ph	15.26 ± 0.25 ^b^	2810 ± 230	184
**1j**	1	-CH_2_	1	3-OCH_3_-Ph	4.16 ± 0.11 ^b^/ 12.51 ± 0.55 ^c^	2880 ± 120	686/222 ^f^
**1k**	1	-CH_2_	1	4-OCH_3_-Ph	4.58 ± 0.62 ^b^	2080 ± 99	443
**1l**	1	-CH_2_	1	4-F-Ph	23.41 ± 1.77 ^b^	4120 ± 110	176
**1m**	1	-CH_2_	1	3-Cl-Ph	13.08 ± 0.83 ^b^	1950 ± 120	149
**1n**	1	-CH_2_	1	4-Cl-Ph	103.8 ± 5.4 ^b^	2240 ± 60	16
**1o**	1	-CH_2_	1	3-Br-Ph	7.92 ± 0.48 ^b^	2090 ± 54	265
**1p**	1	-CH_2_	1	4-Br-Ph	12.88 ± 0.96	1140 ± 70	88
**1q**	2	-CH_2_	1	Ph	1950 ± 86 ^b^/ 1412 ± 64 ^c^	3560 ± 190	~2 ^e,f^
**Donepezil**	-	-	-	-	7.02 ± 0.24 ^b^/ 89 ^g^	2390 ± 110	341/27 ^f^

^a^ Concentration required to inhibit 50% of cholinesterase activity, data obtained ± standard deviation (SD) from triplicates of three independent experiments; ^b^ Electric eel AChE; ^c^ Human AChE; ^d^ equine serum BChE; ^e^ Selectivity Index (SI) is given as *Eq*BChE IC_50_/*Ee*AChE IC_50_, ^f^ SI is given as *Eq*BChE IC_50_/*h*AChE, ^g^ Reference [24].

**Table 2:** Kinetic parameters of **1a** and **1h** in *Ee*AChE and *Eq*BChE.

Conc. (μM)	V_max_ ± SD (1 × 10^3^ nM/min)	K_m_ ± SD (1 × 10^3^ nM)	K_i_ (nM) ± SE ^a^	K_i’_ (nM) ± SE ^b^
**1a** in AChE				
0	6.10 ± 0.09	40.38 ± 1.57		
0.02	2.78 ± 0.04	70.30 ± 1.53	30.79 ± 5.38	61.59 ± 20.04
0.06	2.06 ± 0.03	102.1 ± 0.10		
**1a** in BChE				
0	13.92 ± 0.09	85.46 ± 0.83		
4	11.69 ± 0.20	113.1 ± 0.8	3940 ± 230	11,400 ± 1100
8	8.24 ± 0.21	157.5 ± 1.5		
**1h** in AChE				
0	8.50 ± 0.17	72.19 ± 6.82		
0.005	4.45 ± 0.02	88.07 ± 6.78	4.73 ± 0.88	7.17 ± 1.48
0.007	3.19 ± 0.010	93.73 ± 7.01		
**1h** in BChE				
0	4.51 ± 0.16	88.7 2± 7.61		
2.8	3.62 ± 0.14	234.4 ± 16.2	1250 ± 150	4830 ± 620
4.8	3.40 ± 0.03	355.7 ± 19.2		

Data obtained ± standard deviation (SD) or ± standard error (SE) from triplicates of independent experiments. ^a^ Affinity constant of inhibition of free enzyme; ^b^ Affinity constant of inhibition of enzyme-substrate complex. Values were obtained via global non-linear regression to the mixed-inhibition rate equation.

**Table 4:** H_3_R affinity (*K_i_*) and MAO-A/B inhibitory activity of compounds **1b**, **1h** and **1q**.

Compound	*K_i_* hH_3_R [nM] ^a^	*h*MAO-A IC_50_ [nM] ^a^	*h*MAO-B IC_50_ [nM] ^a^
**1b**	32 [25; 41]	6.4 ± 0.5 ^b^	11.4 ± 1.9 ^b^
**1h**	558 [337; 925]	5.2± 1.9 ^b^	11.3 ± 1.9 ^b^
**1q**	151 [71; 325]	31,055 [11,758; 82,018]	1688 [1164; 2449]
**Pitolisant**	6.5 [2.3; 18]	-	-
**Rasagiline**	-	-	25 ± 7 ^c^

^a^ The 95% confidence interval is given in brackets; ^b^ Percentage of MAO A/B inhibition at a test concentration of 1 µM ± SEM; ^c^ IC_50_ ± SD.

## Data Availability

Data is contained within the article and Supplementary Material.

## Supplementary Materials

The following supporting information can be downloaded at: https://www.mdpi.com/article/10.3390/ph18091398/s1.
