# Supplementary material for: Design, Synthesis, and Biological Evaluation of Novel Multitarget 7-Alcoxyamino-3-(1,2,3-triazole)-coumarins as Potent Acetylcholinesterase Inhibitors
Source: Pharmaceuticals (Basel). 2025 Sep 17;18(9):1398. doi: 10.3390/ph18091398 (PMC12472947; doi:10.3390/ph18091398)
Supplement: Supplementary file 1 [file pharmaceuticals-18-01398-s001.zip › pharmaceuticals-3844212-supplementary.pdf]

Design, Synthesis, and Biological Evaluation of Novel Multitarget 7-alcoxyamino-3-(1,2,3-triazole)-coumarins as Potent Acetylcholinesterase Inhibitors

Nathalia F. Nadur[1], Larissa A. P. Ferreira[1], Daiana P. Franco[1], Luciana L. Azevedo[1], Lucas Caruso[1], Thiago da S. Honório[2], Priscila de S. Furtado[2], Alice Simon[2], Lucio M. Cabral[2], Tobias Werner[3], Holger Stark[3] and Arthur E. Kümmerle[1*]

Laboratory of Molecular Diversity and Medicinal Chemistry (LaDMol-QM), Graduate Program in Chemistry (PPGQ), Institute of Chemistry, Federal Rural University of Rio de Janeiro, 23897-000 - Seropédica - RJ, Brazil.

2 Cell Culture Laboratory (LabCel), Department of Drugs and Pharmaceutics, Faculty of Pharmacy, Universidade Federal do Rio de Janeiro, Rio de Janeiro 21941-902, Brazil.

3 Institute of Pharmaceutical and Medicinal Chemistry, Heinrich Heine University Düsseldorf, Duesseldorf, Germany.

* Correspondence: akummerle@hotmail.com; Tel.: +55-21-998576298.

Supporting Information

Contents

Biological assessment 2

Biological assessment results 2

Molecular Modeling 11

ADMET Evaluations 15

Copies of ^{1}H, ^{13}C NMR 32

HPLC data 67

References 84

Biological assessment

Anticholinesterase activity assays

Table S1. Inhibitor concentrations for the kinetic study on AChE and BChE.

	Inhibitor concentrations (μM)		
**Compound**	**1a**	**1h**	**1j**
**AChE**	0.02 and 0.06	0.005 and 0.007	0.003 and 0.005
**BChE**	4.0 and 8.0	2.8 and 4.8	1.9 and 3.9

Biological assessment results

Enzymatic kinetic study

Table S2. Kinetic parameters of **1a**, **1h** and **1j** in AChE and BChE.

Concentration (μM)	*Vmax* ± SD (1×10^3 nM/min)	Km ± SD (1×10^3 nM)	K_i (nM) ± SD[a]	$K_{i'}$ (nM) ± SD[b]
		**1a in AChE**		
0	6.10 ± 0.088	40.38 ± 1.566		
0.02	2.78 ± 0.044	70.30 ± 1.529	3.15 ± 0.143	14.27 ± 0.592
0.06	2.06 ± 0.031	102.10 ± 0.115		
		**1a in BChE**		
0	13.92 ± 0.093	85.46 ± 0.826		
4	11.69 ± 0.201	113.07 ± 0.833	3502 ± 64.9	9174 ± 163
8	8.24 ± 0.212	157.53 ± 1.485		
		**1h in AChE**		
0	8.50 ± 0.1743	92.19 ± 6.8207		
0.005	4.45 ± 0.02231	92.78 ± 9.2653	1.682 ± 0.038	4.780 ± 0.0106
0.007	3.19 ± 0.0981	93.73 ± 7.0155		
		**1h in BChE**		
0	4.51 ± 0.1648	88.72 ± 7.6123		
2.8	3.62 ± 0.1358	234.43 ± 16.1506	980 ± 24	2670 ± 67
4.8	3.40 ± 0.0349	354.67 ± 19.2448		
		**1j in AChE**		
0	9.53 ± 0.0584	64.30 ± 0.6389	0.508 ± 0.0001	

0.003	5.21 ± 0.0656	156.63 ± 4.4377		1.696 ±
0.005	3.90 ± 0.1517	208.97 ± 10.6829		0.0038
		**1j in BChE**		
0	9.35 ± 0.1171	96.81 ± 1.6613		
1.9	5.97 ± 0.1562	181.63 ± 6.1890	615 ± 0.58	1619 ± 14
3.9	4.12 ± 0.2257	233.03 ± 16.4394		

[a]Affinity constant for competitive inhibition; [b]Affinity constant for non-competitive inhibition; Data obtained ± standard deviation (SD) from triplicates of independent assays.

Table S3. Global Non-Linear Regression Parameters for Mixed Inhibition.

Compound	Enzyme	α ± SE (95% CI)	Ki ± SE (95% CI) (μM)	Ki′ ± SE (95% CI) (μM)
**1a**	AChE	2.001 ± 0.6489 (0.9906 to 4.110)	0.03079 ± 0.005375 (0.02181 to 0.04678)	0.06159 ± 0.02004 (0.02160 to 0.1923)
**1a**	BChE	2.883 ± 0.3970 (2.183 to 3.876)	3.941 ± 0.2271 (3.507 to 4.455)	11.36 ± 1.097 (7.655 to 17.28)
**1h**	AChE	1.516 ± 0.3136 (0.5256 to 1.900)	0.004727 ± 0.0008777 (0.003344 to 0.007268)	0.007166 ± 0.001482 (0.001757 to 0.01381)
**1h**	BChE	3.852 ± 0.4265 (3.1296 to 4.7514)	1.253 ± 0.1512 (0.9835 to 1.5815)	4.826 ± 0.6248 (3.7574 to 6.1526)
**1j**	AChE	4.248 ± 1.415 (2.164 to 10.18)	0,000637±0,000033 (0.000611 to 0.000657)[a]	0,000270 ± 0,000041 (0.00260 to 0.00280)[a]
**1j**	BChE	3.349 ± 1.984 (2.523 to 4.487)	0.9438 ± 0.1996 (0.6172 to 1.568)	3.1607 ± 1.987 (2.326 to 4.283)

Values represent the best-fit parameters ± standard error (SE) with 95% confidence intervals (95% CI) derived from profile likelihood. The inhibition constant Ki represents affinity for the free enzyme, while Ki′ represents affinity for the enzyme-substrate complex. [a] Value corrected for tight-binding using the Morrison equation.

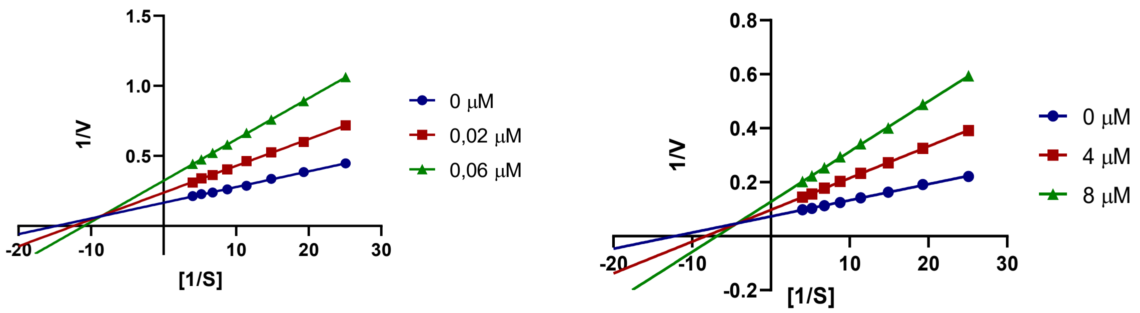

Figure S1. Lineweaver-Burk plots of the prevention kinetics in *Ee*AChE (**left**) and *Eq*BChE (**right**) of the compound **1a**.

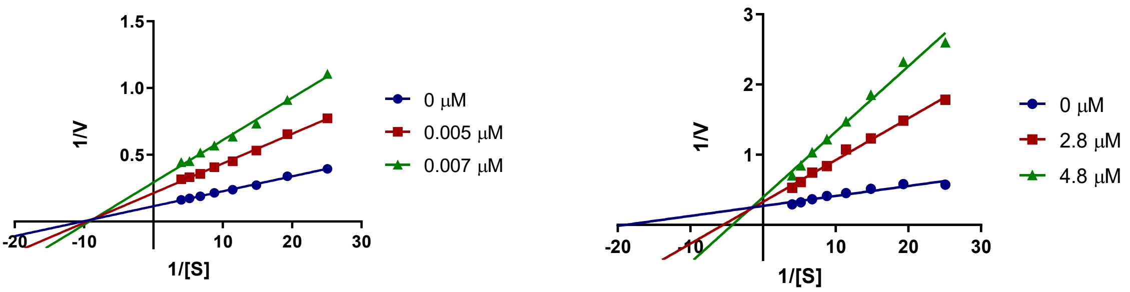

Figure S2. Lineweaver-Burk plots of the prevention kinetics in *Ee*AChE (**left**) and *Eq*BChE (**right**) of the compound **1h.**

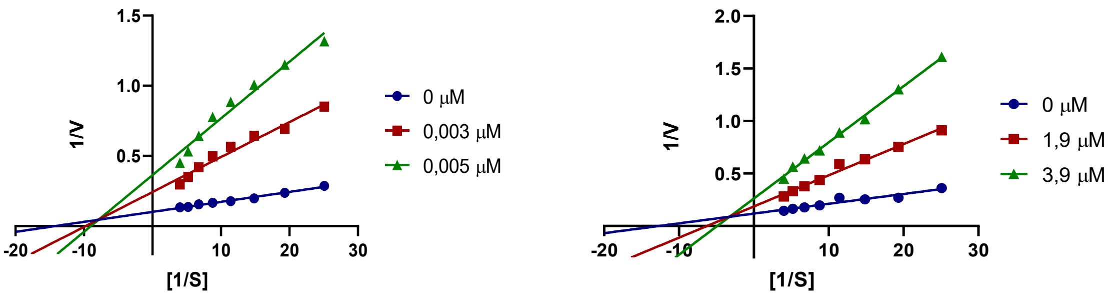

Figure S3. Lineweaver-Burk plots of the prevention kinetics in *Ee*AChE (**left**) and *Eq*BChE (**right**) of the compound **1j.**

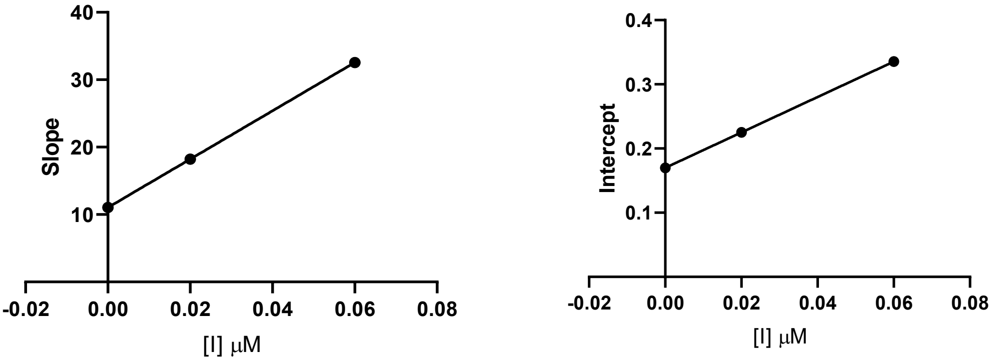

Figure S4. Secondary plots of slope (**left**) and intercept (**right**) versus inhibitor concentration [I] for compound **1a** in *Ee*AChE.

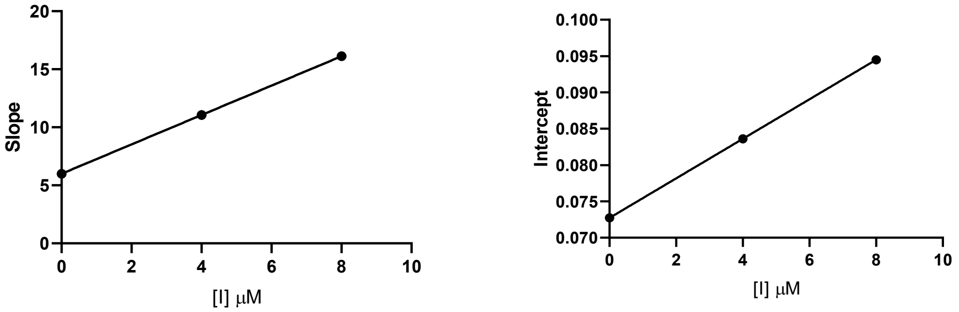

Figure S5. Secondary plots of slope (**left**) and intercept (**right**) versus inhibitor concentration [I] for compound **1a** in *Eq*BChE.

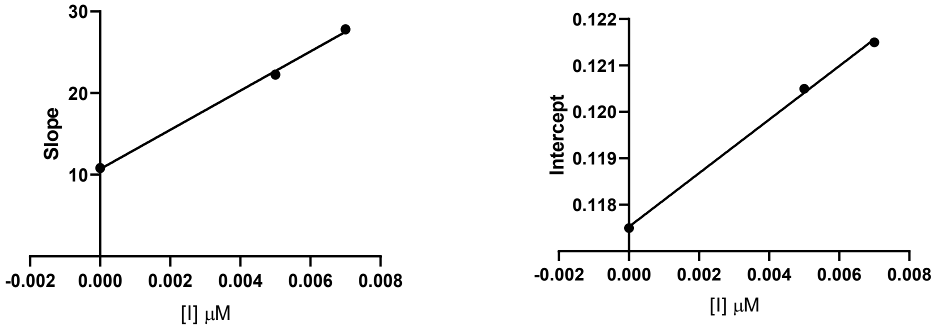

Figure S6. Secondary plots of slope (**left**) and intercept (**right**) versus inhibitor concentration [I] for compound **1h** in *Ee*AChE.

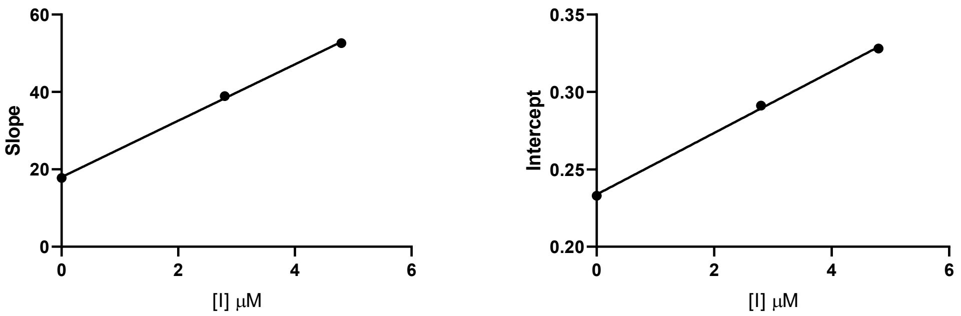

Figure S7. Secondary plots of slope (left) and intercept (right) versus inhibitor concentration [I] for compound **1h** in *Eq*BChE.

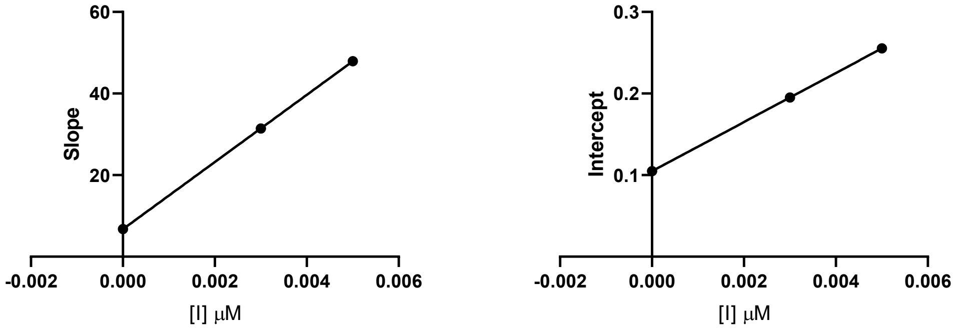

Figure S8. Secondary plots of slope (left) and intercept (right) versus inhibitor concentration [I] for compound **1j** in *Ee*AChE.

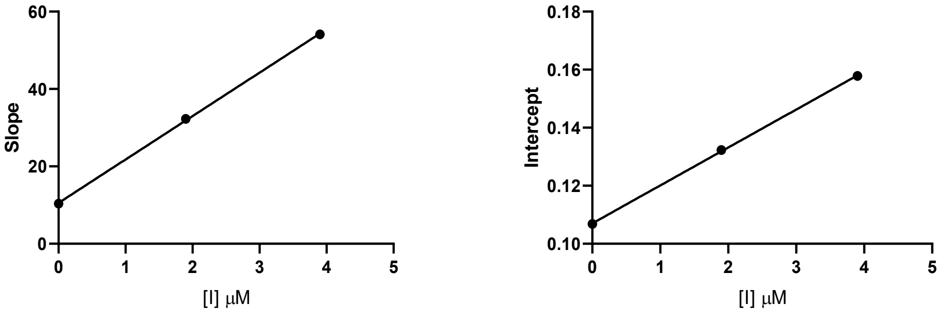

Figure S9. Secondary plots of slope (left) and intercept (right) versus inhibitor concentration [I] for compound **1j** in *Eq*BChE.

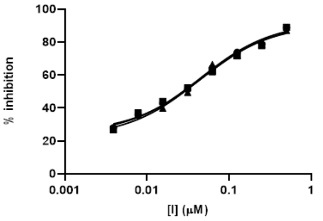

1a

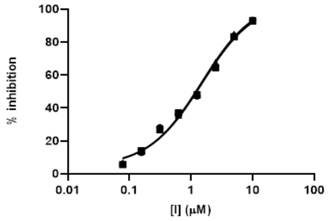

1b

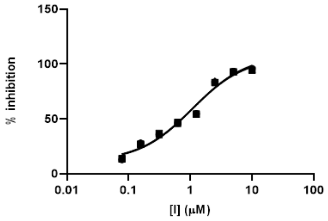

1c

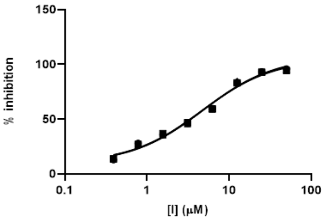

1d

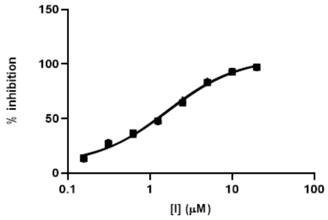

1e

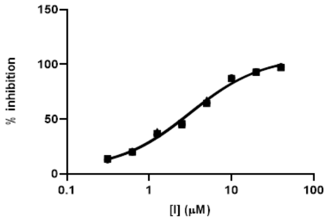

1f

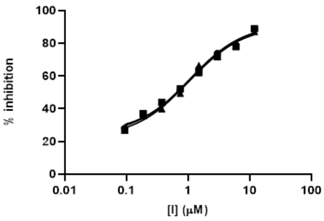

1g

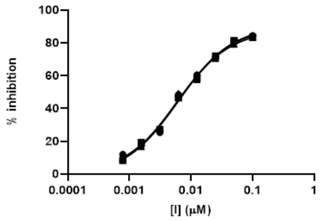

1h

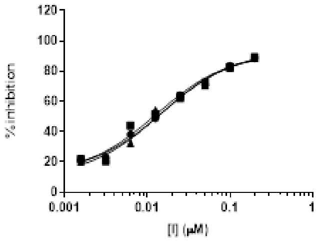

1i

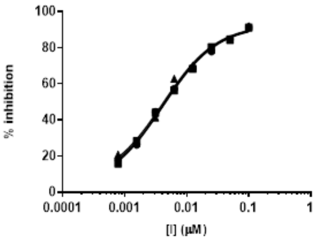

1j

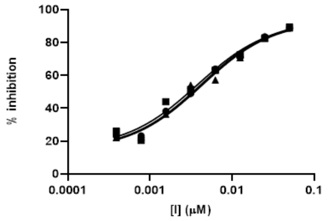

1k

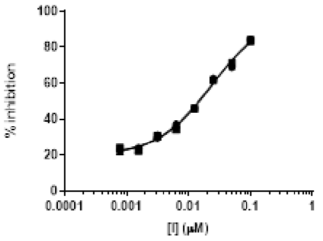

1l

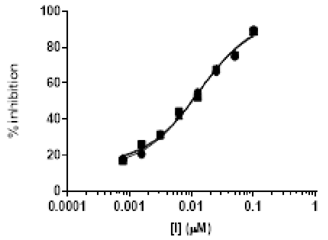

1m

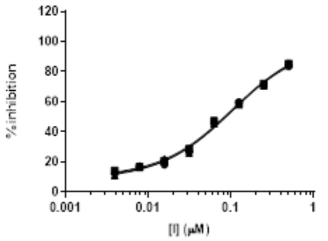

1n

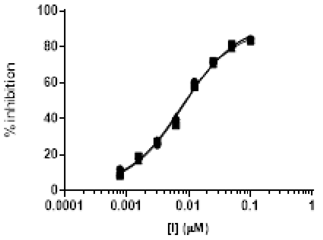

1o

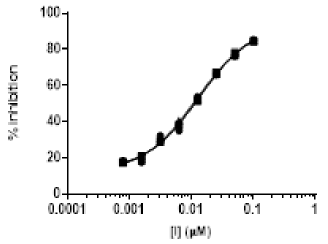

1p

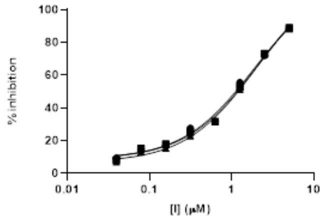

1q

Figure S10. Graphs of *ee*AChE inhibition percentage vs. inhibitor concentration.

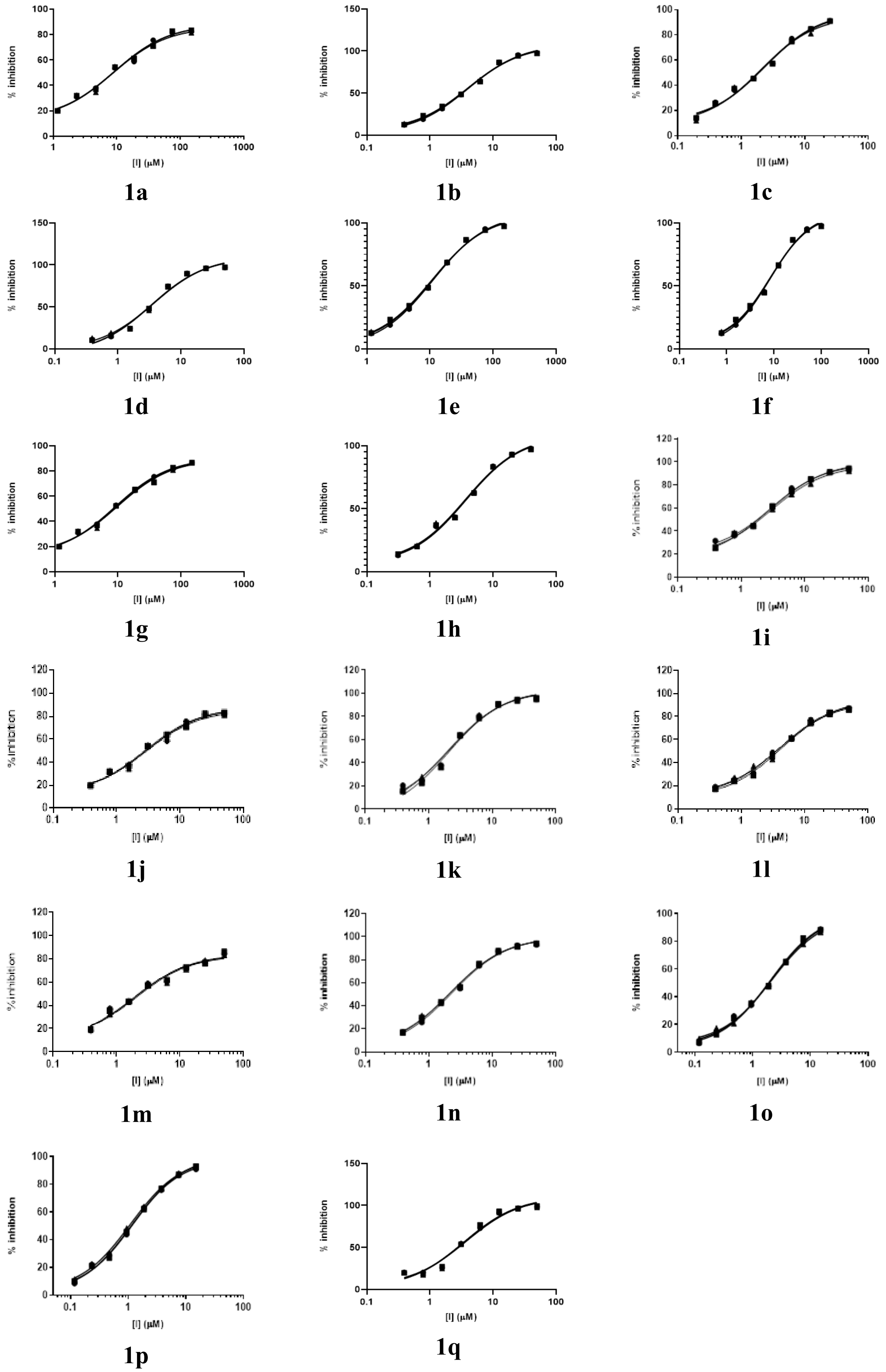

Figure S11. Graphs of *eq*BChE inhibition percentage vs. inhibitor concentration.

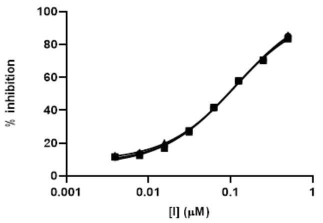

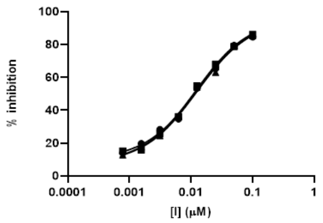

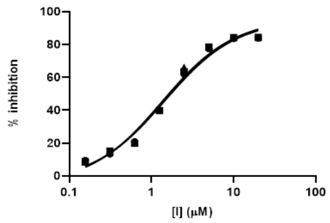

1h **1j** **1q**

Figure S12. Graphs of *h*AChE inhibition percentage vs. inhibitor concentration.

Table S4. Control assays to quantify compound interference on DTNB/thiolate detection at 415 nm.

Control	Absorbance (t = 0 min)	Absorbance (t = 30 min)	ΔAbs[a]	ΔAbs/min	%[b]
**1a**	0.05 ± 0.002	0.051 ± 0.0025	0.001	3.33333E-05	0.06
**1a** + DTNB	0.088 ± 0.0035	0.092 ± 0.0042	0.005	0.000166667	0.31
**1a** + DTNB + ACTI	0.085 ± 0.0032	0.13 ± 0.0123	0.045	0.0015	2.77
**1h**	0.052 ± 0.0015	0.053 ± 0.0017	0.001	3.33333E-05	0.06
**1h** + DTNB	0.088 ± 0.0025	0.093 ± 0.0025	0.005	0.000166667	0.31
**1h** + DTNB + ACTI	0.086 ± 0.0015	0.123 ± 0.0021	0.038	0.001266667	2.34
**Active Essay**	0.145 ± 0.0045	1.772 ± 0.003	1.627	0.054233333	

[a] ΔAbs = Abs(final) - Abs(initial); [b] % Interference = (ΔAbs/min (control) / ΔAbs/min(active enzymatic control)) × 100. Values below 5% are considered insignificant, indicating the compound does not appreciably interfere with the assay detection method.

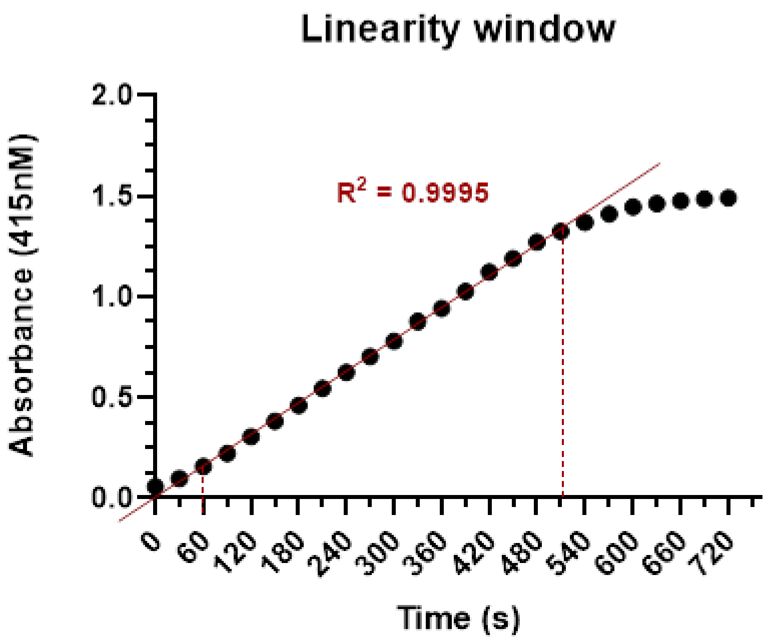

Figure S13. Validation of the Linearity Window for the Enzymatic Assay Using Ellman's Method ($R^2 = 0.9995$).

Table S5. Kinetic parameters of electric eel acetylcholinesterase (*ee*AChE) for the substrate acetylthiocholine.

***Vmax* (µM/min)**	**Km (µM)**
0.171 ± 0.005	97.4 ± 7.1

Data obtained ± standard deviation (SD) from triplicates of independent assays. Initial rates were measured at eight different acetylthiocholine concentrations, ranging from 0 to 800 µM.

Molecular Modeling

Table S6. Docking Score and RMSD Values for the Redocking of *ee*AChE (PDB: 1C2B).

Score Function	DockScore	RMSD (Å)
GoldScore	88.5717	0.6132
CHEMPLP	87.2740	0.8285
ChemScore	61.3541	0.7397
ASP	83.9720	0.7947

Table S7. Docking Score and RMSD Values for the Redocking of *h*AChE (PDB: 4ey7).

Score Function	DockScore	RMSD (Å)
GoldScore	58.4452	1.4686
CHEMPLP	92.7356	0.9999
ChemScore	42.1934	1.5606
ASP	63.9023	1.0263

Table S8. Docking Score and RMSD Values for the Redocking Validation of the *eq*BChE Homology Model.

Score Function	DockScore	RMSD (Å)
GoldScore	33.8079	2.6434
CHEMPLP	51.0350	2.6173
ChemScore	40.2990	3.1314
ASP	35.0566	0.6127

Table S9. Fitness scores obtained by molecular docking in cholinesterase's.

Ligand	*Ee*AChE (Goldscore)	*Eq*BChE (ASP)	*h*AChE (CHEMPLP)
**1a**	80.42	47.95	91.00
**1b**	74.51	48.72	96.40
**1c**	77.83	53.68	-
**1d**	66.75	56.51	-
**1e**	76.62	49.52	-
**1f**	82.46	49.99	-
**1g**	87.90	48.24	-
**1h**	82.01	48.55	96.27
**1i**	83.3	50.06	-
**1j**	73.41	50.67	95.51
**1k**	78.24	50.40	99.48
**1l**	79.61	48.29	-
**1m**	82.87	48.38	-
**1n**	83.49	48.13	-
**1o**	83.98	48.64	-
**1p**	80.18	48.34	-
**1q**	77.19	50.44	96.87
**donepezil**	84.26	45.65	92.74
**tacrine**	-	35.14	-

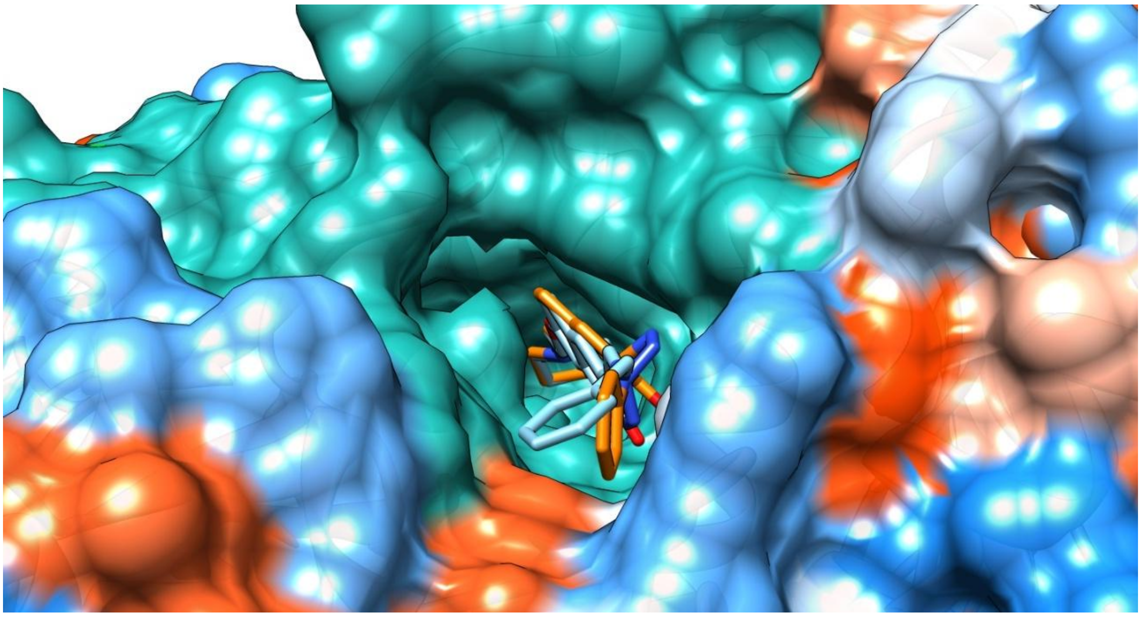

Figure S14. Surface (in green) located within 15 Å of Tyr337, selected for docking studies, illustrating the complete environment surrounding the CAS and PAS sites of Ee AChE (measured by UCSF Chimera, alpha version 1.17).

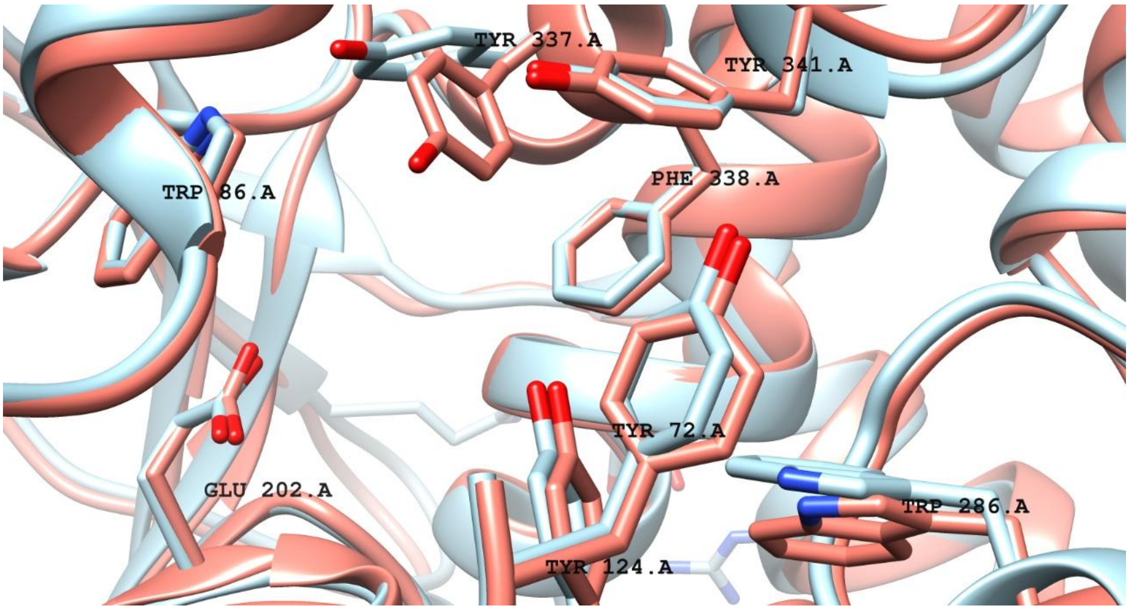

Figure S15. Superposition of eeAChE (1C2B, salmon) and hAChE (4EY7, light blue) showing the main difference between both structures, i.e. the special disposition of Tyr337 and Tyr286.

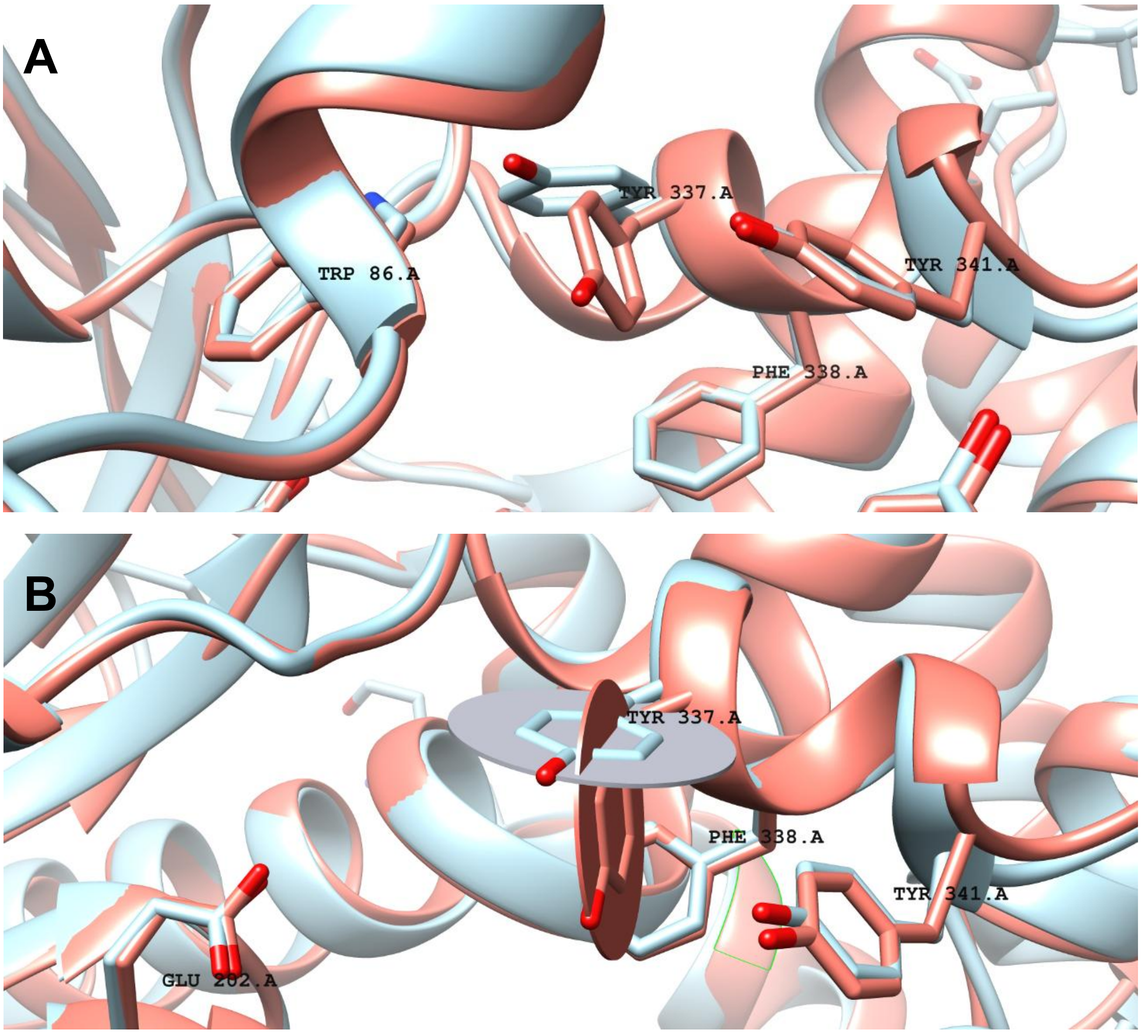

Figure S16. (**A**) Zoom in comparison of Tyr337 of eeAChE (1C2B, salmon) and hAChE (4EY7, light blue). (**B**) Conformational changes represented by Tyr337 ring plans from both cholinesterases: dihedral angle for hAChE of 146.02 º (salmon) and for eeAChE of 85.81 º (light blue), measured by UCSF Chimera, alpha version 1.17.

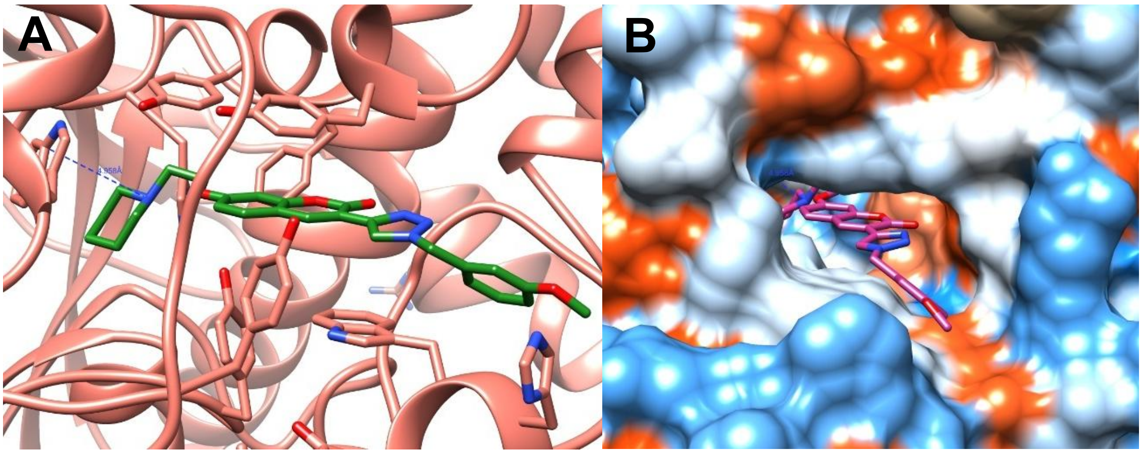

Figure S17. (**A**) Coumarin derivatives **1j** docked within the cavity of the hAChE (4EY7), and (**B**) represented as a Van der Waals surface.

ADMET Evaluations

Table S10. ADMET properties of compound 1a.

Compound 1a

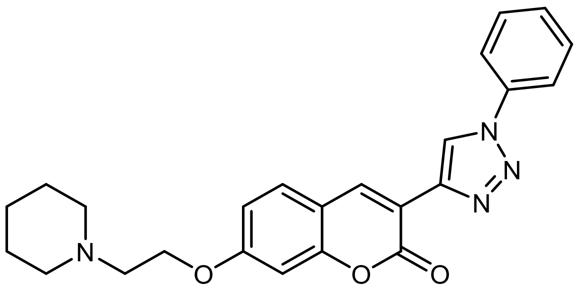

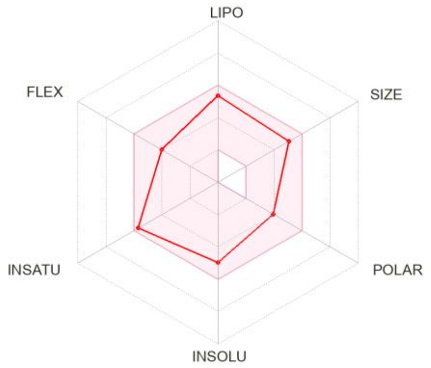

Physicochemical Properties	
Formula	C24H24N4O3
Molecular weight	416.47 g/mol
Fraction Csp3	0.29
Num. rotatable bonds	6
Num. H-bond acceptors	6
Num. H-bond donors	0
Molar Refractivity	122.87
TPSA	73.39 Å²
Lipophilicity	
Log $P_{o/w}$ (iLOGP)	4.09
Log $P_{o/w}$ (XLOGP3)	3.86
Log $P_{o/w}$ (WLOGP)	3.52
Log $P_{o/w}$ (MLOGP)	2.95
Log $P_{o/w}$ (SILICOS-IT)	3.68
Consensus Log $P_{o/w}$	3.62
Water Solubility	
Log S (ESOL)	-4.96
Solubility	4.57e-03 mg/ml; 1.10e-05 mol/l
Class	Moderately soluble
Pharmacokinetics	
GI absorption	High
BBB permeant	Yes
P-gp substrate	Yes
CYP1A2 inhibitor	Yes
CYP2C19 inhibitor	Yes
CYP2C9 inhibitor	Yes
CYP2D6 inhibitor	Yes
CYP3A4 inhibitor	Yes
Log K_p (skin permeation)	-6.23 cm/s
Druglikeness	
Lipinski	Yes; 0 violation
Ghose	Yes
Veber	Yes
Egan	Yes
Muegge	Yes
Bioavailability Score	0.55

Table S11. ADMET properties of compound **1b.**

**Compound 1b**	

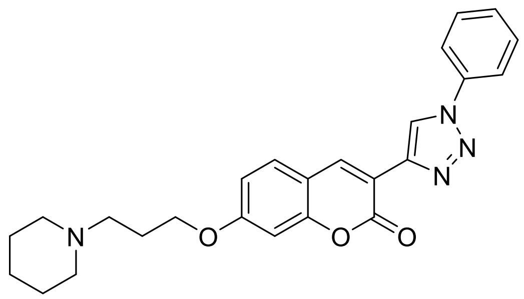

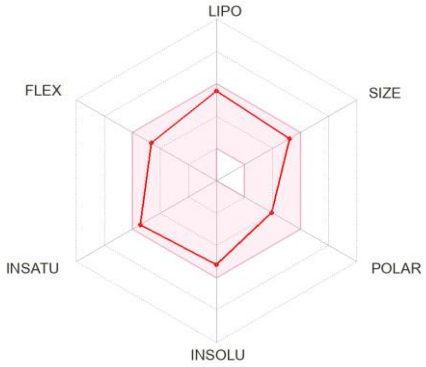

Physicochemical Properties	
Formula	$C_{25}H_{26}N_4O_3$
Molecular weight	430.50g/mol
Fraction Csp3	0.32
Num. rotatable bonds	7
Num. H-bond acceptors	6
Num. H-bond donors	0
Molar Refractivity	127.67
TPSA	73.39 Å^2
Lipophilicity	
Log $P_{o/w}$ (iLOGP)	4.16
Log $P_{o/w}$ (XLOGP3)	4.22
Log $P_{o/w}$ (WLOGP)	3.91
Log $P_{o/w}$ (MLOGP)	3.16
Log $P_{o/w}$ (SILICOS-IT)	4.07
Consensus Log $P_{o/w}$	3.91
Water Solubility	
Log S (ESOL)	-5.19
Solubility	2.77e-03 mg/ml; 6.44e-06 mol/l
Class	Moderately soluble
Pharmacokinetics	
GI absorption	High
BBB permeant	Yes
P-gp substrate	Yes
CYP1A2 inhibitor	Yes
CYP2C19 inhibitor	Yes
CYP2C9 inhibitor	Yes
CYP2D6 inhibitor	Yes
CYP3A4 inhibitor	Yes
Log K_p (skin permeation)	-5.93 cm/s
Druglikeness	
Lipinski	Yes; 0 violation
Ghose	Yes
Veber	Yes
Egan	Yes
Muegge	Yes
Bioavailability Score	0.55

Table S12. ADMET properties of compound **1c.**

**Compound 1c**	

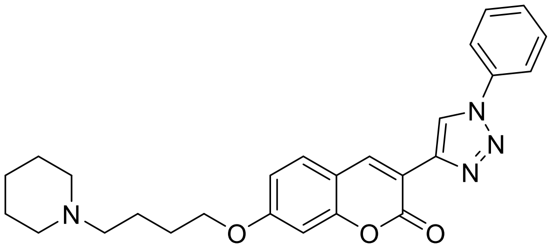

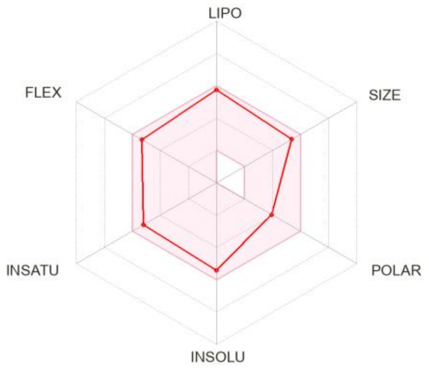

Physicochemical Properties	
Formula	C26H28N4O3
Molecular weight	444.53 g/mol
Fraction Csp3	0.35
Num. rotatable bonds	8
Num. H-bond acceptors	6
Num. H-bond donors	0
Molar Refractivity	132.48
TPSA	73.39 Å^2
Lipophilicity	
Log $P_{o/w}$ (iLOGP)	4.49
Log $P_{o/w}$ (XLOGP3)	4.57
Log $P_{o/w}$ (WLOGP)	4.30
Log $P_{o/w}$ (MLOGP)	3.36
Log $P_{o/w}$ (SILICOS-IT)	4.47
Consensus Log $P_{o/w}$	4.24
Water Solubility	
Log S (ESOL)	-5.42
Solubility	1.70e-03 mg/ml; 3.82e-06 mol/l
Class	Moderately soluble
Pharmacokinetics	
GI absorption	High
BBB permeant	Yes
P-gp substrate	Yes
CYP1A2 inhibitor	Yes
CYP2C19 inhibitor	Yes
CYP2C9 inhibitor	Yes
CYP2D6 inhibitor	Yes
CYP3A4 inhibitor	Yes
Log K_p (skin permeation)	-5.77 cm/s
Druglikeness	
Lipinski	Yes; 0 violation
Ghose	No; 1 violation: MR>130
Veber	Yes
Egan	Yes
Muegge	Yes
Bioavailability Score	0.55

Table S13. ADMET properties of compound **1d.**

Compound 1d

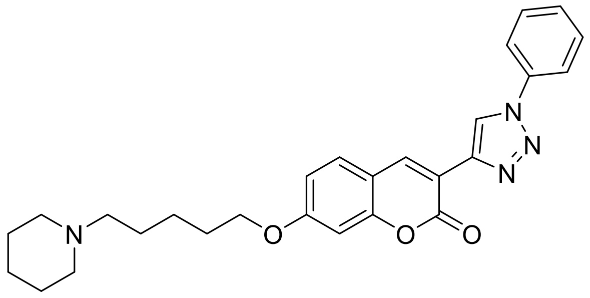

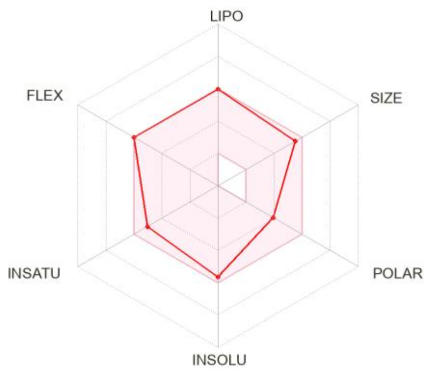

Physicochemical Properties	
Formula	C24H24N4O3
Molecular weight	4.58.55 g/mol
Fraction Csp3	0.37
Num. rotatable bonds	9
Num. H-bond acceptors	6
Num. H-bond donors	0
Molar Refractivity	137.29
TPSA	73.39 Å²
Lipophilicity	
Log $P_{o/w}$ (iLOGP)	4.95
Log $P_{o/w}$ (XLOGP3)	4.93
Log $P_{o/w}$ (WLOGP)	4.69
Log $P_{o/w}$ (MLOGP)	3.56
Log $P_{o/w}$ (SILICOS-IT)	4.86
Consensus Log $P_{o/w}$	4.60
Water Solubility	
Log *S* (ESOL)	-5.65
Solubility	1.02e-03 mg/ml; 2.23e-06 mol/l
Class	Moderately soluble
Pharmacokinetics	
GI absorption	High
BBB permeant	No
P-gp substrate	Yes
CYP1A2 inhibitor	Yes
CYP2C19 inhibitor	Yes
CYP2C9 inhibitor	Yes
CYP2D6 inhibitor	Yes
CYP3A4 inhibitor	Yes
Log K_p (skin permeation)	-6.23 cm/s
Druglikeness	
Lipinski	Yes; 0 violation
Ghose	No; 1 violation: MR>130
Veber	Yes
Egan	Yes
Muegge	Yes
Bioavailability Score	0.55

Table S14. ADMET properties of compound **1e.**

**Compound 1e**	

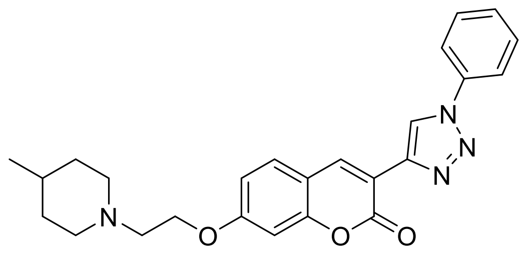

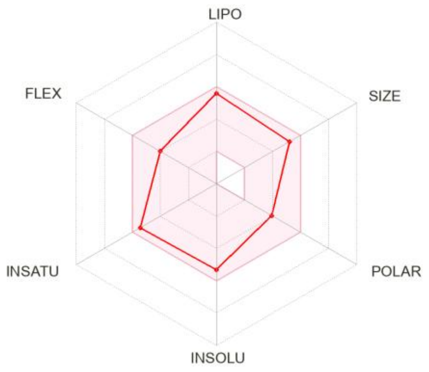

Physicochemical Properties	
Formula	C25H26N4O3
Molecular weight	430.50 g/mol
Fraction Csp3	0.32
Num. rotatable bonds	6
Num. H-bond acceptors	6
Num. H-bond donors	0
Molar Refractivity	127.67
TPSA	73.39 Å²
Lipophilicity	
Log $P_{o/w}$ (iLOGP)	4.35
Log $P_{o/w}$ (XLOGP3)	4.30
Log $P_{o/w}$ (WLOGP)	3.77
Log $P_{o/w}$ (MLOGP)	3.16
Log $P_{o/w}$ (SILICOS-IT)	3.79
Consensus Log $P_{o/w}$	3.87
Water Solubility	
Log S (ESOL)	-5.31
Solubility	2.12e-03 mg/ml; 4.92e-06 mol/l
Class	Moderately soluble
Pharmacokinetics	
GI absorption	High
BBB permeant	Yes
P-gp substrate	Yes
CYP1A2 inhibitor	Yes
CYP2C19 inhibitor	Yes
CYP2C9 inhibitor	Yes
CYP2D6 inhibitor	Yes
CYP3A4 inhibitor	Yes
Log K_p (skin permeation)	-5.87 cm/s
Druglikeness	
Lipinski	Yes; 0 violation
Ghose	Yes
Veber	Yes
Egan	Yes
Muegge	Yes
Bioavailability Score	0.55

Table S15. ADMET properties of compound **1f.**

Compound 1f

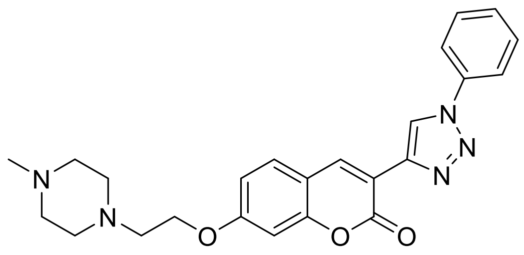

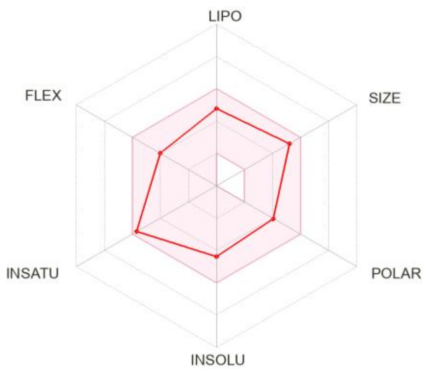

**Physicochemical Properties**	
Formula	C24H25N5O3
Molecular weight	431.49 g/mol
Fraction Csp3	0.29
Num. rotatable bonds	6
Num. H-bond acceptors	7
Num. H-bond donors	0
Molar Refractivity	129.68
TPSA	76.63 Å²
**Lipophilicity**	
Log $P_{o/w}$ (iLOGP)	3.94
Log $P_{o/w}$ (XLOGP3)	2.83
Log $P_{o/w}$ (WLOGP)	1.91
Log $P_{o/w}$ (MLOGP)	2.16
Log $P_{o/w}$ (SILICOS-IT)	2.67
Consensus Log $P_{o/w}$	2.70
**Water Solubility**	
Log S (ESOL)	-4.39
Solubility	1.77e-02 mg/ml; 4.10e-05 mol/l
Class	Moderately soluble
**Pharmacokinetics**	
GI absorption	High
BBB permeant	No
P-gp substrate	Yes
CYP1A2 inhibitor	Yes
CYP2C19 inhibitor	Yes
CYP2C9 inhibitor	Yes
CYP2D6 inhibitor	Yes
CYP3A4 inhibitor	Yes
Log K_p (skin permeation)	-6.92 cm/s
**Druglikeness**	
Lipinski	Yes; 0 violation
Ghose	Yes
Veber	Yes
Egan	Yes
Muegge	Yes
Bioavailability Score	0.55

Table S16. ADMET properties of compound **1g.**

Compound 1g

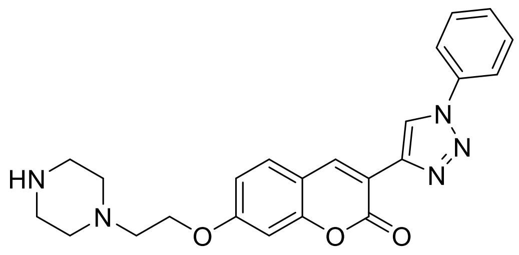

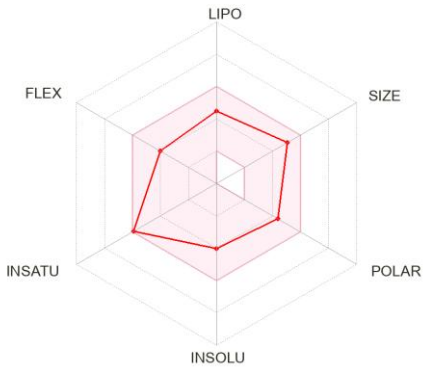

**Physicochemical Properties**	
Formula	C23H23N5O3
Molecular weight	417.46 g/mol
Fraction Csp3	0.26
Num. rotatable bonds	6
Num. H-bond acceptors	7
Num. H-bond donors	1
Molar Refractivity	124.78
TPSA	85.42 Å²
**Lipophilicity**	
Log $P_{o/w}$ (iLOGP)	3.89
Log $P_{o/w}$ (XLOGP3)	2.36
Log $P_{o/w}$ (WLOGP)	1.56
Log $P_{o/w}$ (MLOGP)	1.95
Log $P_{o/w}$ (SILICOS-IT)	2.72
Consensus Log $P_{o/w}$	2.50
**Water Solubility**	
Log *S* (ESOL)	-4.02
Solubility	3.98e-02 mg/ml; 9.54e-05 mol/l
Class	Moderately soluble
**Pharmacokinetics**	
GI absorption	High
BBB permeant	No
P-gp substrate	Yes
CYP1A2 inhibitor	Yes
CYP2C19 inhibitor	No
CYP2C9 inhibitor	Yes
CYP2D6 inhibitor	Yes
CYP3A4 inhibitor	Yes
Log K_p (skin permeation)	-7.17 cm/s
**Druglikeness**	
Lipinski	Yes; 0 violation
Ghose	Yes
Veber	Yes
Egan	Yes
Muegge	Yes
Bioavailability Score	0.55

Table S17. ADMET properties of compound **1h.**

Compound 1h

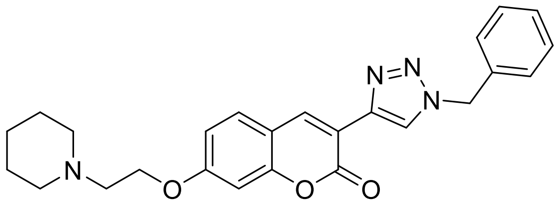

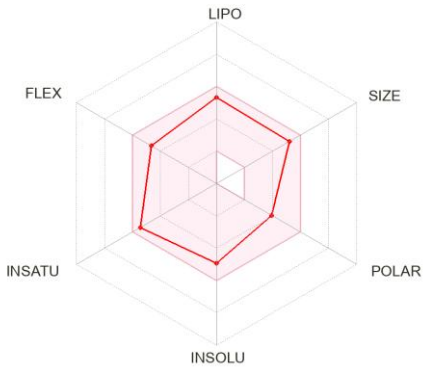

**Physicochemical Properties**	
Formula	C25H26N4O3
Molecular weight	430.50 g/mol
Fraction Csp3	0.32
Num. rotatable bonds	7
Num. H-bond acceptors	6
Num. H-bond donors	0
Molar Refractivity	127.28
TPSA	73.39 Å²
**Lipophilicity**	
Log $P_{o/w}$ (iLOGP)	3.80
Log $P_{o/w}$ (XLOGP3)	3.80
Log $P_{o/w}$ (WLOGP)	3.58
Log $P_{o/w}$ (MLOGP)	2.89
Log $P_{o/w}$ (SILICOS-IT)	4.07
Consensus Log $P_{o/w}$	3.63
**Water Solubility**	
Log S (ESOL)	-4.93
Solubility	5.10e-03 mg/ml; 1.18e-05 mol/l
Class	Moderately soluble
**Pharmacokinetics**	
GI absorption	High
BBB permeant	Yes
P-gp substrate	Yes
CYP1A2 inhibitor	Yes
CYP2C19 inhibitor	Yes
CYP2C9 inhibitor	Yes
CYP2D6 inhibitor	Yes
CYP3A4 inhibitor	Yes
Log K_p (skin permeation)	-6.23 cm/s
**Druglikeness**	
Lipinski	Yes; 0 violation
Ghose	Yes
Veber	Yes
Egan	Yes
Muegge	Yes
Bioavailability Score	0.55

Table S18. ADMET properties of compound **1i.**

Compound 1i

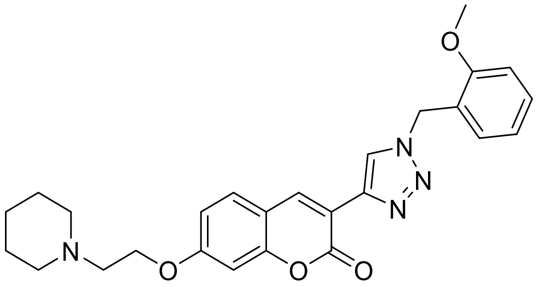

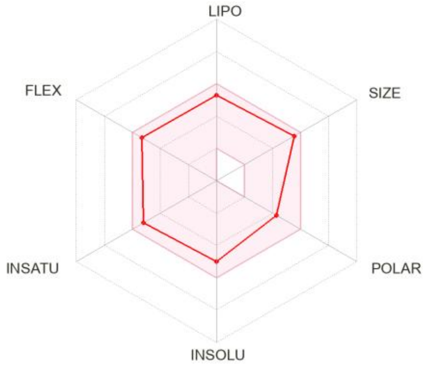

Physicochemical Properties	
Formula	C26H28N4O4
Molecular weight	460.52 g/mol
Fraction Csp3	0.35
Num. rotatable bonds	8
Num. H-bond acceptors	7
Num. H-bond donors	0
Molar Refractivity	133.77
TPSA	82.62 Å²
Lipophilicity	
Log $P_{o/w}$ (iLOGP)	4.12
Log $P_{o/w}$ (XLOGP3)	3.77
Log $P_{o/w}$ (WLOGP)	3.59
Log $P_{o/w}$ (MLOGP)	2.58
Log $P_{o/w}$ (SILICOS-IT)	4.13
Consensus Log $P_{o/w}$	3.64
Water Solubility	
Log S (ESOL)	-5.00
Solubility	4.61e-03 mg/ml; 1.00e-05 mol/l
Class	Moderately soluble
Pharmacokinetics	
GI absorption	High
BBB permeant	No
P-gp substrate	Yes
CYP1A2 inhibitor	No
CYP2C19 inhibitor	Yes
CYP2C9 inhibitor	Yes
CYP2D6 inhibitor	Yes
CYP3A4 inhibitor	Yes
Log K_p (skin permeation)	-6.43 cm/s
Druglikeness	
Lipinski	Yes; 0 violation
Ghose	No; 1 violation: MR>130
Veber	Yes
Egan	Yes
Muegge	Yes
Bioavailability Score	0.55

Table S19. ADMET properties of compound **1j.**

Compound 1j

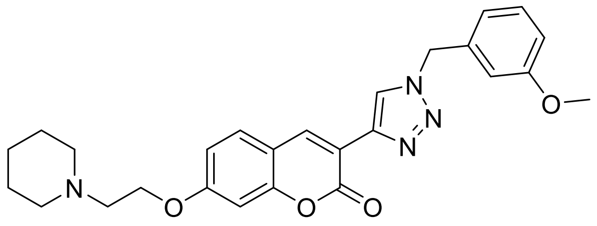

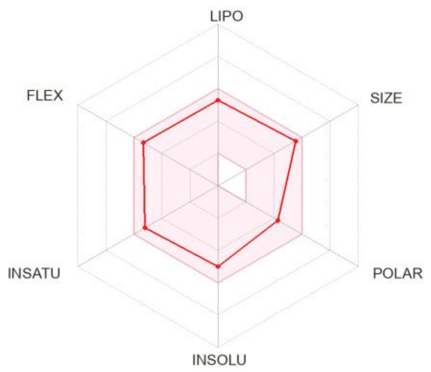

Physicochemical Properties	
Formula	C26H28N4O4
Molecular weight	460.52 g/mol
Fraction Csp3	0.35
Num. rotatable bonds	8
Num. H-bond acceptors	7
Num. H-bond donors	0
Molar Refractivity	133.77
TPSA	82.62 Å²
Lipophilicity	
Log $P_{o/w}$ (iLOGP)	4.07
Log $P_{o/w}$ (XLOGP3)	3.77
Log $P_{o/w}$ (WLOGP)	3.59
Log $P_{o/w}$ (MLOGP)	2.58
Log $P_{o/w}$ (SILICOS-IT)	4.13
Consensus Log $P_{o/w}$	3.63
Water Solubility	
Log S (ESOL)	-5.00
Solubility	4.61e-03 mg/ml; 1.00e-05 mol/l
Class	Moderately soluble
Pharmacokinetics	
GI absorption	High
BBB permeant	No
P-gp substrate	Yes
CYP1A2 inhibitor	No
CYP2C19 inhibitor	Yes
CYP2C9 inhibitor	Yes
CYP2D6 inhibitor	Yes
CYP3A4 inhibitor	Yes
Log K_p (skin permeation)	-6.43 cm/s
Druglikeness	
Lipinski	Yes; 0 violation
Ghose	No; 1 violation: MR>130
Veber	Yes
Egan	Yes
Muegge	Yes
Bioavailability Score	0.55

Table S20. ADMET properties of compound **1k.**

Compound 1k

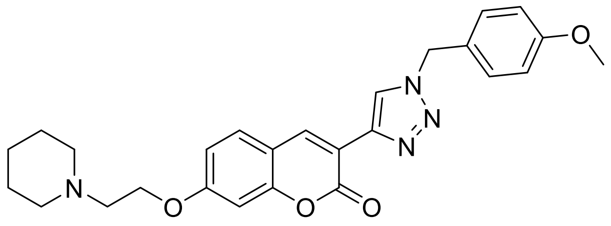

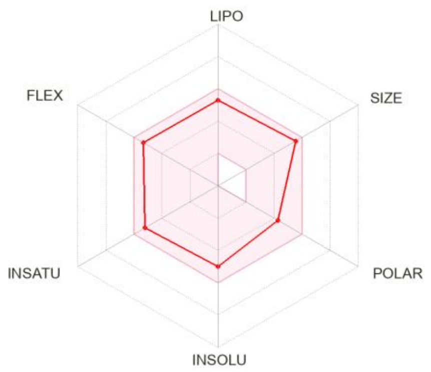

Physicochemical Properties	
Formula	C26H28N4O4
Molecular weight	460.52 g/mol
Fraction Csp3	0.35
Num. rotatable bonds	8
Num. H-bond acceptors	7
Num. H-bond donors	0
Molar Refractivity	133.77
TPSA	82.62 Å²
Lipophilicity	
Log $P_{o/w}$ (iLOGP)	4.07
Log $P_{o/w}$ (XLOGP3)	3.77
Log $P_{o/w}$ (WLOGP)	3.59
Log $P_{o/w}$ (MLOGP)	2.58
Log $P_{o/w}$ (SILICOS-IT)	4.13
Consensus Log $P_{o/w}$	3.63
Water Solubility	
Log *S* (ESOL)	-5.00
Solubility	4.61e-03 mg/ml; 1.00e-05 mol/l
Class	Moderately soluble
Pharmacokinetics	
GI absorption	High
BBB permeant	No
P-gp substrate	Yes
CYP1A2 inhibitor	No
CYP2C19 inhibitor	Yes
CYP2C9 inhibitor	Yes
CYP2D6 inhibitor	Yes
CYP3A4 inhibitor	Yes
Log K_p (skin permeation)	-6.43 cm/s
Druglikeness	
Lipinski	Yes; 0 violation
Ghose	No; 1 violation: MR>130
Veber	Yes
Egan	Yes
Muegge	Yes
Bioavailability Score	0.55

Table S21. ADMET properties of compound **1l.**

Compound 1l

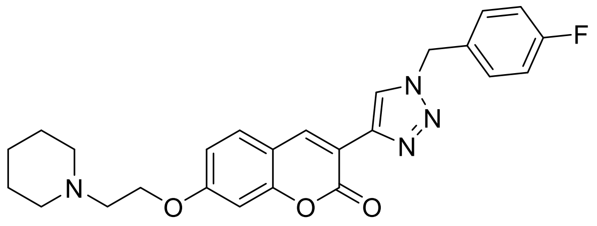

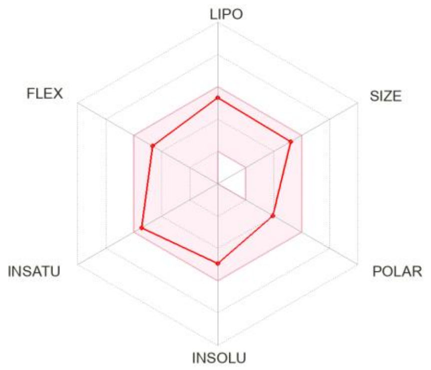

**Physicochemical Properties**	
Formula	C25H25FN4O3
Molecular weight	448.49 g/mol
Fraction Csp3	0.32
Num. rotatable bonds	7
Num. H-bond acceptors	7
Num. H-bond donors	0
Molar Refractivity	127.24
TPSA	73.39 Å^2
**Lipophilicity**	
Log $P_{o/w}$ (iLOGP)	4.39
Log $P_{o/w}$ (XLOGP3)	3.90
Log $P_{o/w}$ (WLOGP)	4.14
Log $P_{o/w}$ (MLOGP)	3.26
Log $P_{o/w}$ (SILICOS-IT)	4.49
Consensus Log $P_{o/w}$	4.04
**Water Solubility**	
Log S (ESOL)	-5.09
Solubility	3.67e-03 mg/ml; 8.19e-06 mol/l
Class	Moderately soluble
**Pharmacokinetics**	
GI absorption	High
BBB permeant	Yes
P-gp substrate	Yes
CYP1A2 inhibitor	Yes
CYP2C19 inhibitor	Yes
CYP2C9 inhibitor	Yes
CYP2D6 inhibitor	Yes
CYP3A4 inhibitor	Yes
Log K_p (skin permeation)	-6.27 cm/s
**Druglikeness**	
Lipinski	Yes; 0 violation
Ghose	Yes
Veber	Yes
Egan	Yes
Muegge	Yes
Bioavailability Score	0.55

Table S22. ADMET properties of compound **1m.**

Compound 1m

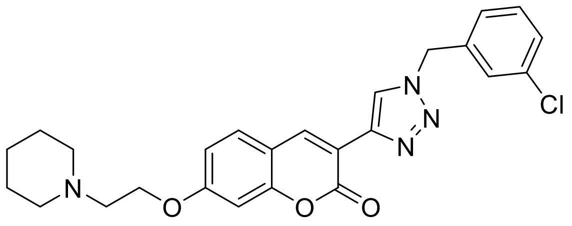

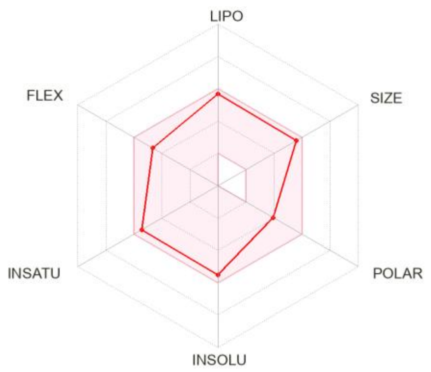

Physicochemical Properties	
Formula	C25H25ClN4O3
Molecular weight	464.94 g/mol
Fraction Csp3	0.32
Num. rotatable bonds	7
Num. H-bond acceptors	6
Num. H-bond donors	0
Molar Refractivity	132.29
TPSA	73.39 Å²
Lipophilicity	
Log $P_{o/w}$ (iLOGP)	4.35
Log $P_{o/w}$ (XLOGP3)	4.42
Log $P_{o/w}$ (WLOGP)	4.24
Log $P_{o/w}$ (MLOGP)	3.36
Log $P_{o/w}$ (SILICOS-IT)	4.71
Consensus Log $P_{o/w}$	4.22
Water Solubility	
Log *S* (ESOL)	-5.52
Solubility	1.42e-03 mg/ml; 3.05e-06 mol/l
Class	Moderately soluble
Pharmacokinetics	
GI absorption	High
BBB permeant	Yes
P-gp substrate	Yes
CYP1A2 inhibitor	Yes
CYP2C19 inhibitor	Yes
CYP2C9 inhibitor	Yes
CYP2D6 inhibitor	Yes
CYP3A4 inhibitor	Yes
Log K_p (skin permeation)	-6.00 cm/s
Druglikeness	
Lipinski	Yes; 0 violation
Ghose	No; 1 violation: MR>130
Veber	Yes
Egan	Yes
Muegge	Yes
Bioavailability Score	0.55

Table S23. ADMET properties of compound **1n.**

**Compound 1n**	
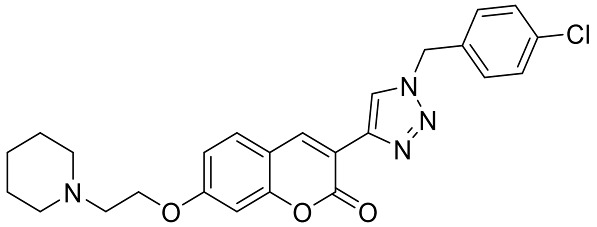	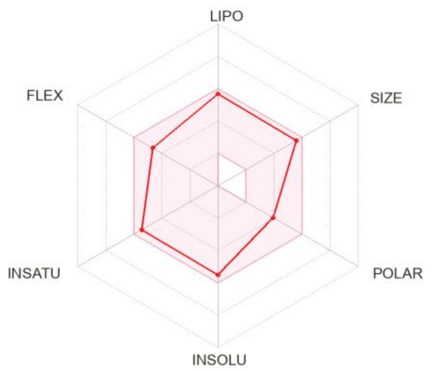
Physicochemical Properties	
Formula	C25H25ClN4O3
Molecular weight	464.94 g/mol
Fraction Csp3	0.32
Num. rotatable bonds	7
Num. H-bond acceptors	6
Num. H-bond donors	0
Molar Refractivity	132.29
TPSA	73.39 Å²
Lipophilicity	
Log $P_{o/w}$ (iLOGP)	4.24
Log $P_{o/w}$ (XLOGP3)	4.42
Log $P_{o/w}$ (WLOGP)	4.24
Log $P_{o/w}$ (MLOGP)	3.36
Log $P_{o/w}$ (SILICOS-IT)	4.71
Consensus Log $P_{o/w}$	4.19
Water Solubility	
Log S (ESOL)	-5.52
Solubility	1.42e-03 mg/ml; 3.05e-06 mol/l
Class	Moderately soluble
Pharmacokinetics	
GI absorption	High
BBB permeant	Yes
P-gp substrate	Yes
CYP1A2 inhibitor	Yes
CYP2C19 inhibitor	Yes
CYP2C9 inhibitor	Yes
CYP2D6 inhibitor	Yes
CYP3A4 inhibitor	Yes
Log K_p (skin permeation)	-6.00 cm/s
Druglikeness	
Lipinski	Yes; 0 violation
Ghose	No; 1 violation: MR>130
Veber	Yes
Egan	Yes
Muegge	Yes
Bioavailability Score	0.55

Table S24. ADMET properties of compound **1o.**

**Compound 1o**	
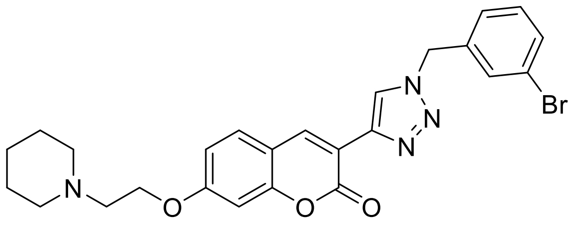	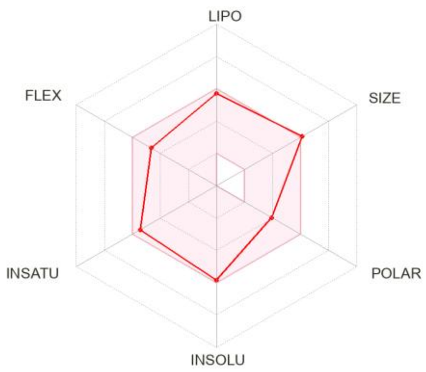
**Physicochemical Properties**	
Formula	C25H25BrN4O3
Molecular weight	509.40 g/mol
Fraction Csp3	0.32
Num. rotatable bonds	7
Num. H-bond acceptors	6
Num. H-bond donors	0
Molar Refractivity	134.98
TPSA	73.39 Å²
**Lipophilicity**	
Log $P_{o/w}$ (iLOGP)	4.46
Log $P_{o/w}$ (XLOGP3)	4.49
Log $P_{o/w}$ (WLOGP)	4.35
Log $P_{o/w}$ (MLOGP)	3.46
Log $P_{o/w}$ (SILICOS-IT)	4.75
Consensus Log $P_{o/w}$	4.30
**Water Solubility**	
Log S (ESOL)	-5.84
Solubility	7.43e-04 mg/ml; 1.46e-06 mol/l
Class	Moderately soluble
**Pharmacokinetics**	
GI absorption	High
BBB permeant	Yes
P-gp substrate	Yes
CYP1A2 inhibitor	Yes
CYP2C19 inhibitor	Yes
CYP2C9 inhibitor	Yes
CYP2D6 inhibitor	Yes
CYP3A4 inhibitor	Yes
Log K_p (skin permeation)	-6.22 cm/s
**Druglikeness**	
Lipinski	Yes; 1 violation: MW>500
Ghose	No; 2 violations: MW>480, MR>130
Veber	Yes
Egan	Yes
Muegge	Yes
Bioavailability Score	0.55

Table S25. ADMET properties of compound **1p.**

Compound 1p

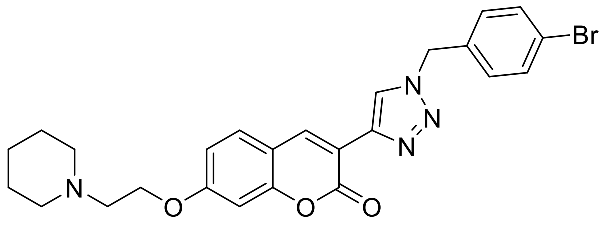

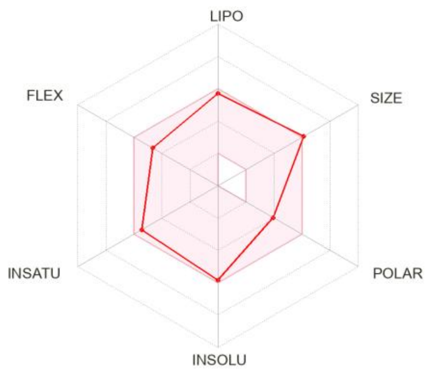

Physicochemical Properties	
Formula	C25H25BrN4O3
Molecular weight	509.40 g/mol
Fraction Csp3	0.32
Num. rotatable bonds	7
Num. H-bond acceptors	6
Num. H-bond donors	0
Molar Refractivity	134.98
TPSA	73.39 Å²
**Lipophilicity**	
Log $P_{o/w}$ (iLOGP)	4.29
Log $P_{o/w}$ (XLOGP3)	4.49
Log $P_{o/w}$ (WLOGP)	4.35
Log $P_{o/w}$ (MLOGP)	3.46
Log $P_{o/w}$ (SILICOS-IT)	4.75
Consensus Log $P_{o/w}$	4.27
**Water Solubility**	
Log S (ESOL)	-5.84
Solubility	7.43e-04 mg/ml; 1.46e-06 mol/l
Class	Moderately soluble
**Pharmacokinetics**	
GI absorption	High
BBB permeant	Yes
P-gp substrate	Yes
CYP1A2 inhibitor	Yes
CYP2C19 inhibitor	Yes
CYP2C9 inhibitor	Yes
CYP2D6 inhibitor	Yes
CYP3A4 inhibitor	Yes
Log K_p (skin permeation)	-6.22 cm/s
**Druglikeness**	
Lipinski	Yes; 1 violation: MW>500
Ghose	No; 2 violations: MW>480, MR>130
Veber	Yes
Egan	Yes
Muegge	Yes
Bioavailability Score	0.55

Table S26. ADMET properties of compound **1q.**

Compound 1q

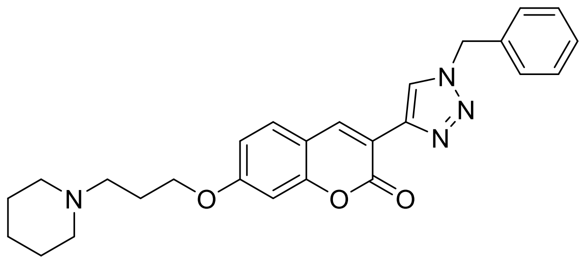

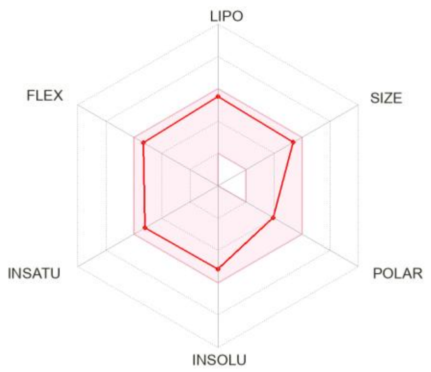

Physicochemical Properties	
Formula	C26H28N4O3
Molecular weight	444.50 g/mol
Fraction Csp3	0.35
Num. rotatable bonds	8
Num. H-bond acceptors	6
Num. H-bond donors	0
Molar Refractivity	132,09
TPSA	73.39 Å²
**Lipophilicity**	
Log $P_{o/w}$ (iLOGP)	4.28
Log $P_{o/w}$ (XLOGP3)	4.15
Log $P_{o/w}$ (WLOGP)	3.97
Log $P_{o/w}$ (MLOGP)	3.10
Log $P_{o/w}$ (SILICOS-IT)	4.47
Consensus Log $P_{o/w}$	3.99
**Water Solubility**	
Log S (ESOL)	-5.15
Solubility	3.12e-03 mg/ml; 7.02e-06 mol/l
Class	Moderately soluble
**Pharmacokinetics**	
GI absorption	High
BBB permeant	Yes
P-gp substrate	Yes
CYP1A2 inhibitor	Yes
CYP2C19 inhibitor	Yes
CYP2C9 inhibitor	Yes
CYP2D6 inhibitor	Yes
CYP3A4 inhibitor	Yes
Log K_p (skin permeation)	-6.07 cm/s
**Druglikeness**	
Lipinski	Yes; 0 violation
Ghose	No; 1 violation: MR>130
Veber	Yes
Egan	Yes
Muegge	Yes
Bioavailability Score	0.55

Copies of ^{1}H, ^{13}C NMR

Figure S18. ^{1}H NMR spectra of **6a.**

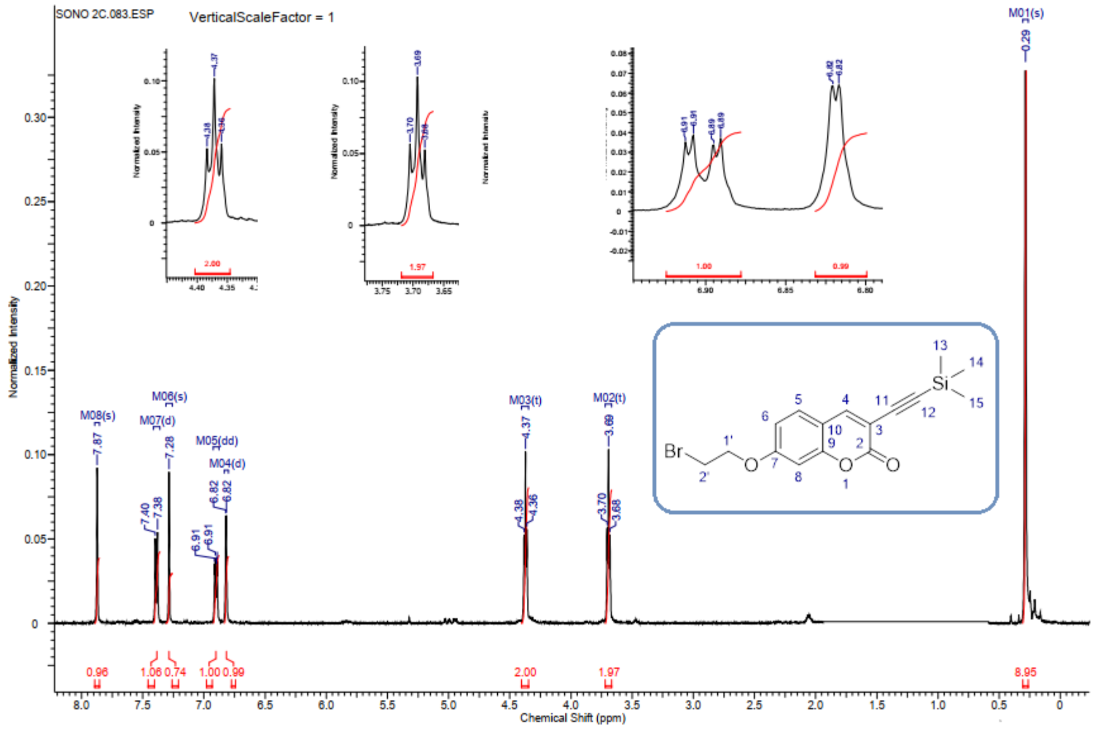

Figure S19. ^{13}C NMR spectra of **6a.**

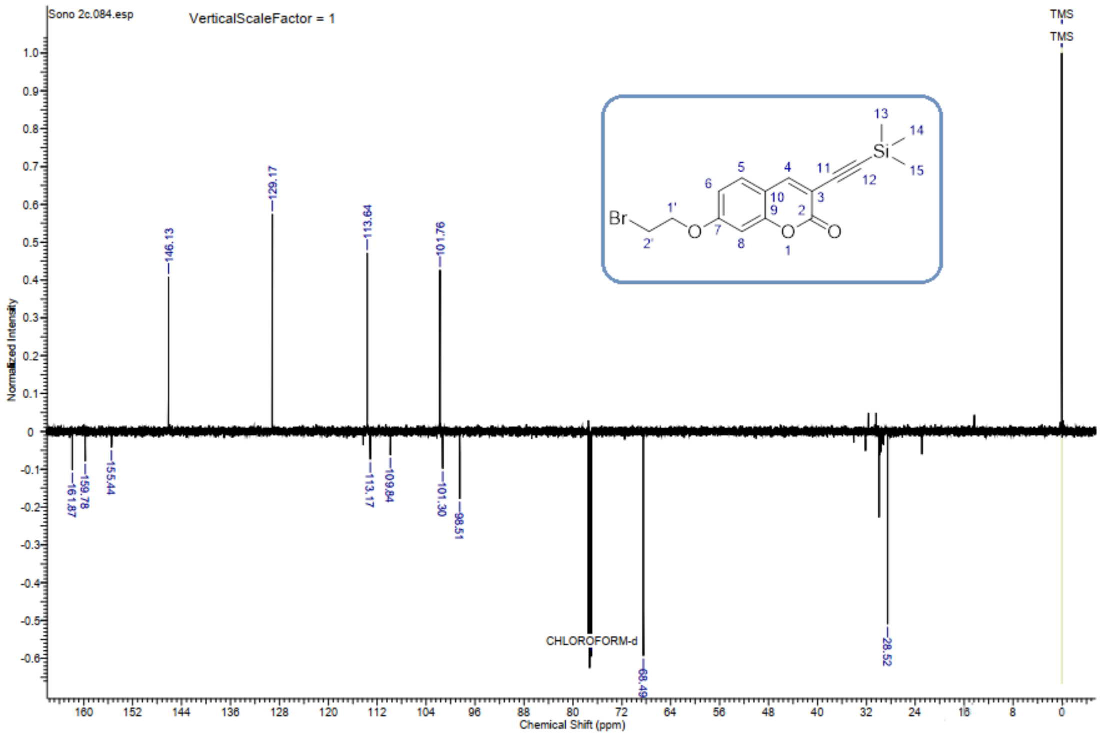

Figure S20. ^{1}H NMR spectra of **6b.**

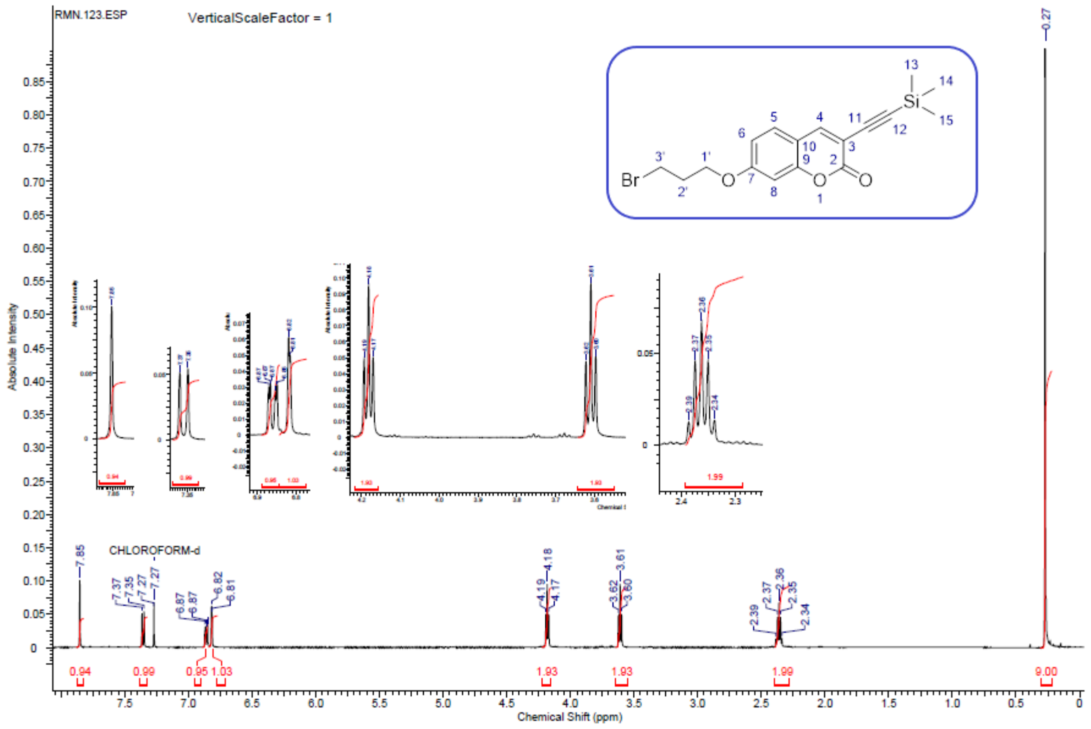

Figure S21. ^{13}C NMR spectra of **6b.**

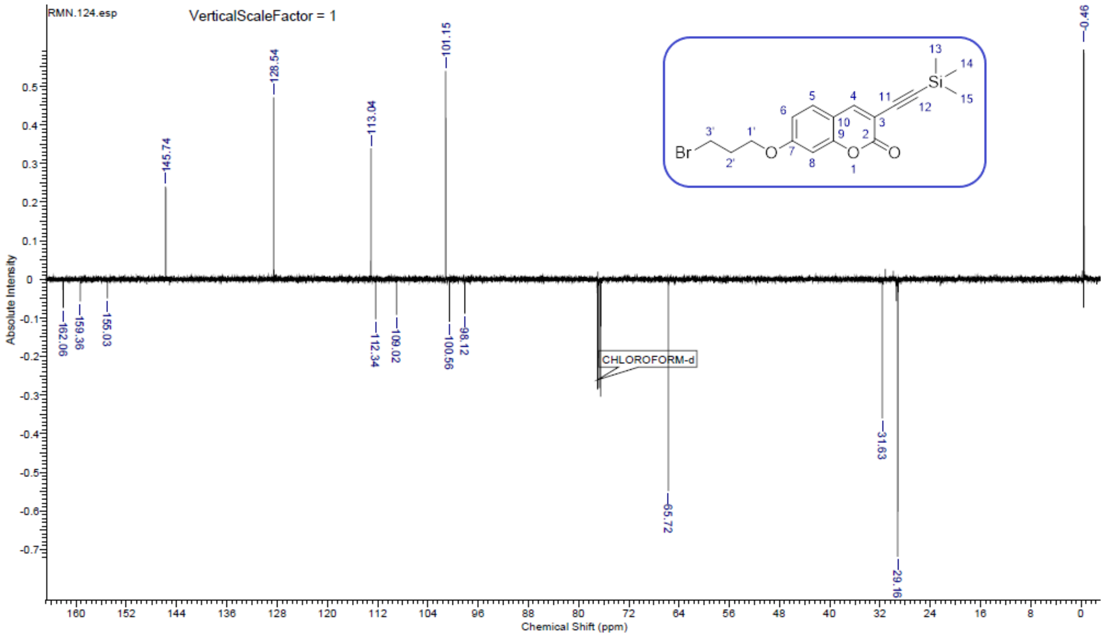

Figure S22. ^{1}H NMR spectra of **6c.**

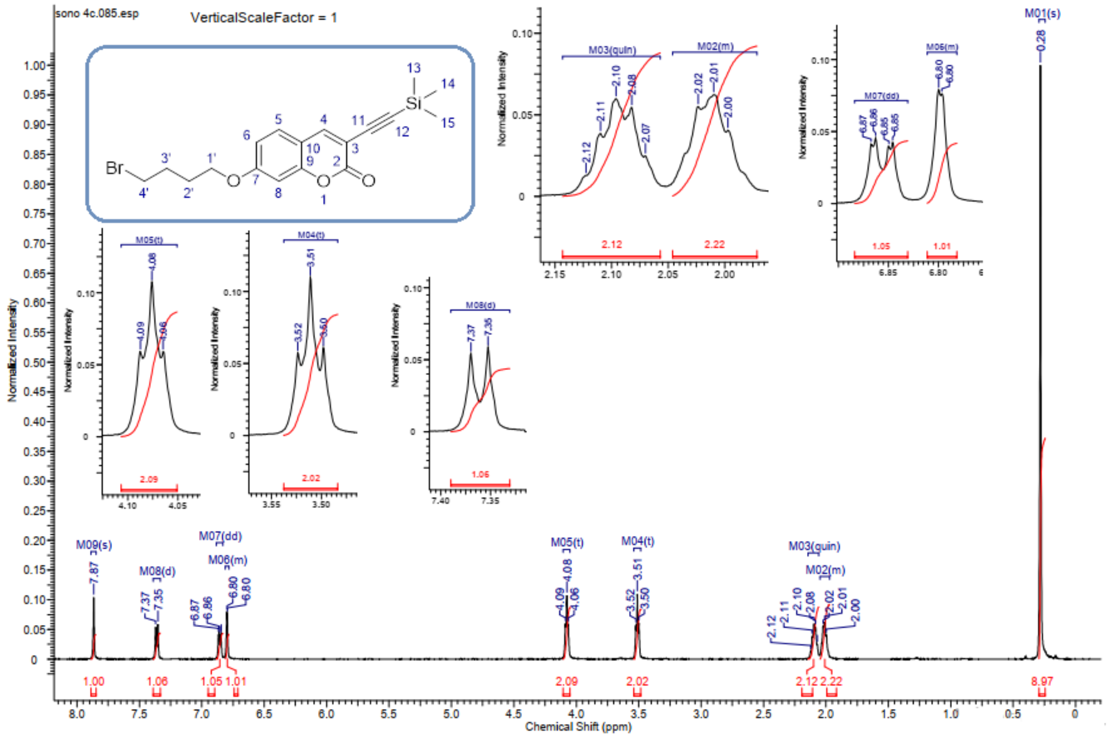

Figure S23. ^{13}C NMR spectra of **6c.**

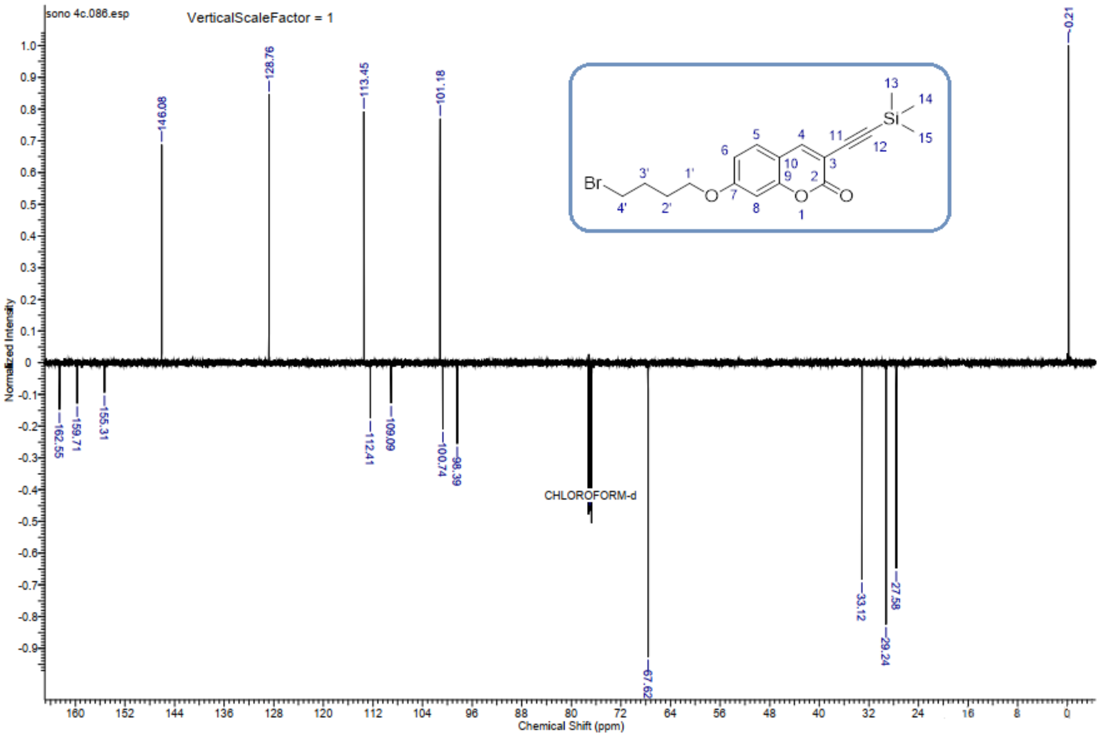

Figure S24. ^{1}H NMR spectra of **6d.**

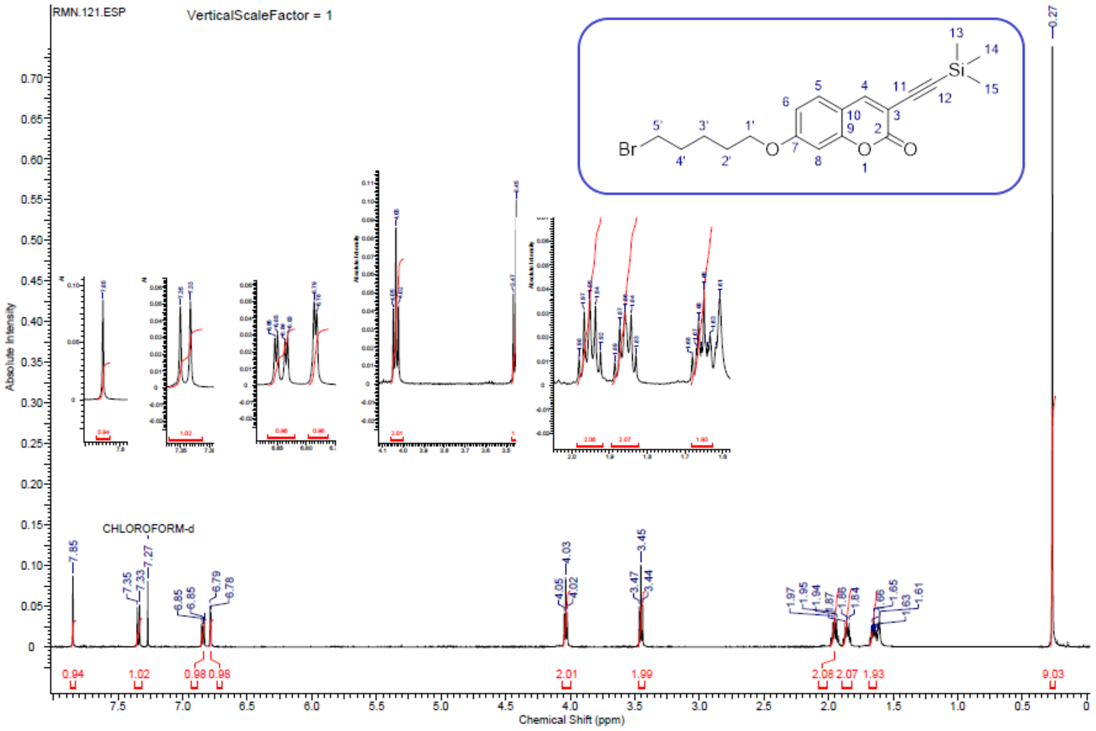

Figure S25. ^{13}C NMR spectra of **6d.**

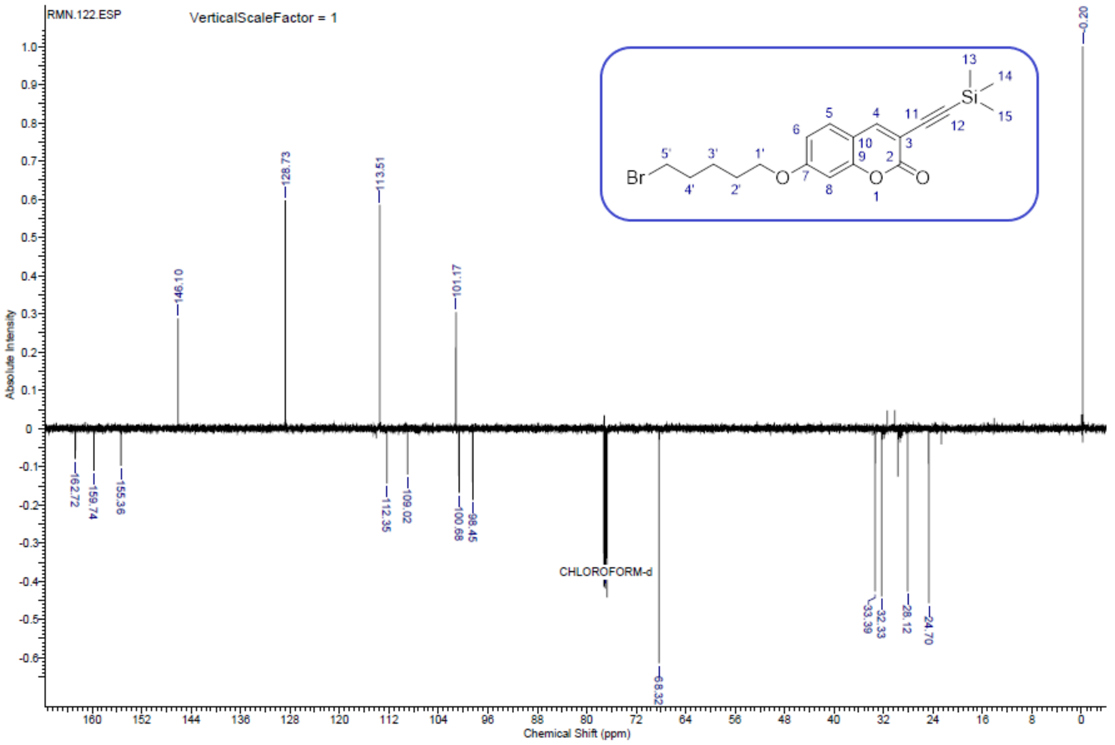

Figure S26. ^{1}H NMR spectra of **14a.**

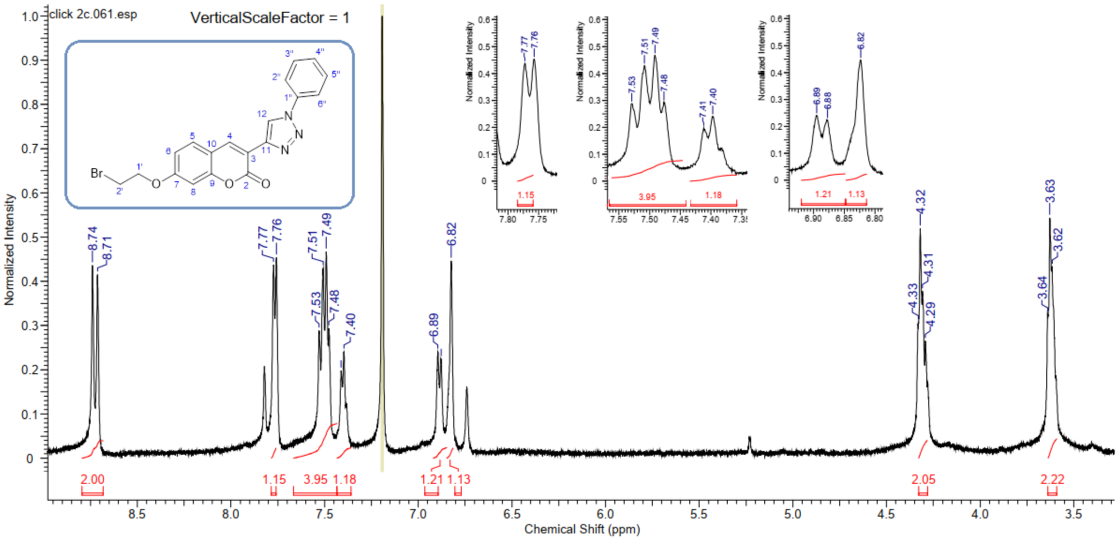

Figure S27. ^{13}C NMR spectra of **14a.**

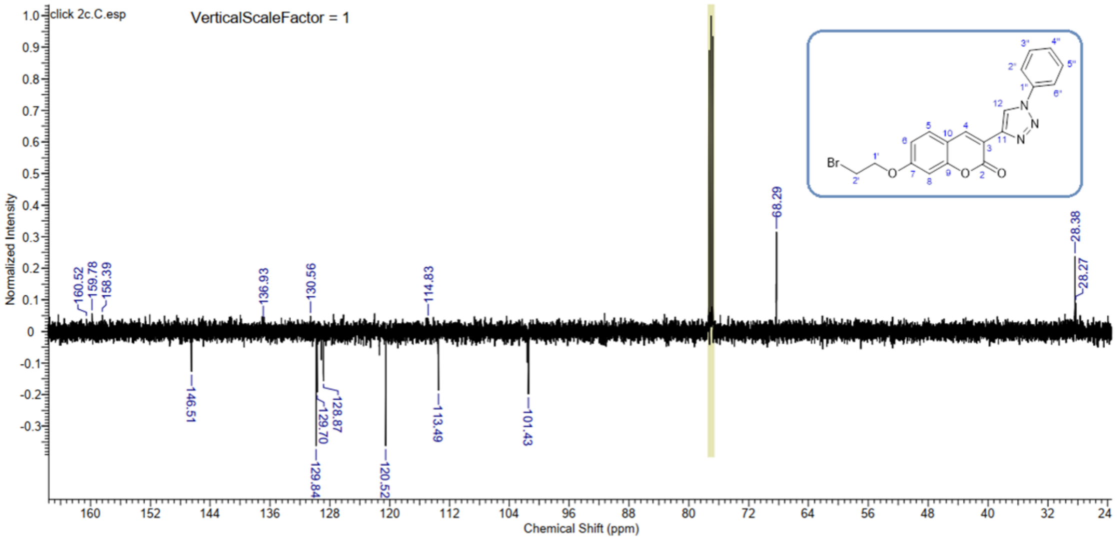

Figure S28. ^{1}H NMR spectra of **14b.**

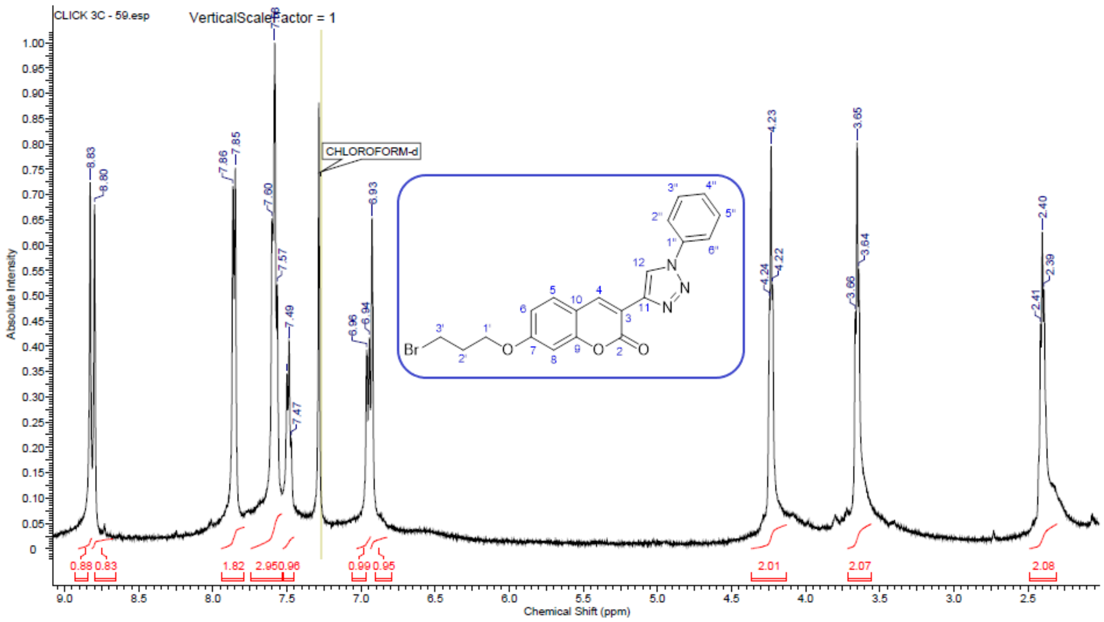

Figure S29. ^{13}C NMR spectra of **14b.**

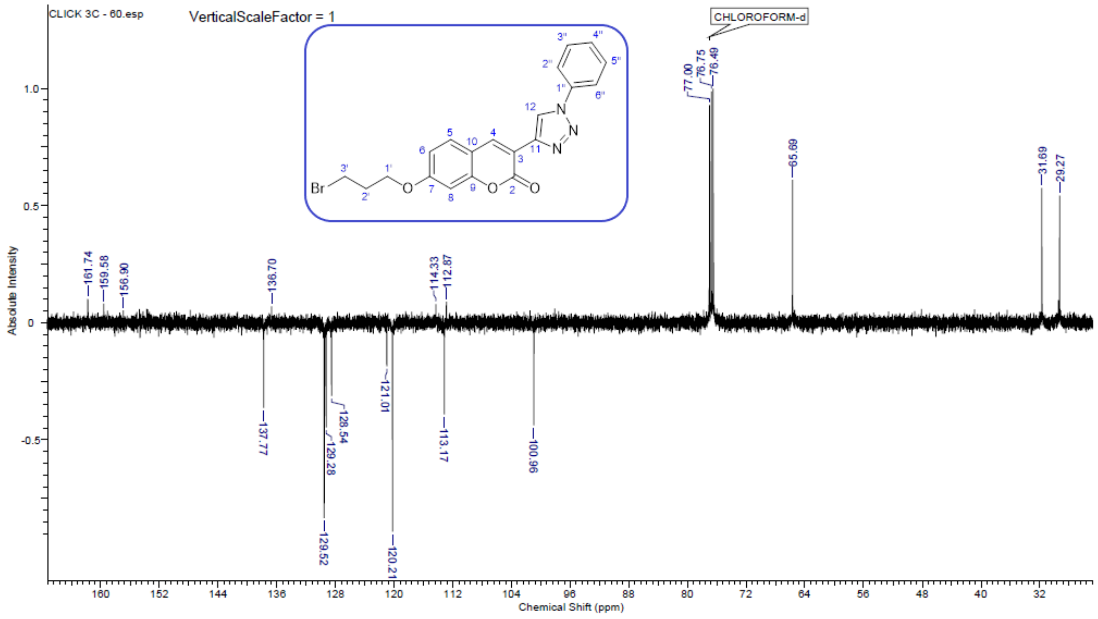

Figure S30. ^{1}H NMR spectra of **14c.**

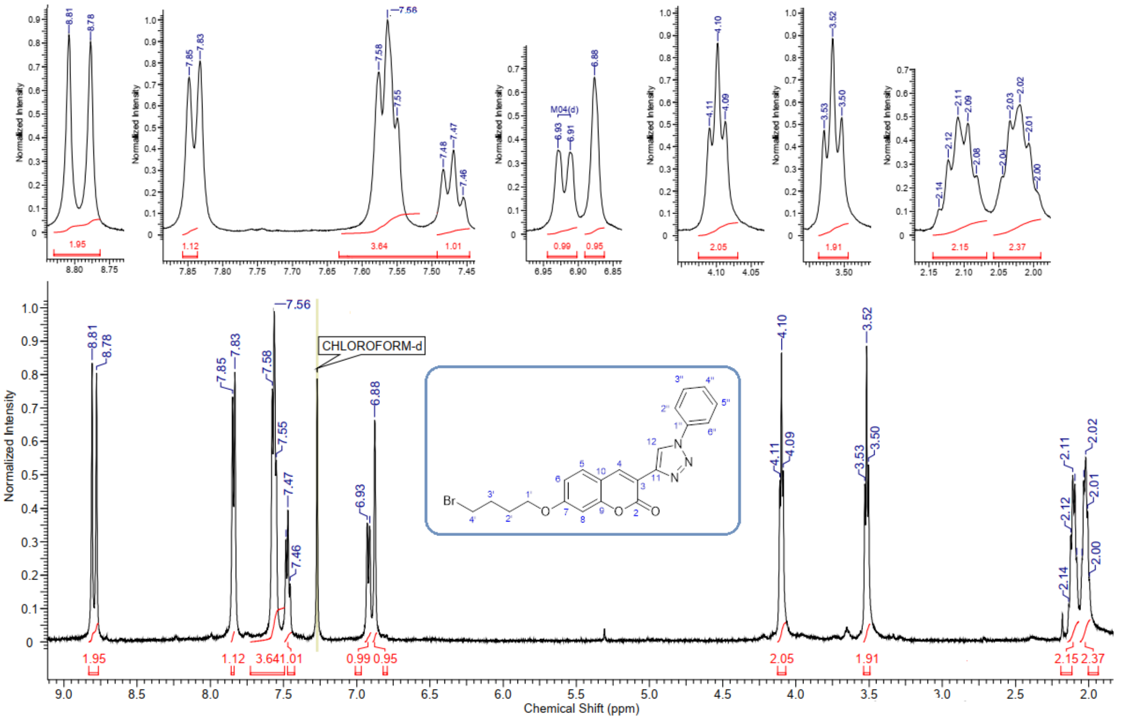

Figure S31. ^{13}C NMR spectra of **14c.**

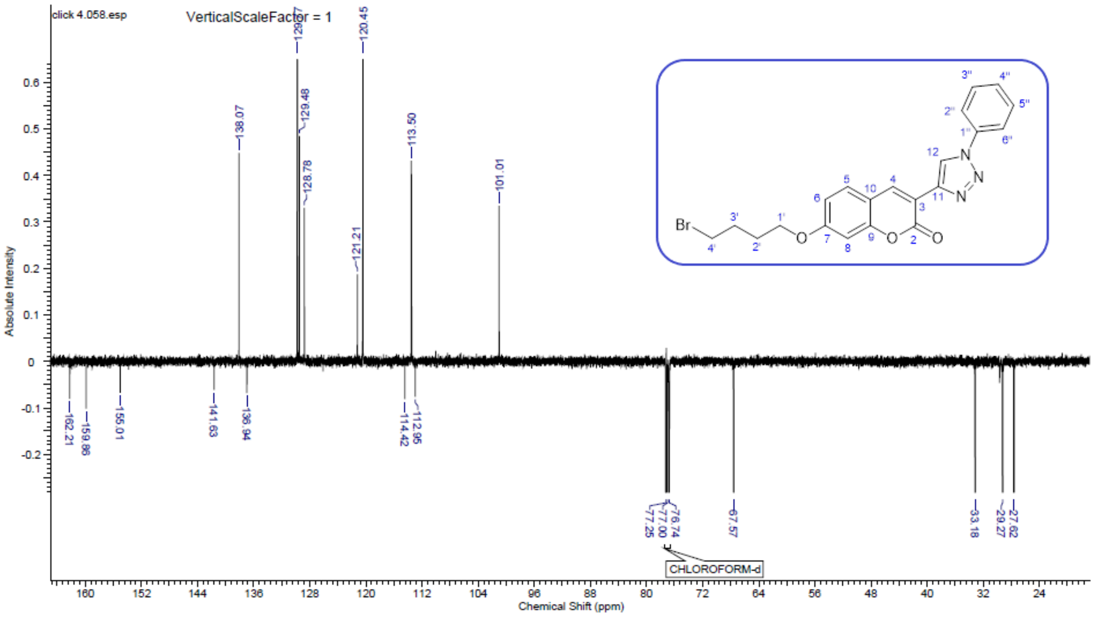

Figure S32. ^{1}H NMR spectra of **14d.**

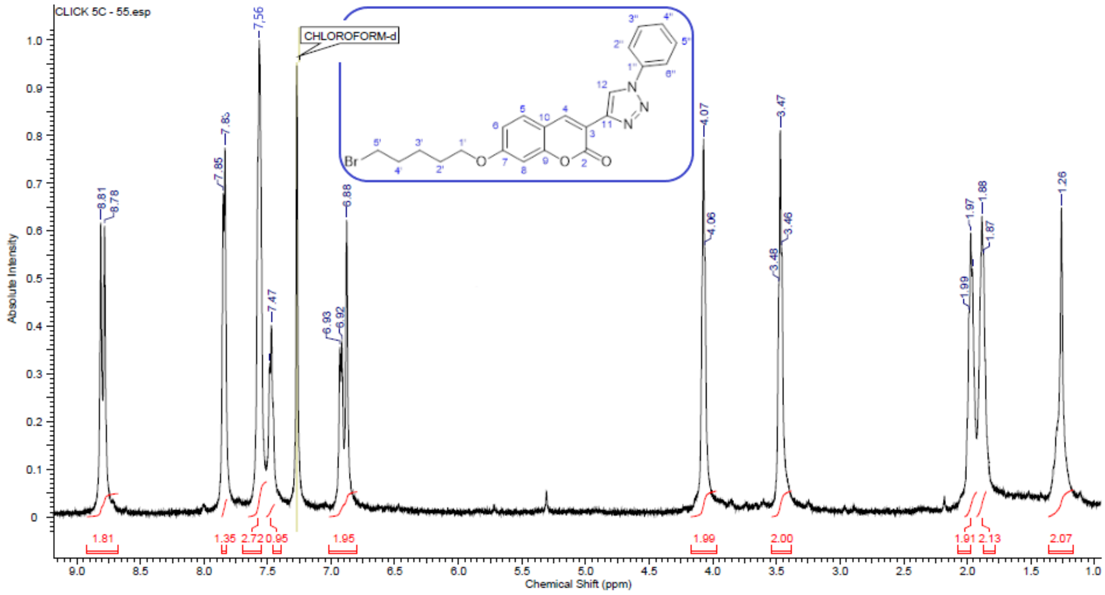

Figure S33. ^{13}C NMR spectra of **14d.**

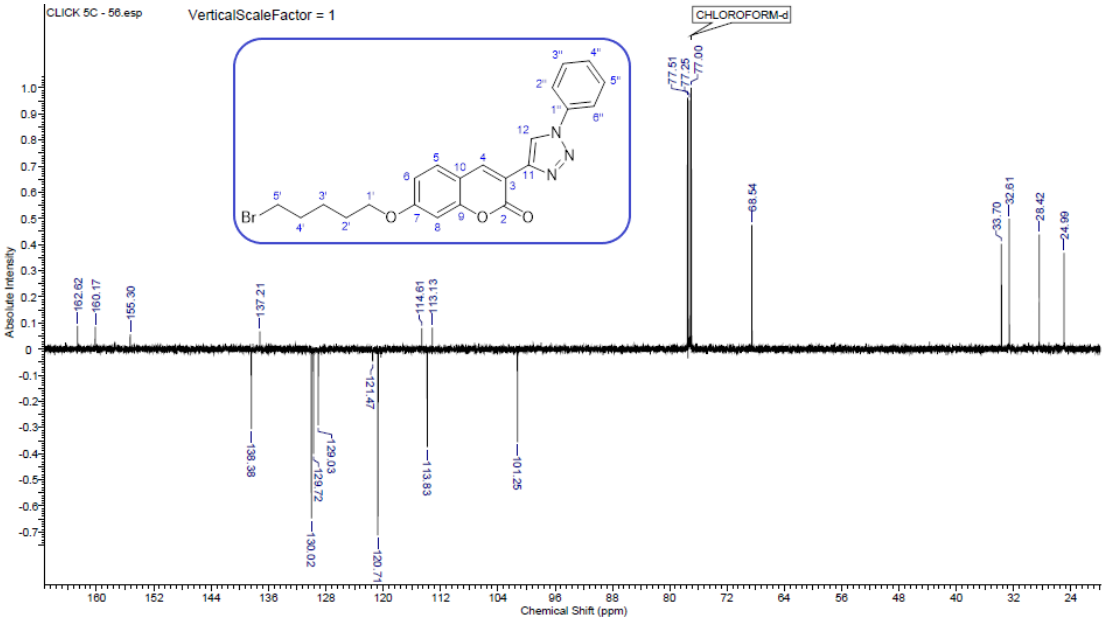

Figure S34. ^{1}H NMR spectra of **14e.**

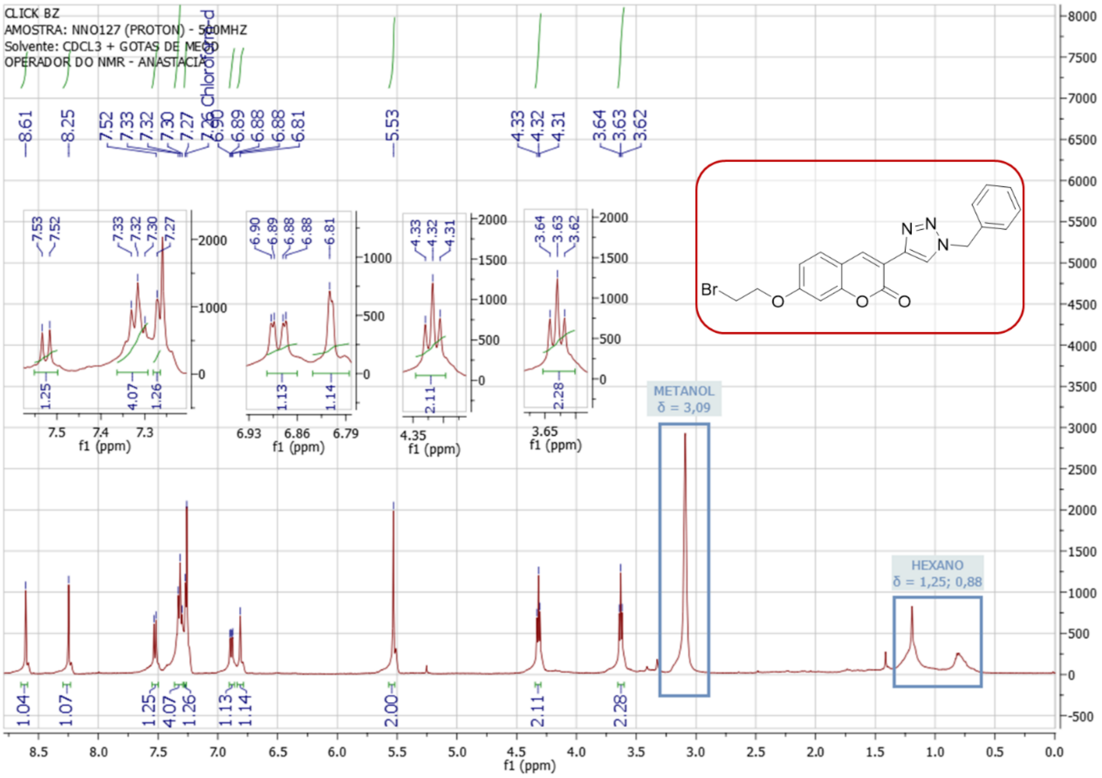

Figure S35. ^{13}C NMR spectra of **14e.**

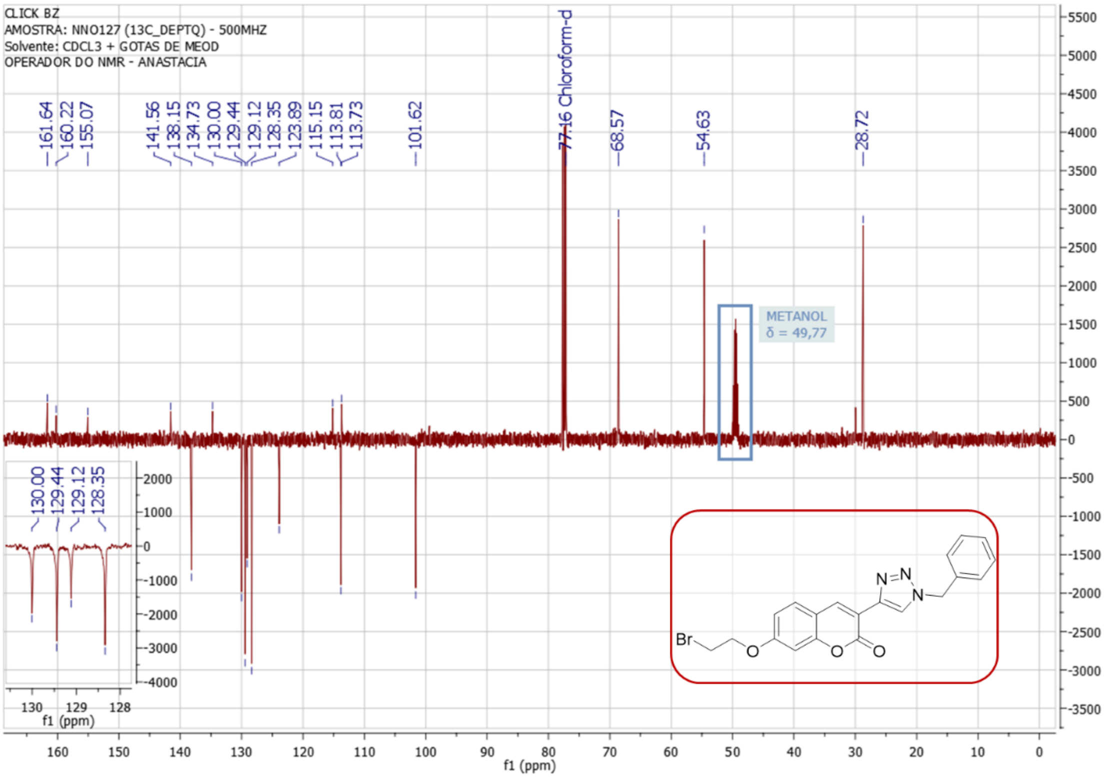

Figure S36. ^{1}H NMR spectra of **14f.**

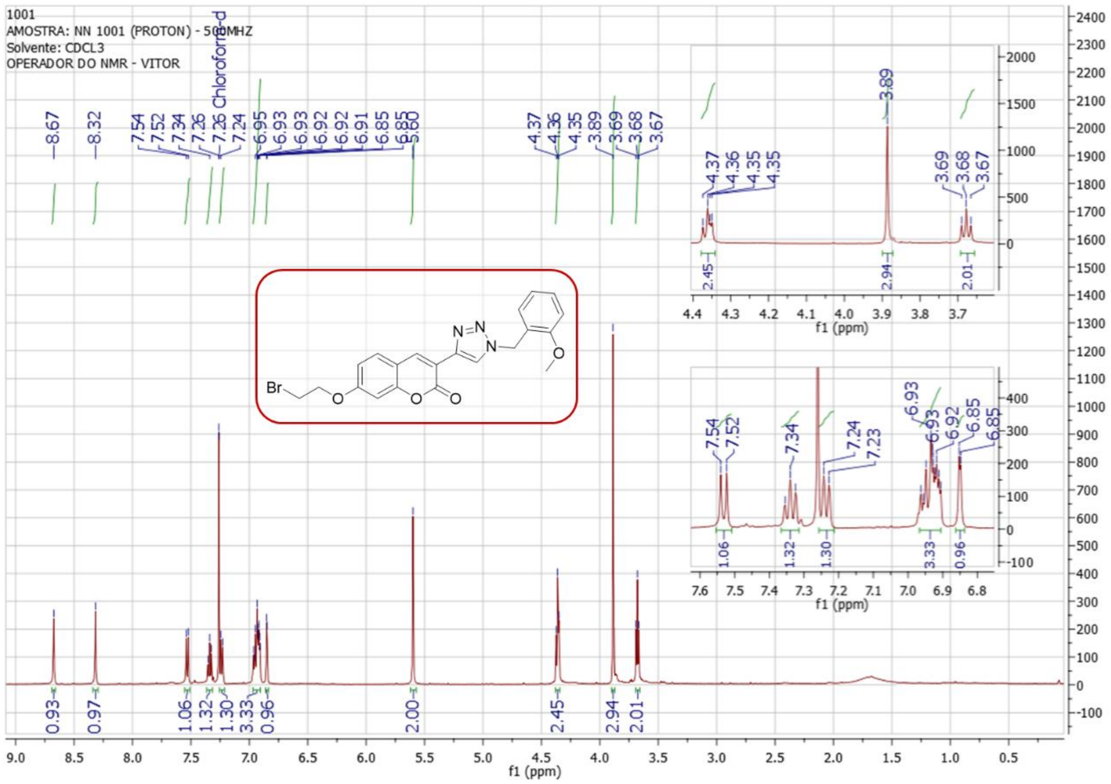

Figure S37. ^{13}C NMR spectra of **14f.**

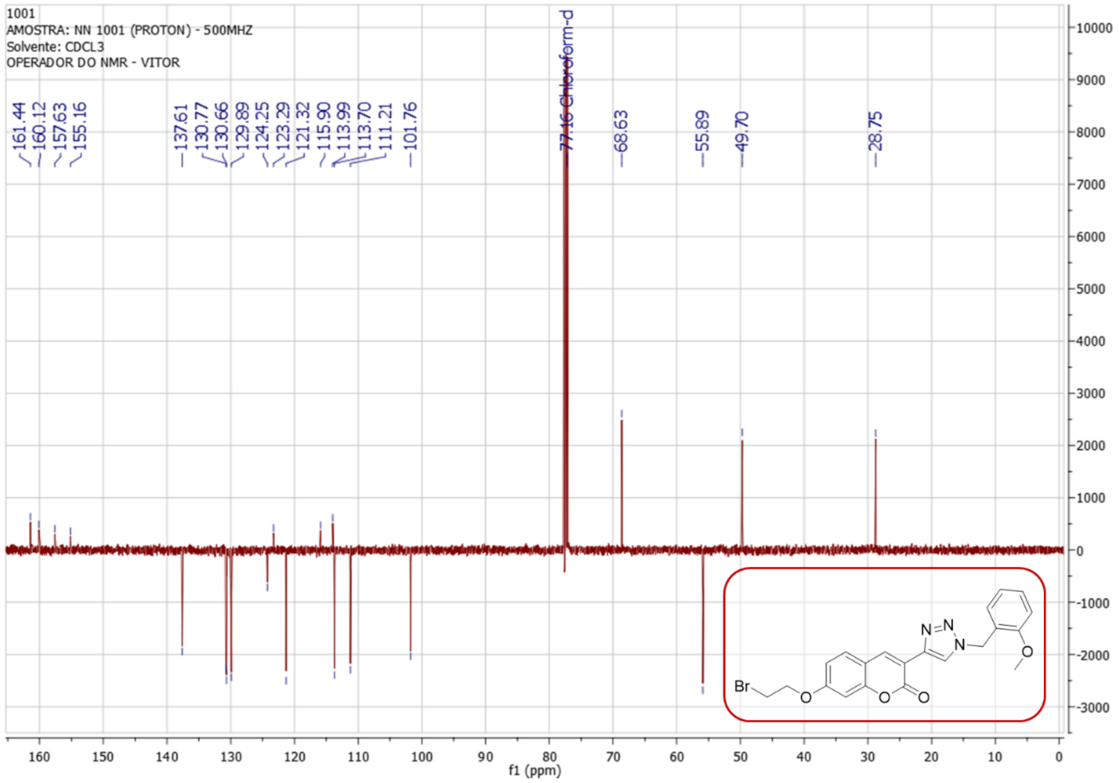

Figure S38. ^{1}H NMR spectra of **14g.**

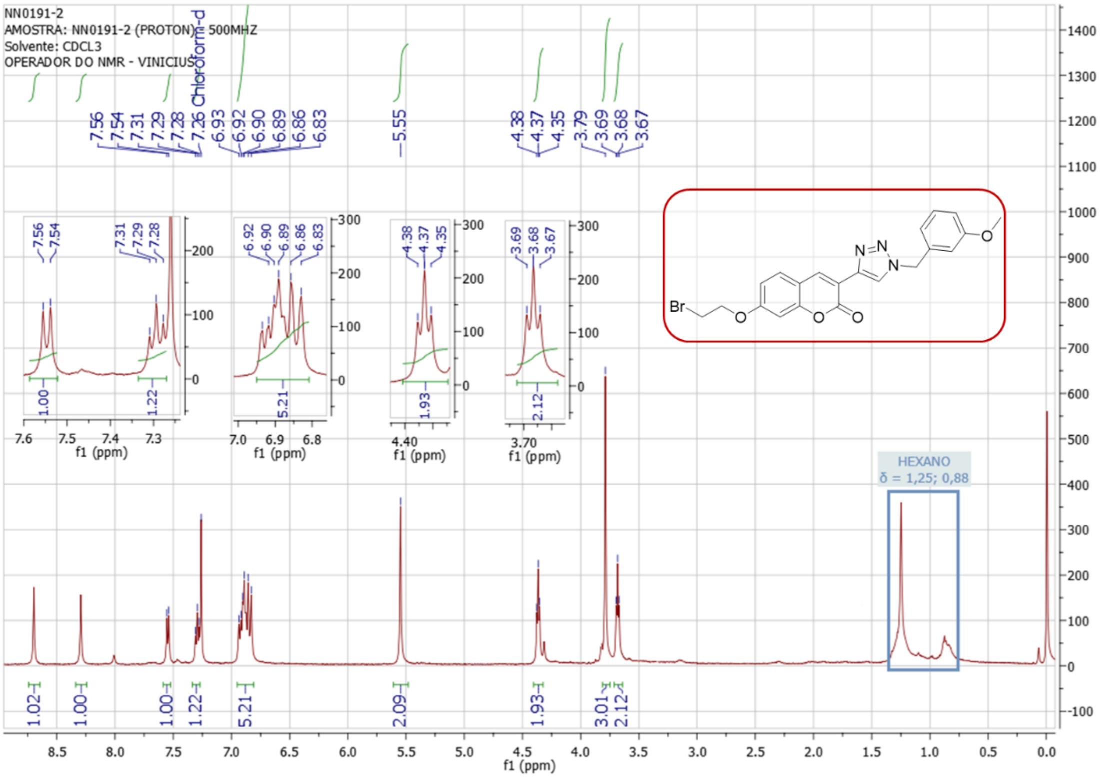

Figure S39. ^{13}C NMR spectra of **14g.**

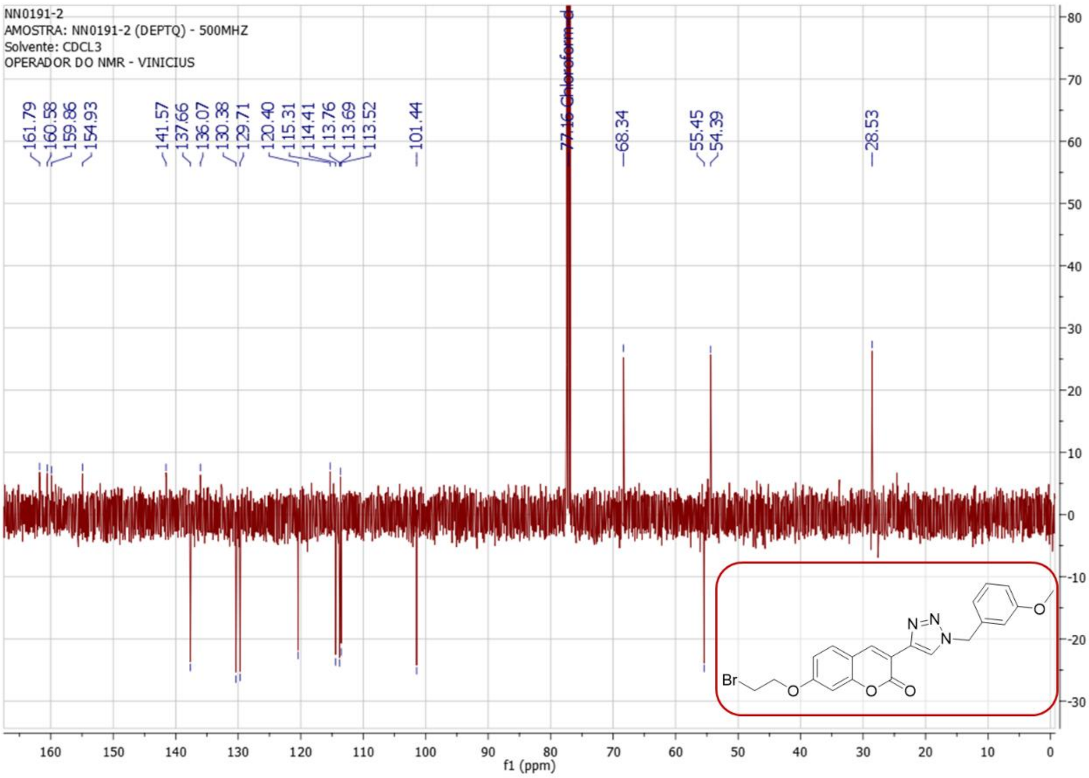

Figure S40. ^{1}H NMR spectra of **14h.**

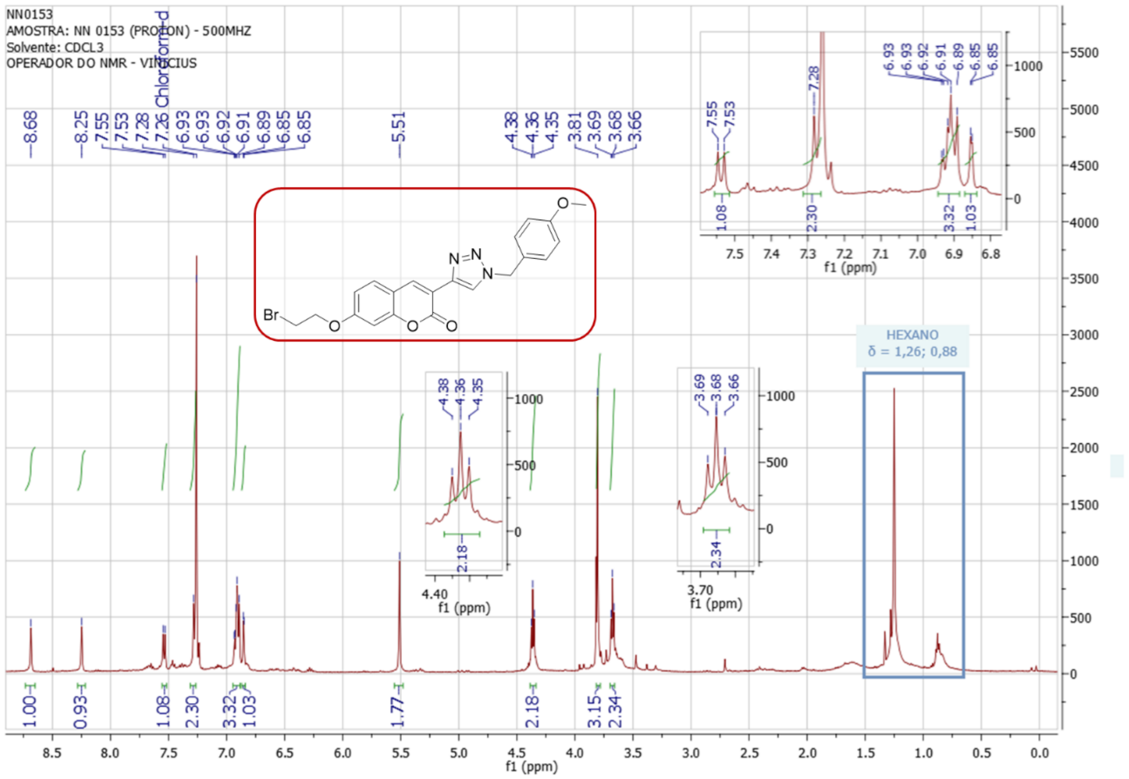

Figure S41. ^{13}C NMR spectra of **14h.**

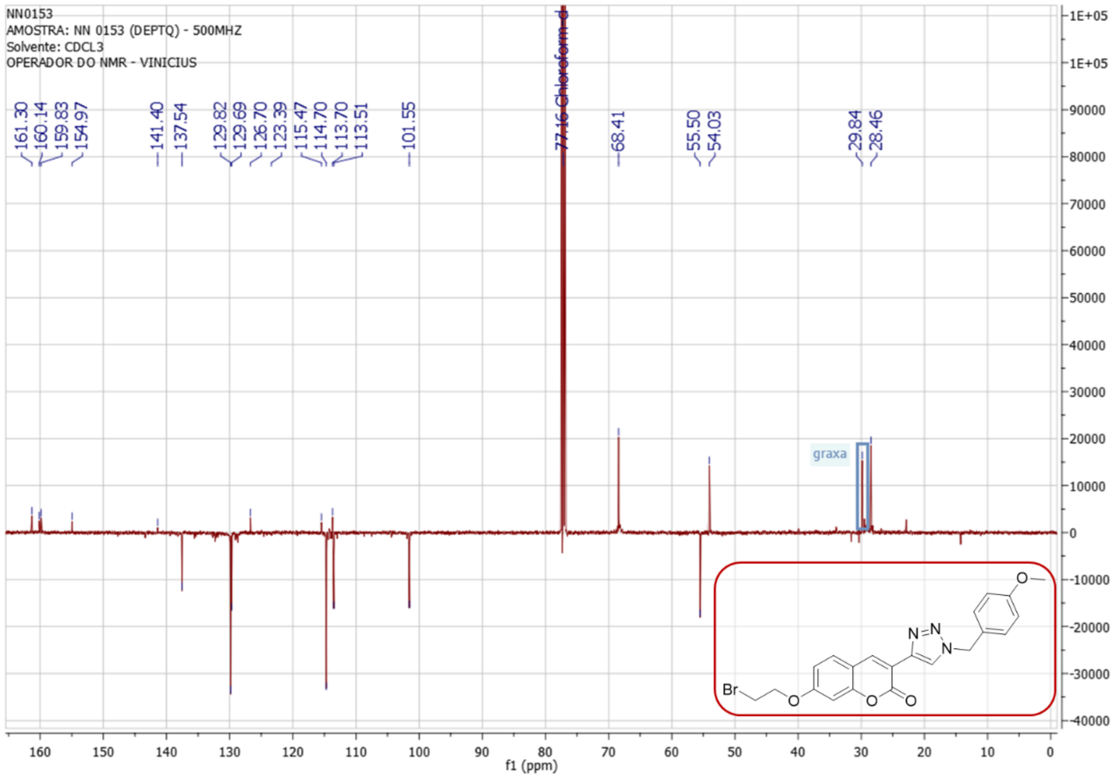

Figure S42. ^{1}H NMR spectra of **14i.**

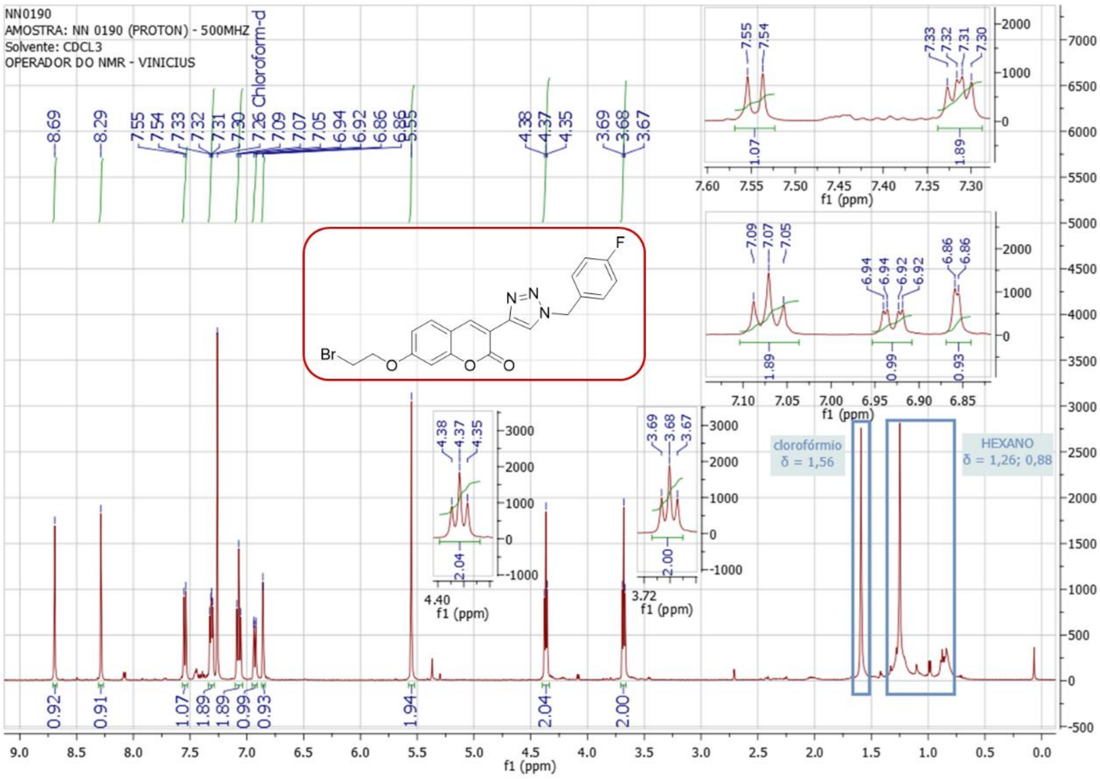

Figure S43. ^{13}C NMR spectra of **14i.**

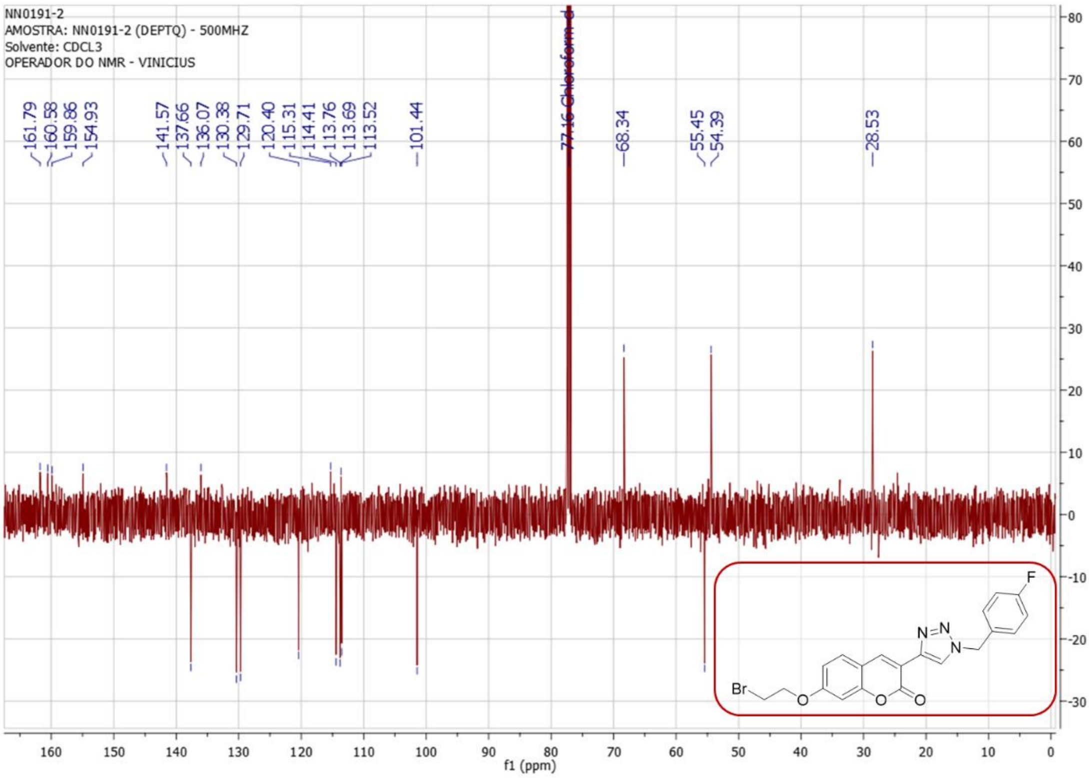

Figure S44. ^{1}H NMR spectra of **14j.**

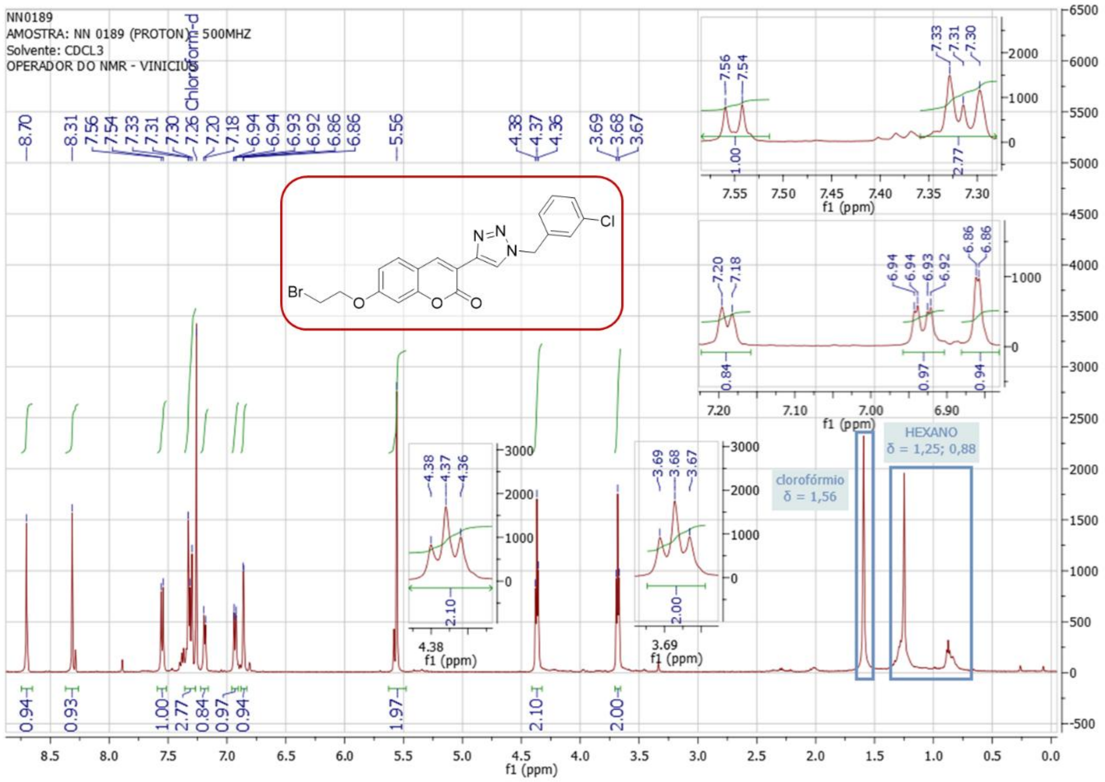

Figure S45. ^{13}C NMR spectra of **14j.**

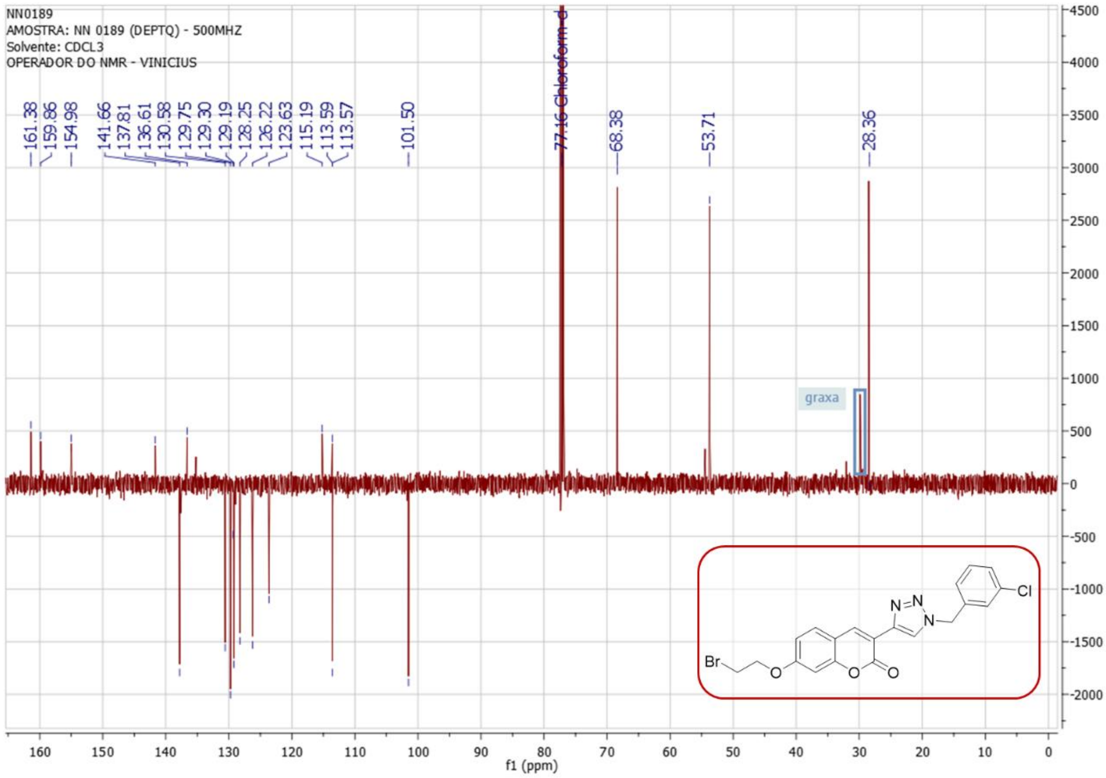

Figure S46. ^{1}H NMR spectra of **14k.**

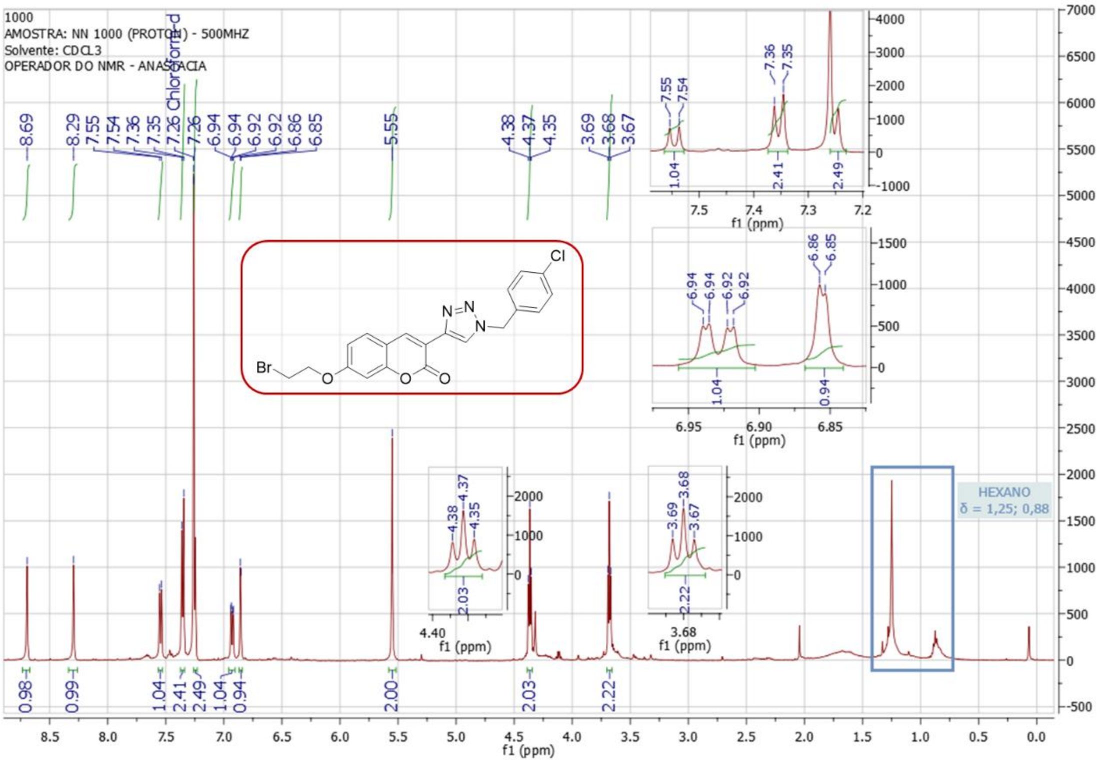

Figure S47. ^{13}C NMR spectra of **14k.**

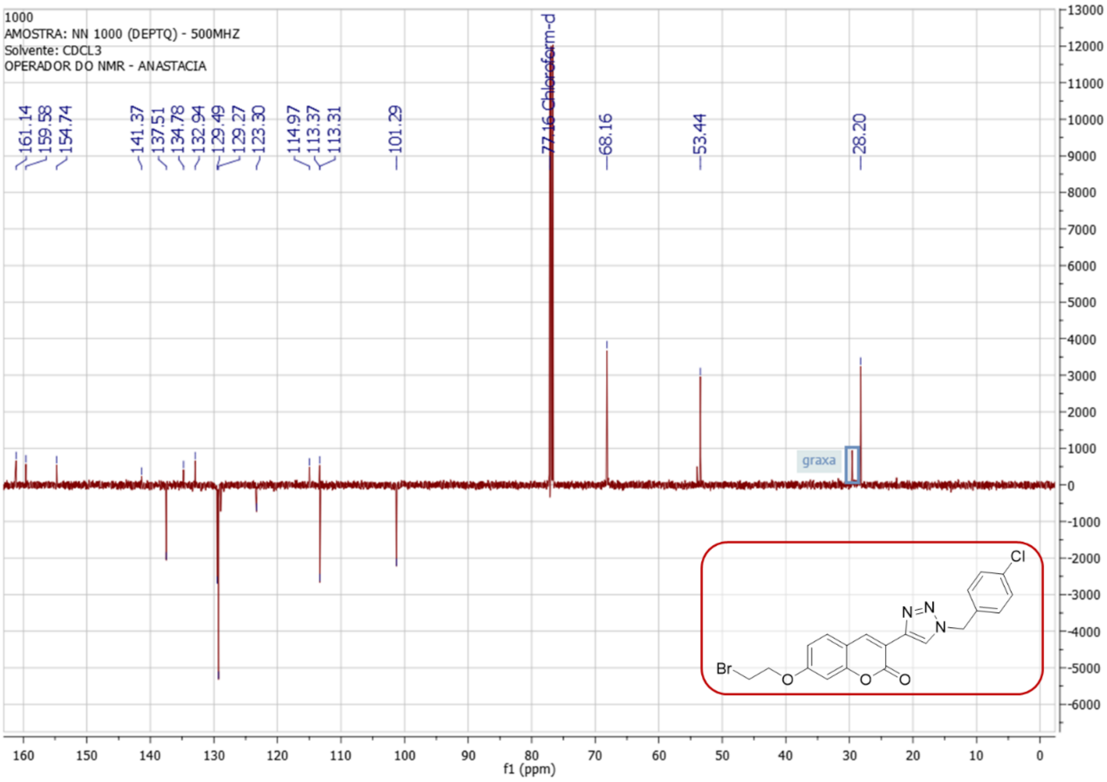

Figure S48. ^{1}H NMR spectra of **14l.**

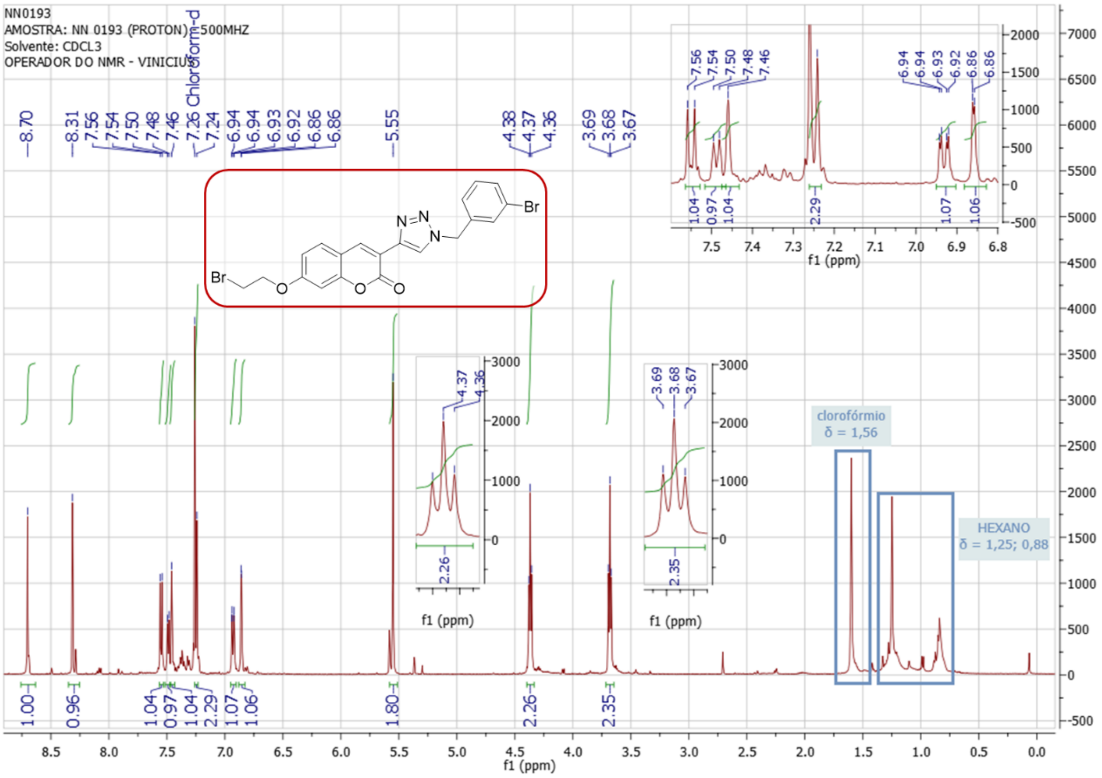

Figure S49. ^{13}C NMR spectra of **14l.**

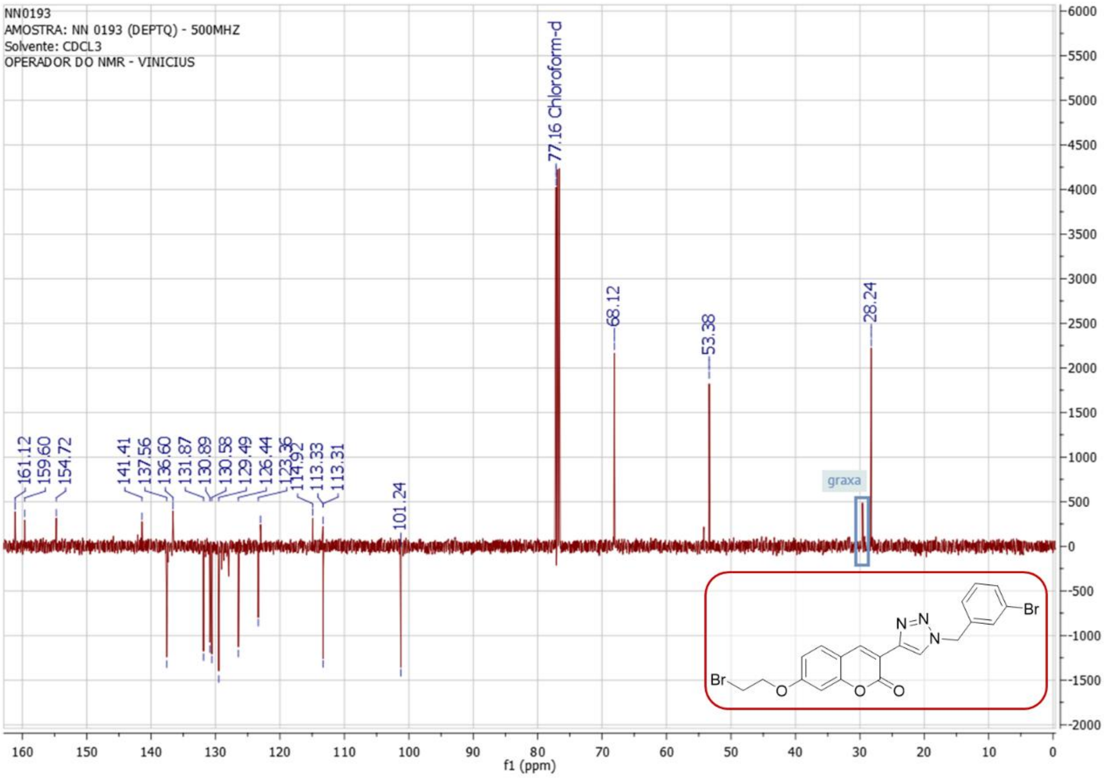

Figure S50. ^{1}H NMR spectra of **14m.**

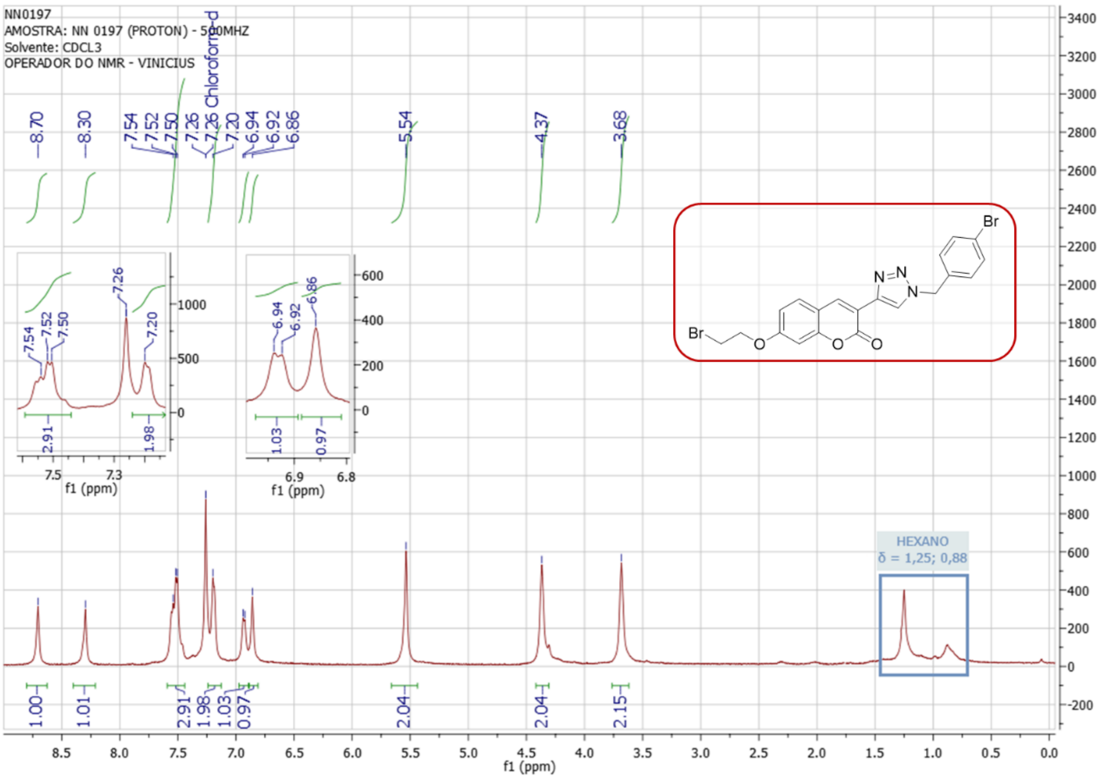

Figure S51. ^{13}C NMR spectra of **14m.**

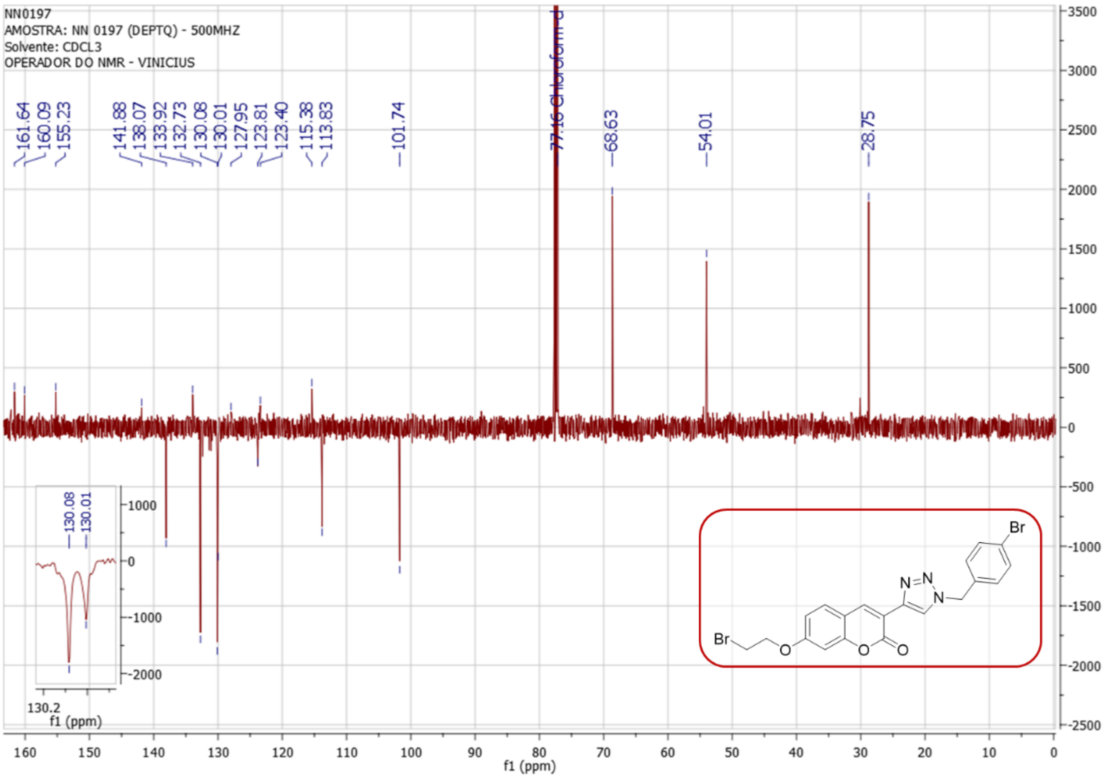

Figure S52. ^{1}H NMR spectra of **14n.**

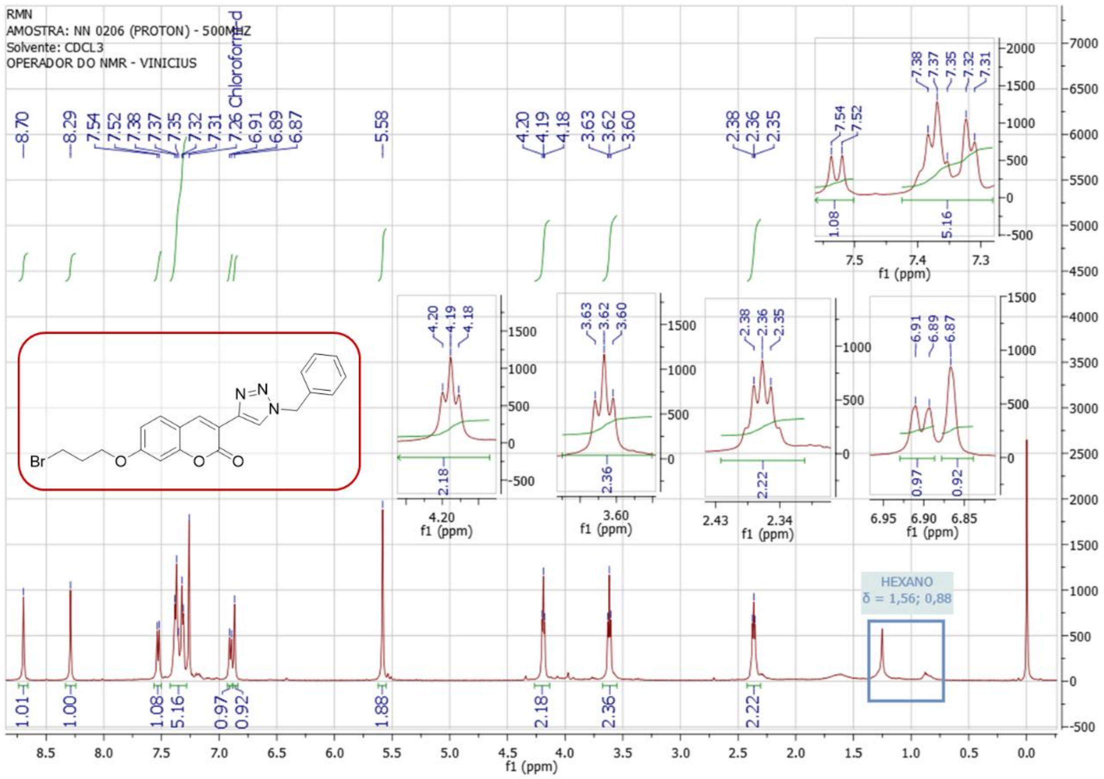

Figure S53. ^{13}C NMR spectra of **14n.**

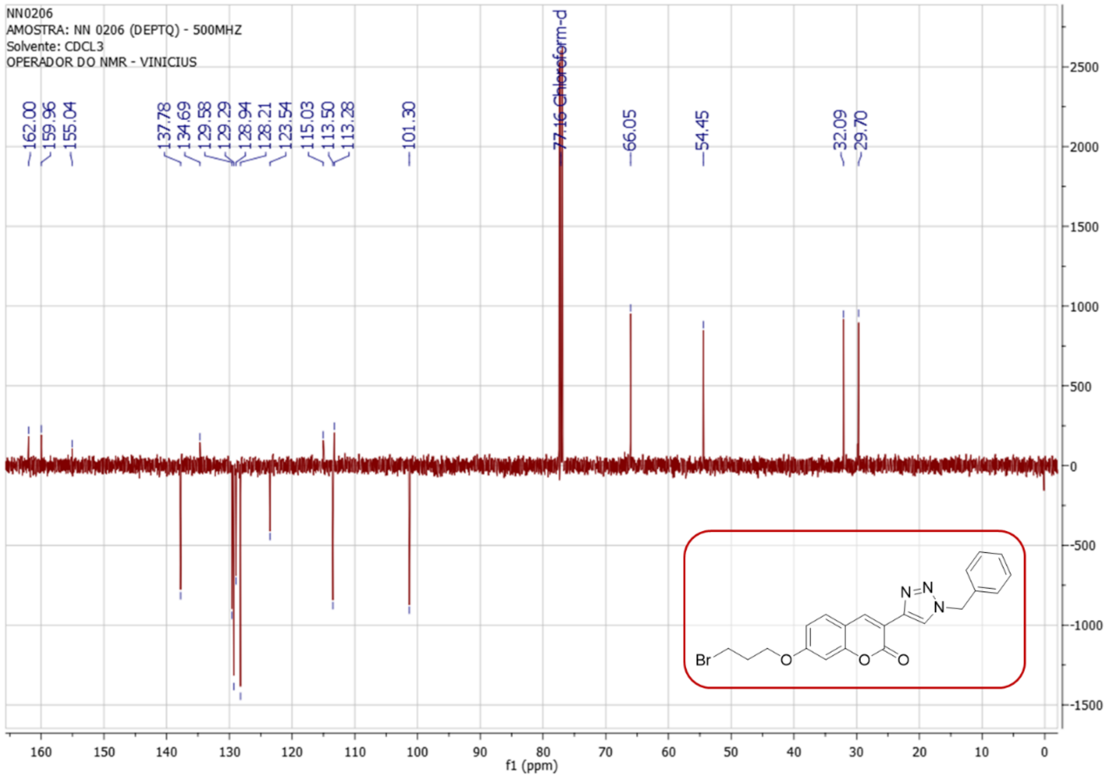

Figure S54. ^{1}H NMR spectra of **1a.**

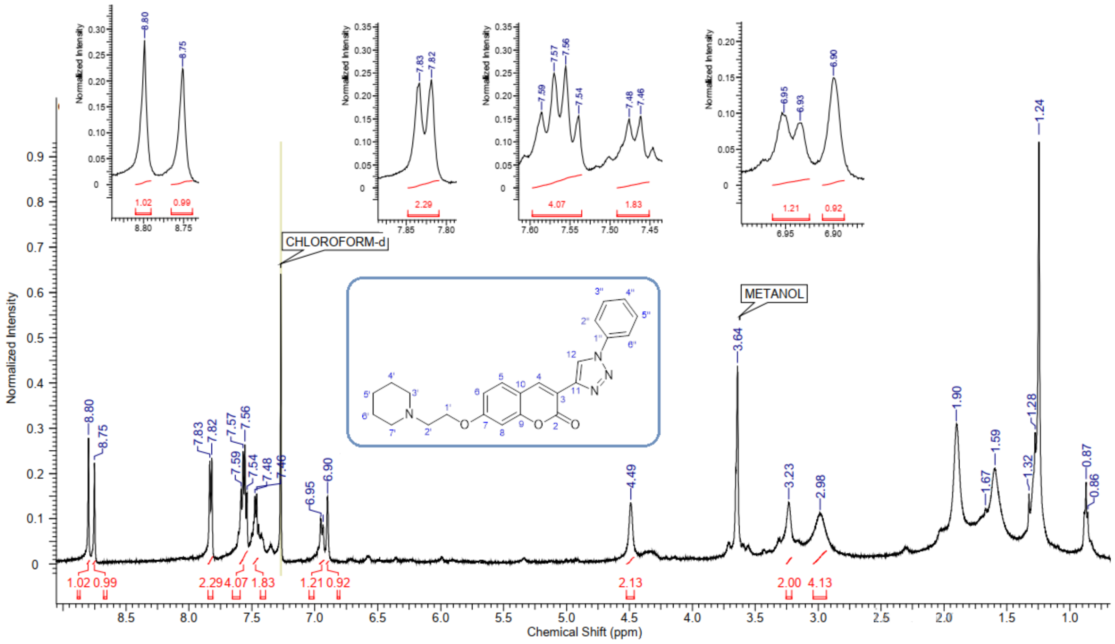

Figure S55. ^{13}C NMR spectra of **1a.**

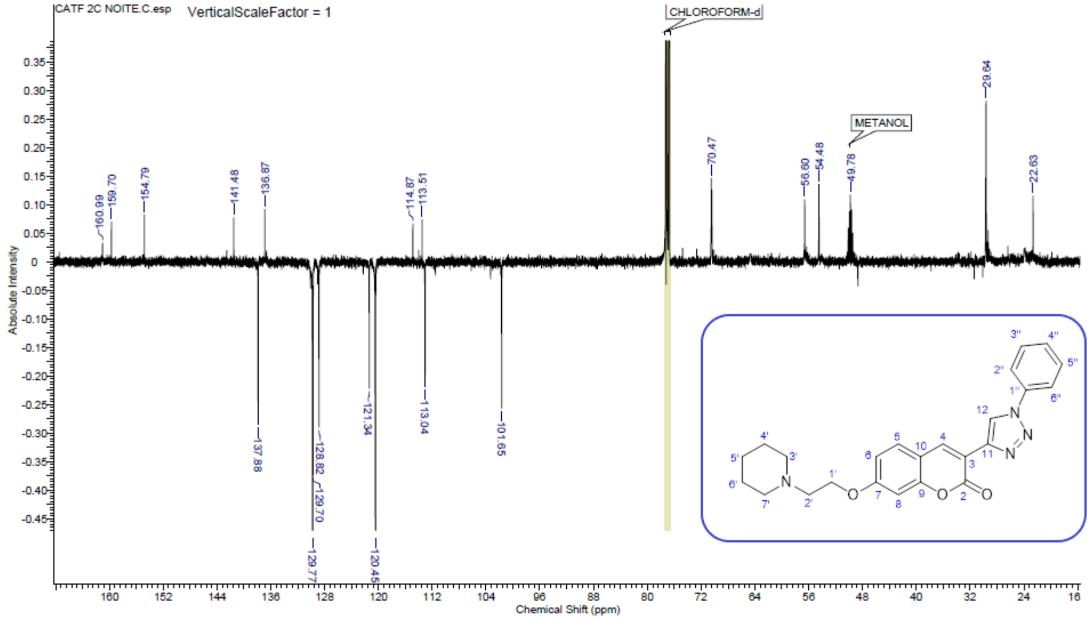

Figure S56. ^{1}H NMR spectra of **1b.**

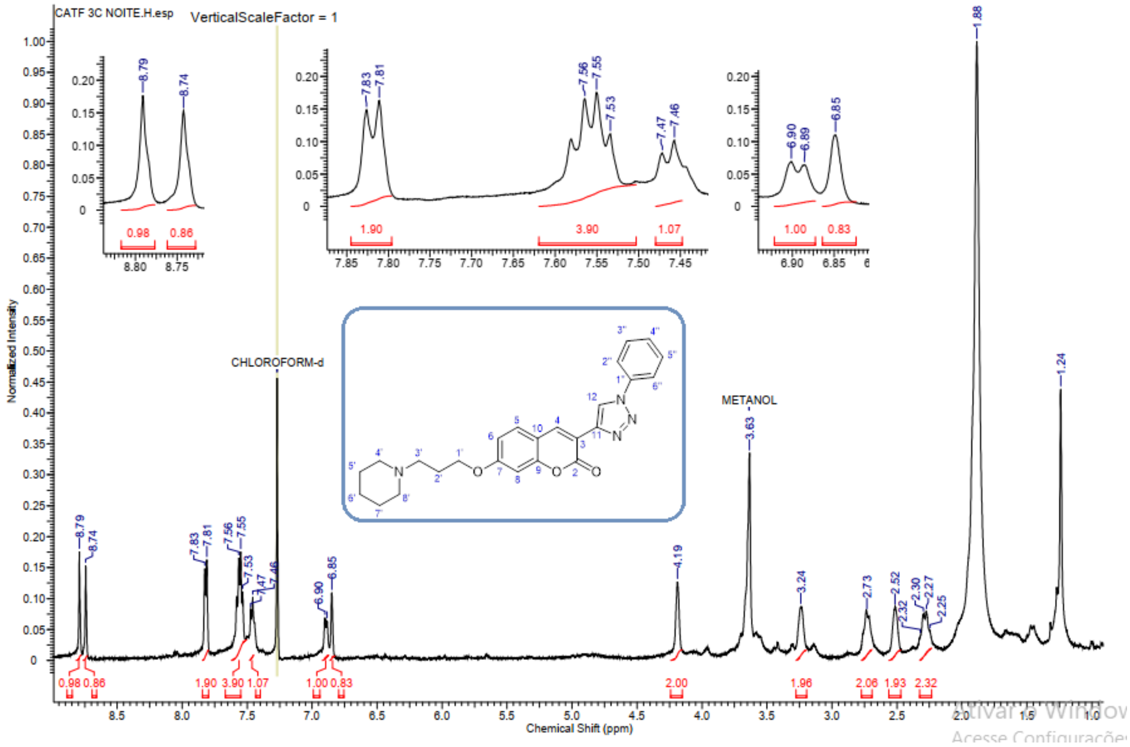

Figure S57. ^{13}C NMR spectra of **1b.**

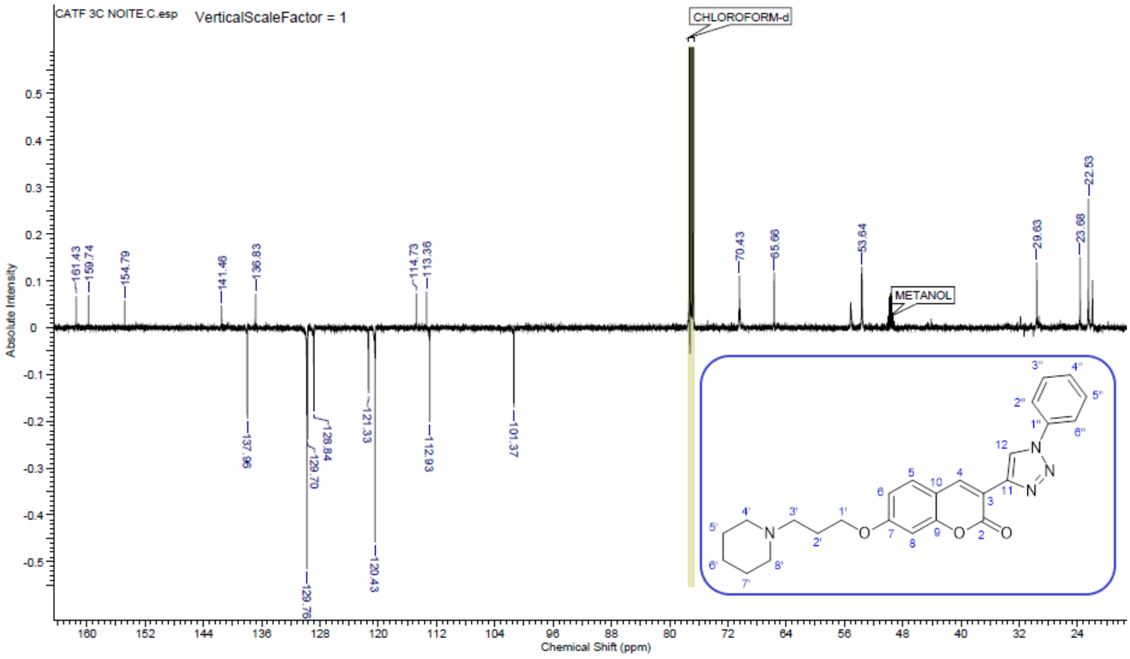

Figure S58. ^{1}H NMR spectra of **1c.**

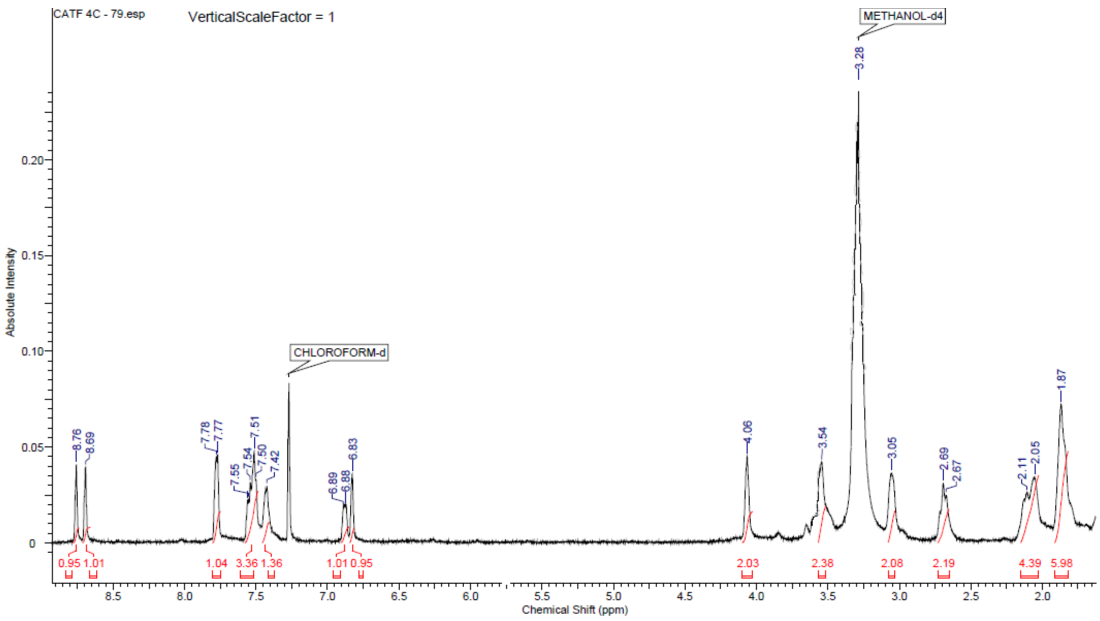

Figure S59. ^{13}C NMR spectra of **1c.**

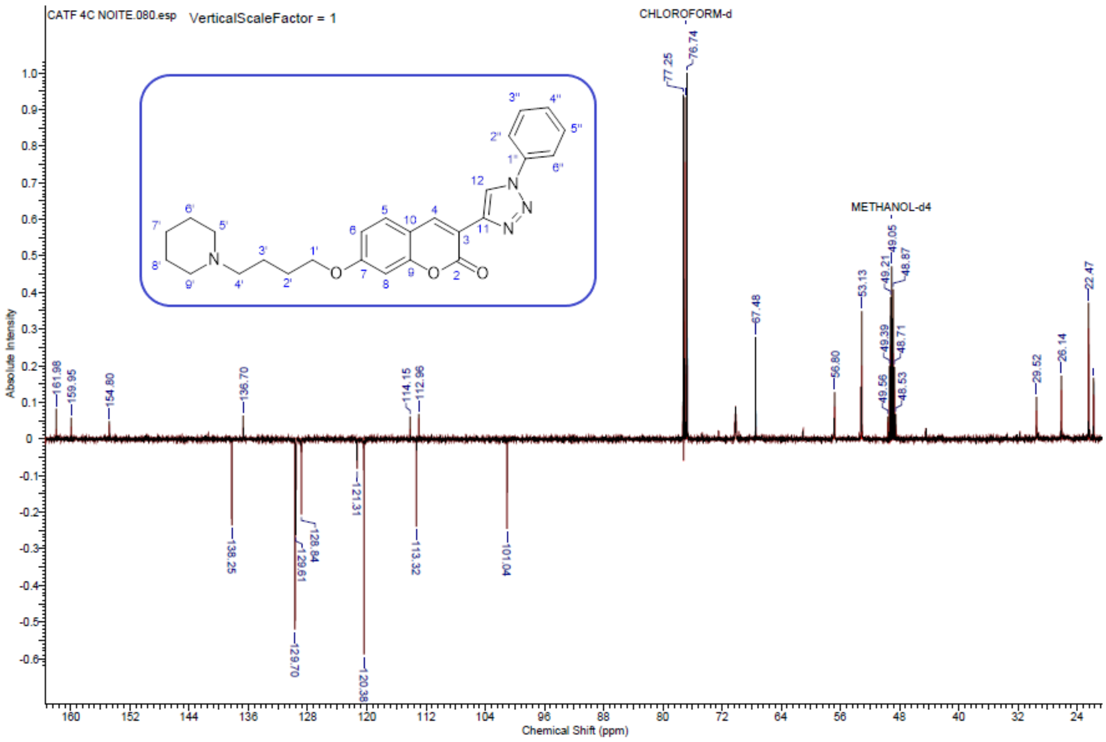

Figure S60. ^{1}H NMR spectra of **1d.**

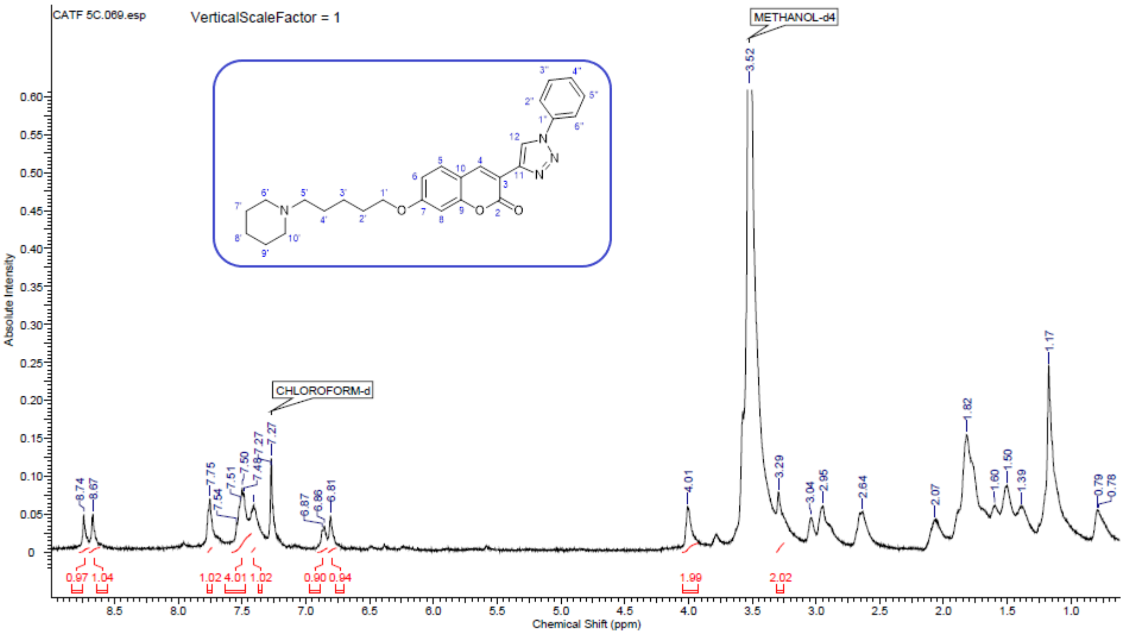

Figure S61. ^{13}C NMR spectra of **1d.**

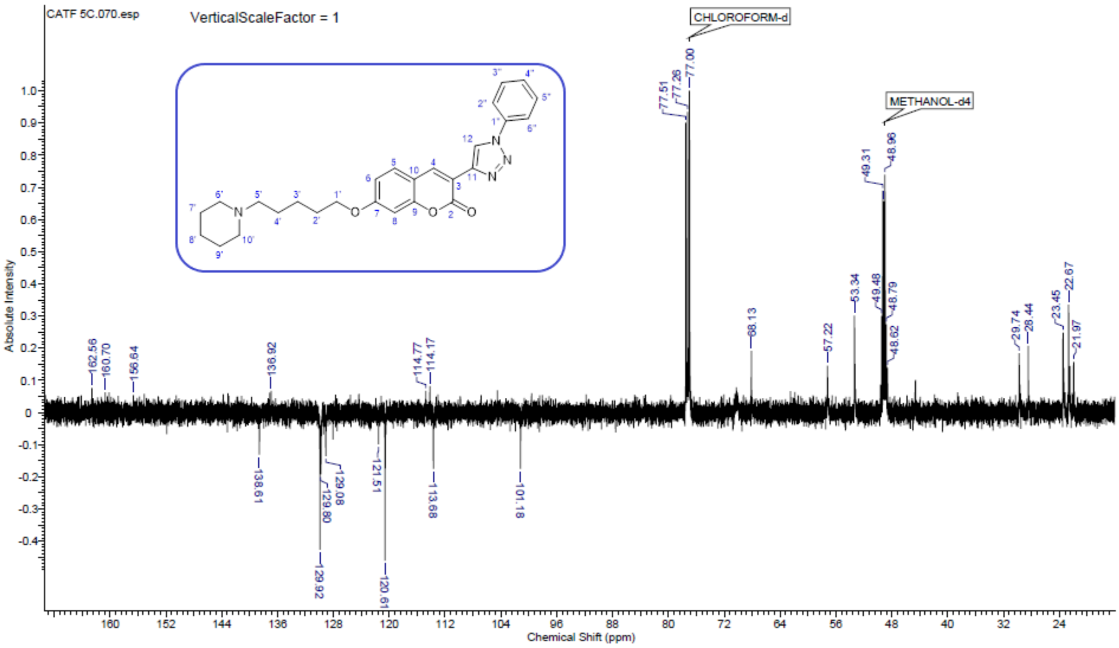

Figure S62. ^{1}H NMR spectra of **1e.**

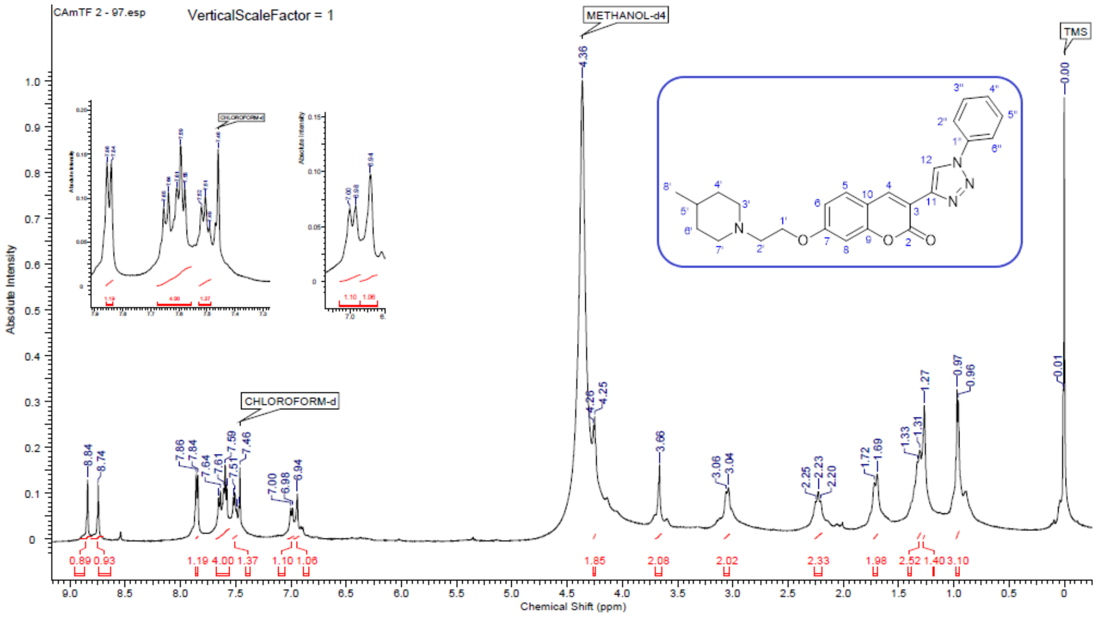

Figure S63. ^{13}C NMR spectra of **1e.**

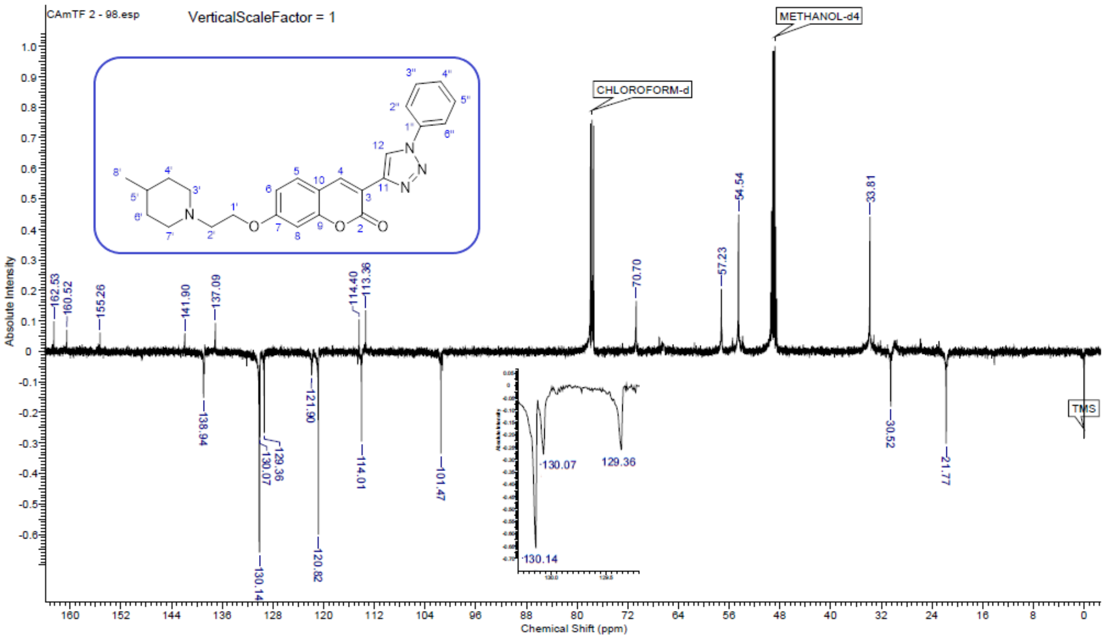

Figure S64. ^{1}H NMR spectra of **1f.**

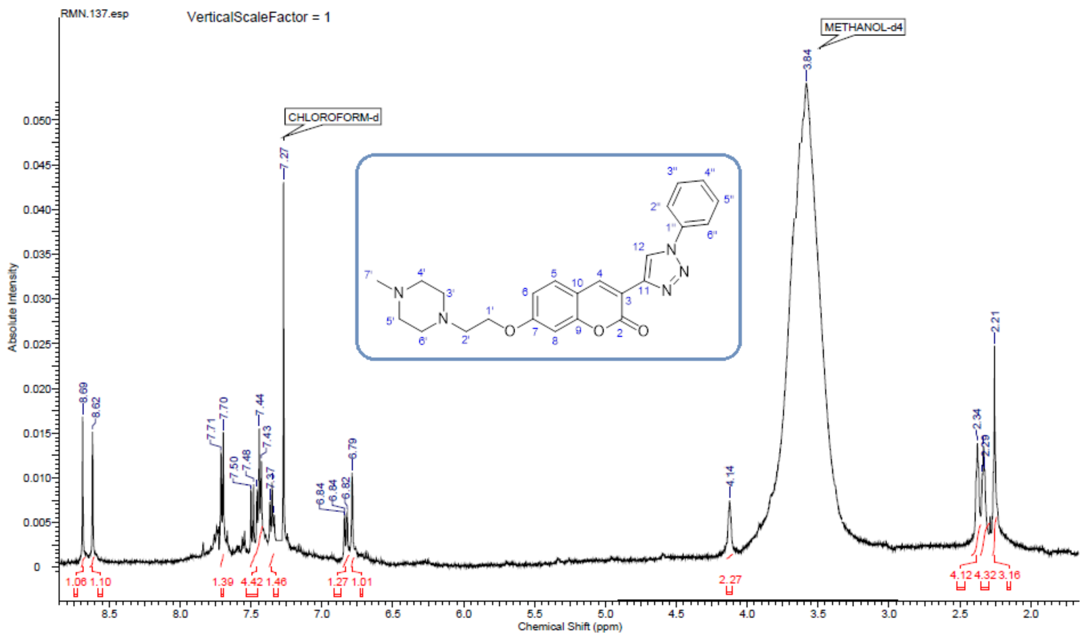

Figure S65. ^{13}C NMR spectra of **1f.**

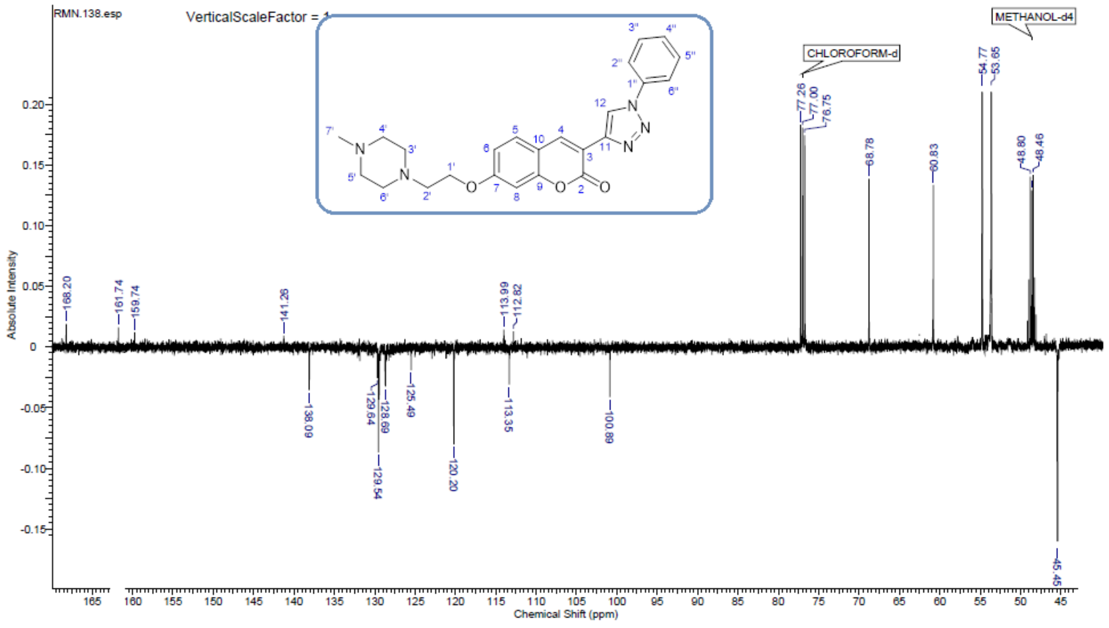

Figure S66. ^{1}H NMR spectra of **1g.**

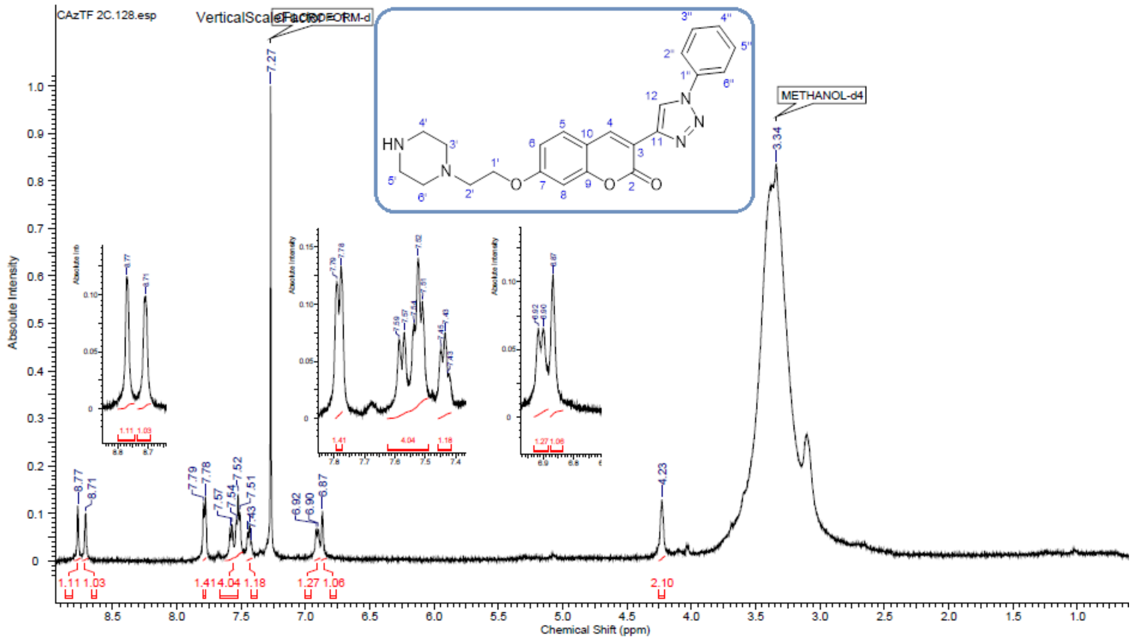

Figure S67. ^{13}C NMR spectra of **1g.**

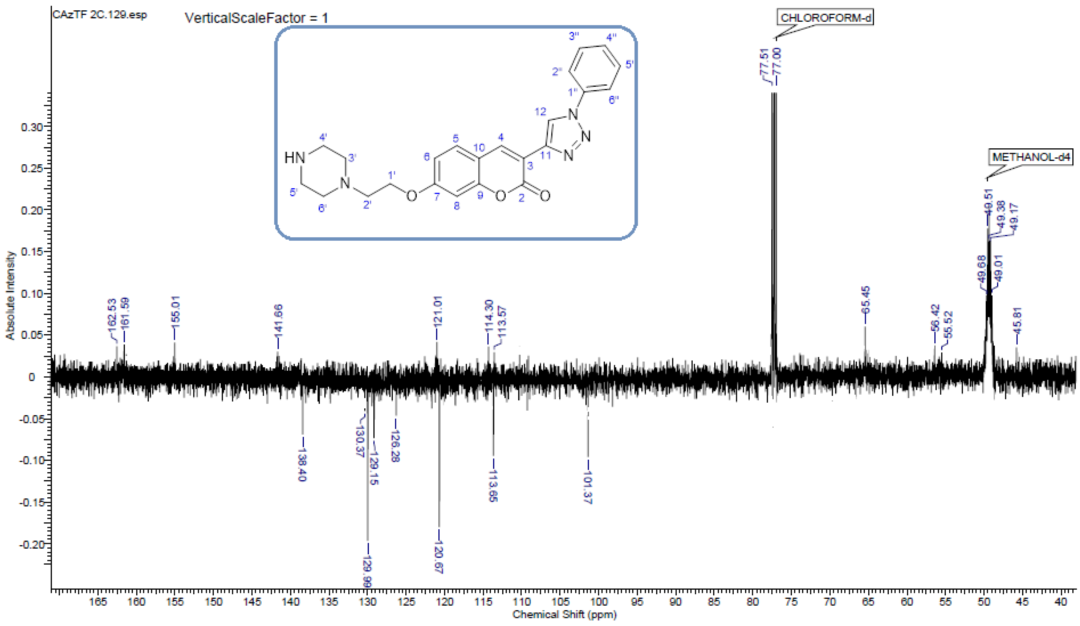

Figure S68. ^{1}H NMR spectra of **1h.**

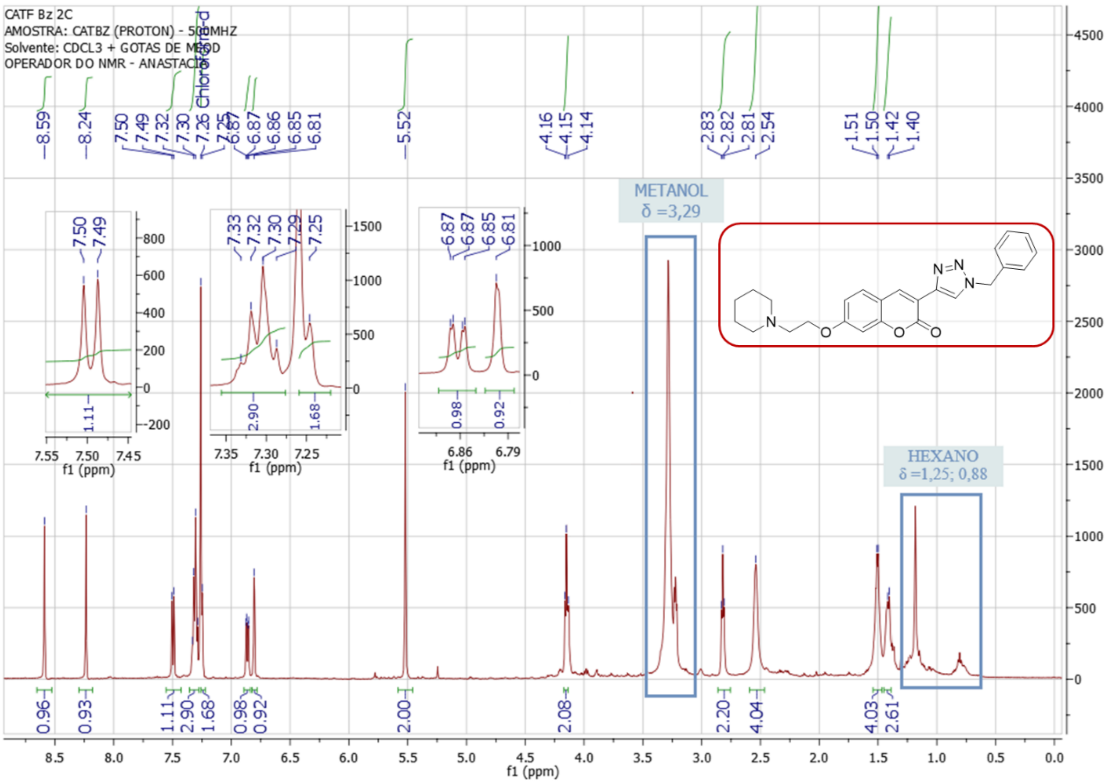

Figure S69. ^{13}C NMR spectra of **1h.**

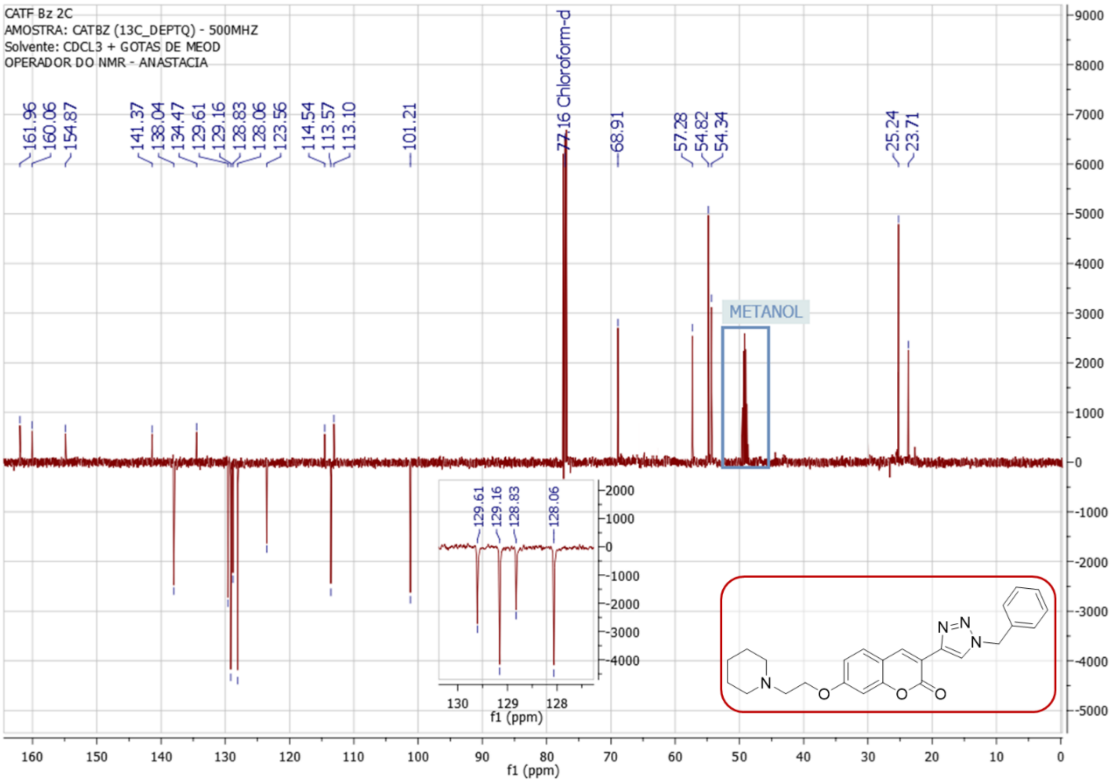

Figure S70. ^{1}H NMR spectra of **1i.**

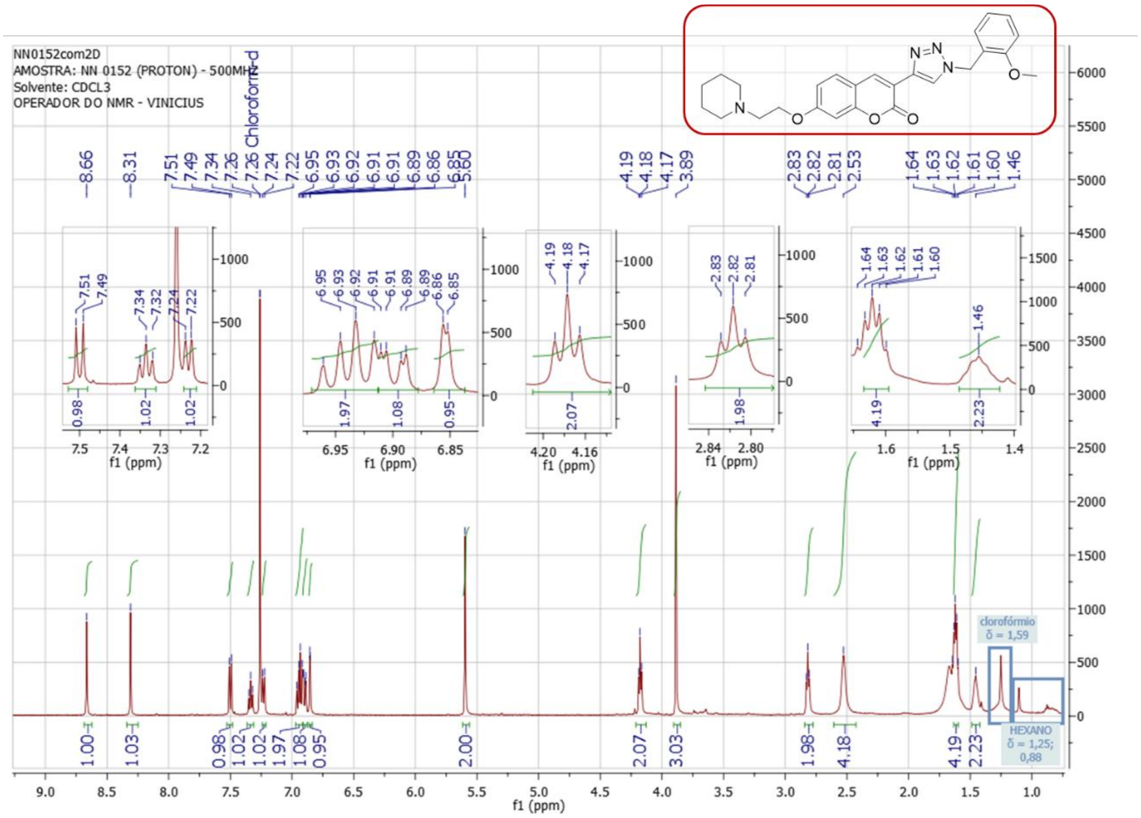

Figure S71. ^{13}C NMR spectra of **1i.**

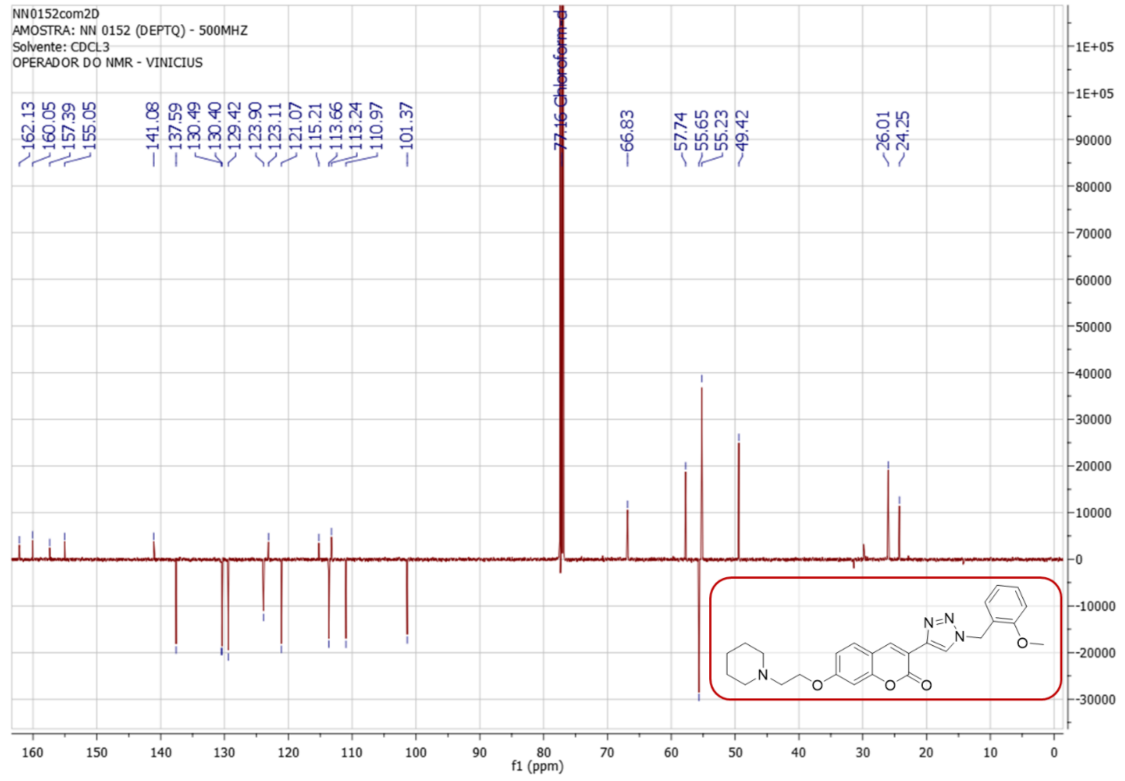

Figure S72. ^{1}H NMR spectra of **1j.**

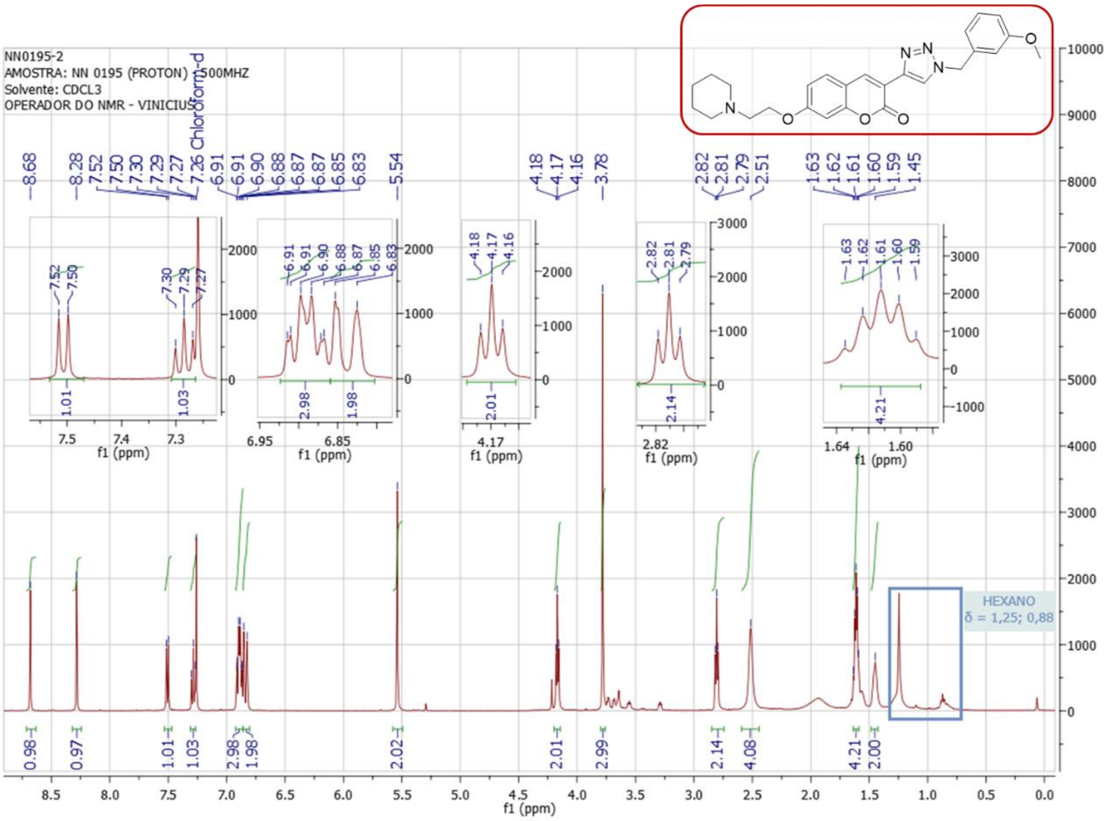

Figure S73. ^{13}C NMR spectra of **1j.**

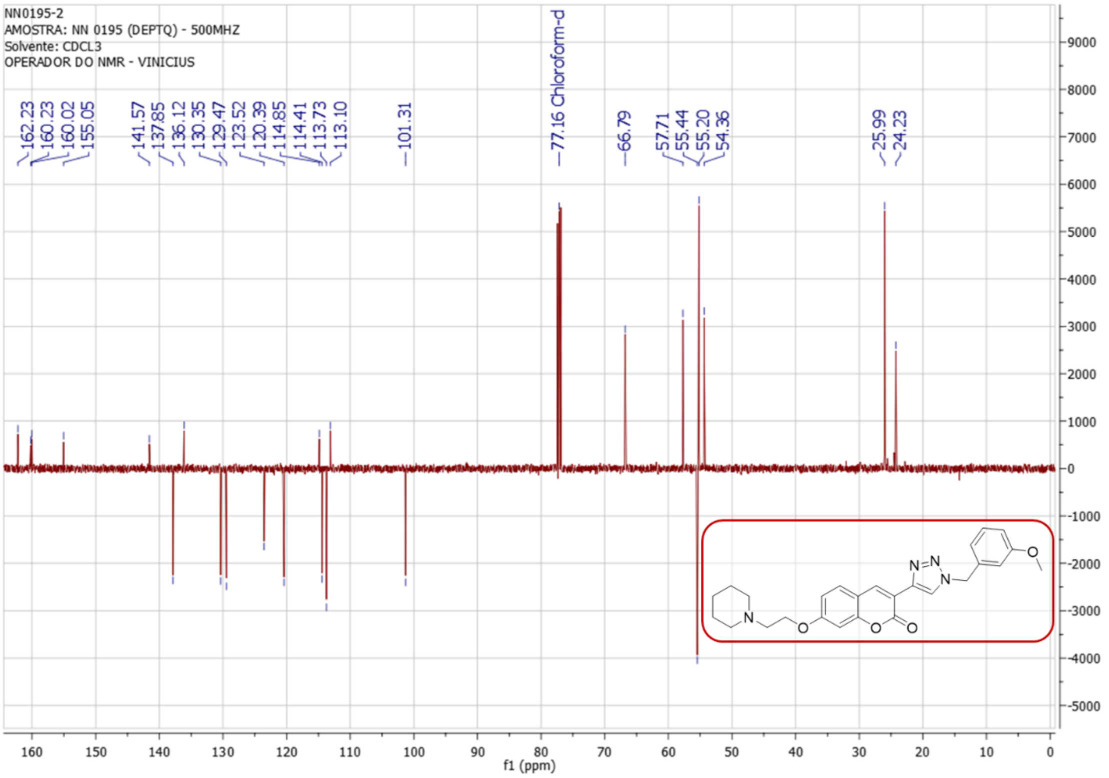

Figure S74. ^{1}H NMR spectra of **1k.**

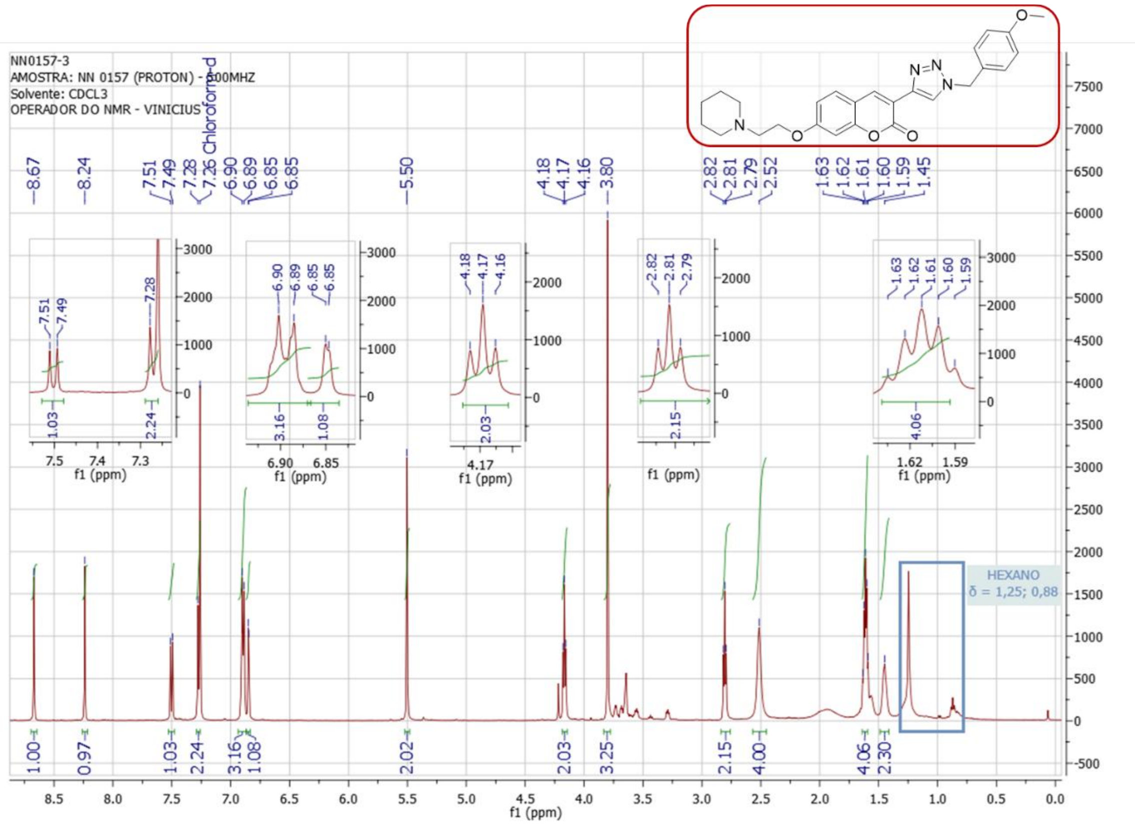

Figure S75. ^{13}C NMR spectra of **1k.**

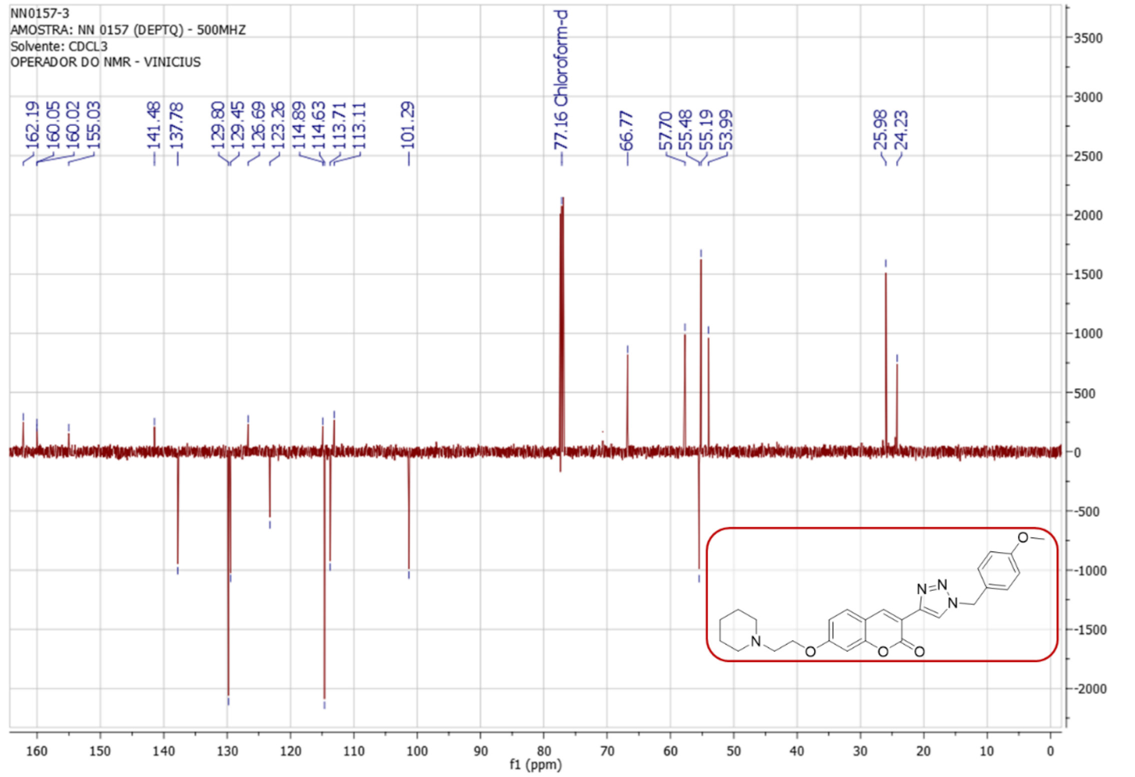

Figure S76. ^{1}H NMR spectra of **1l.**

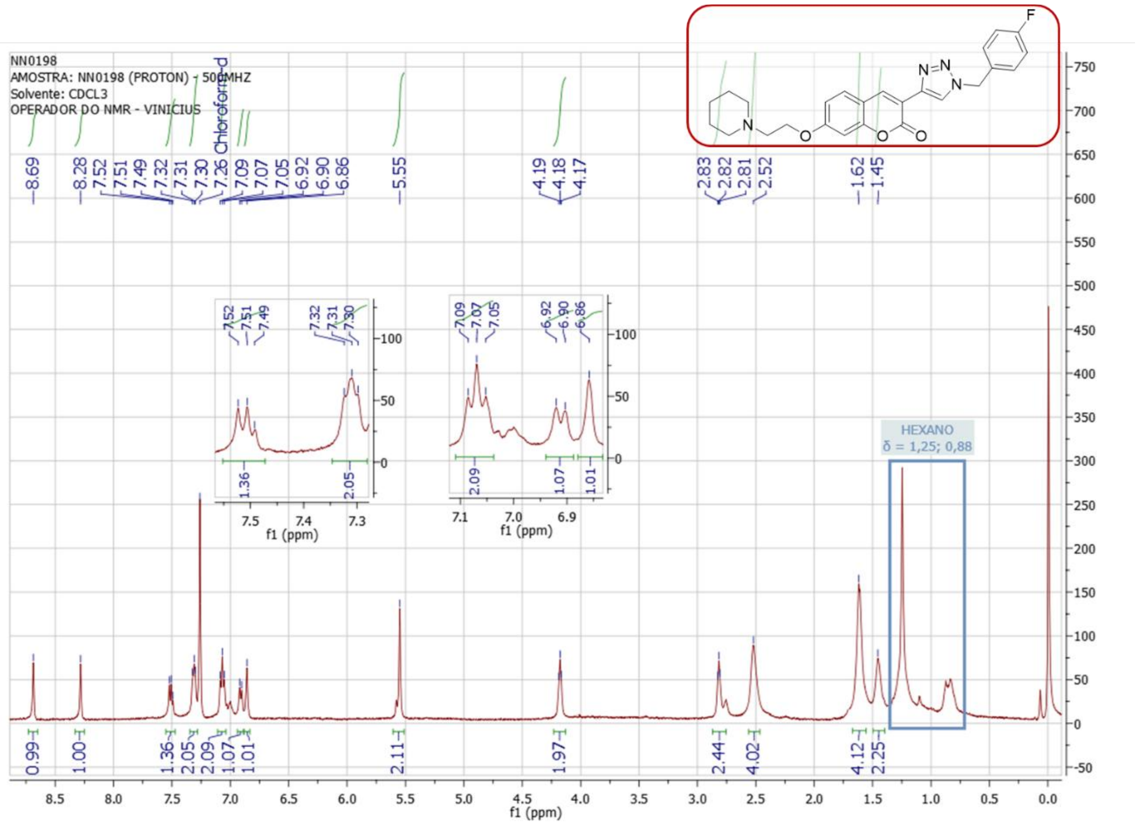

Figure S77. ^{13}C NMR spectra of **1l.**

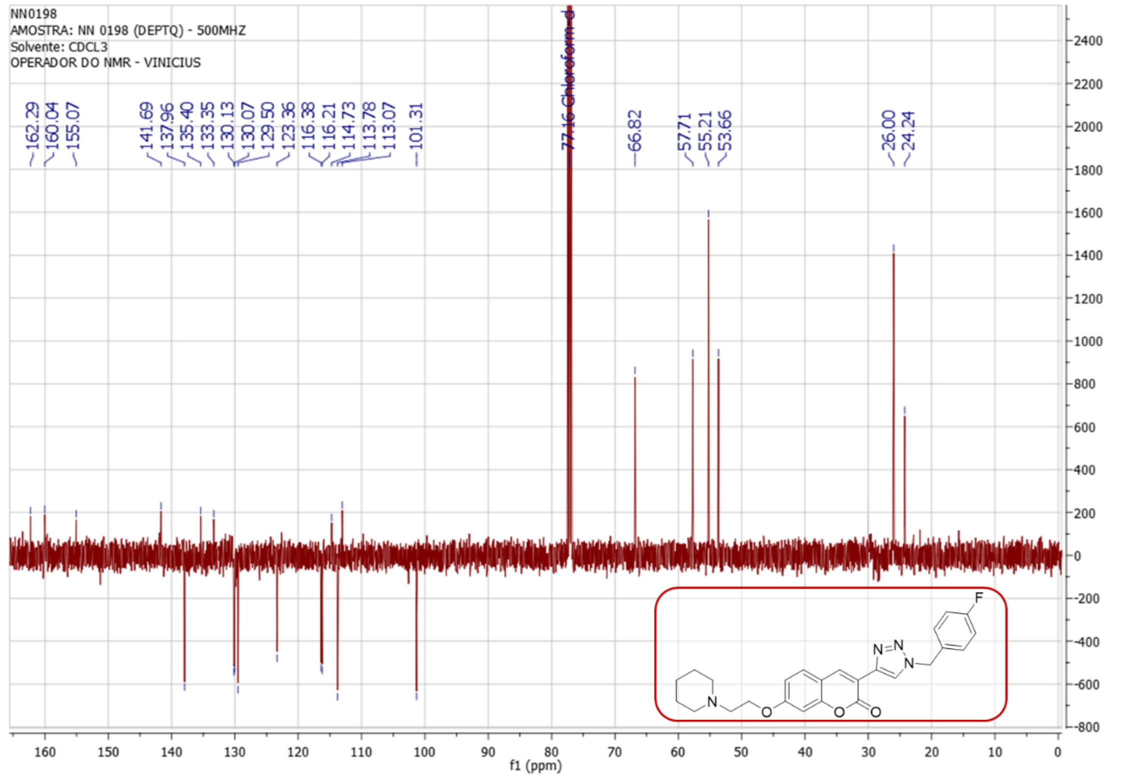

Figure S78. ^{1}H NMR spectra of **1m.**

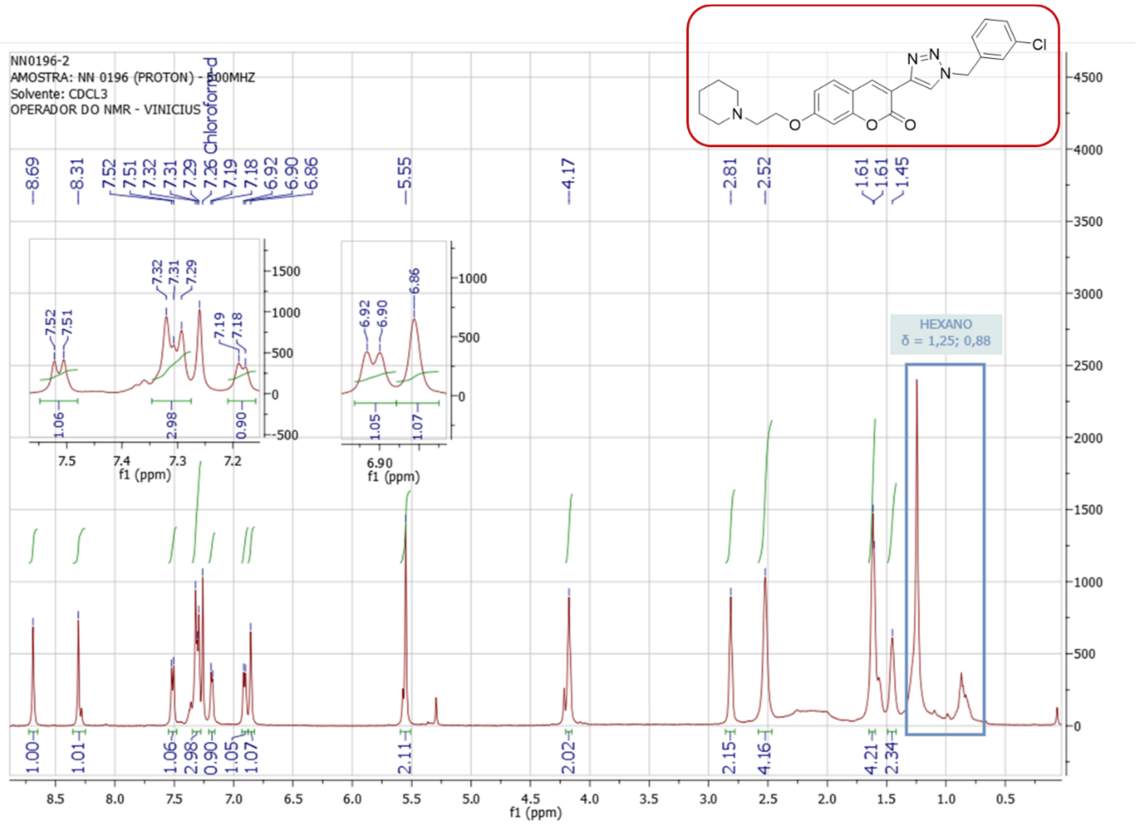

Figure S79. ^{13}C NMR spectra of **1m.**

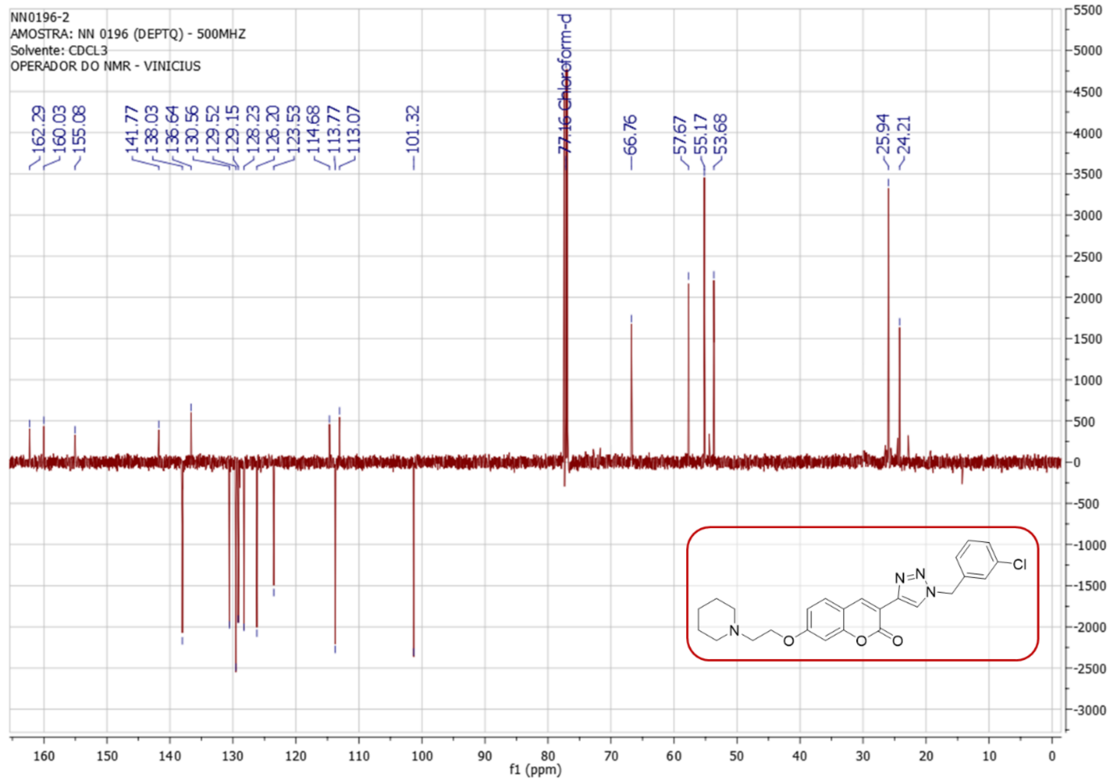

Figure S80. ^{1}H NMR spectra of **1n.**

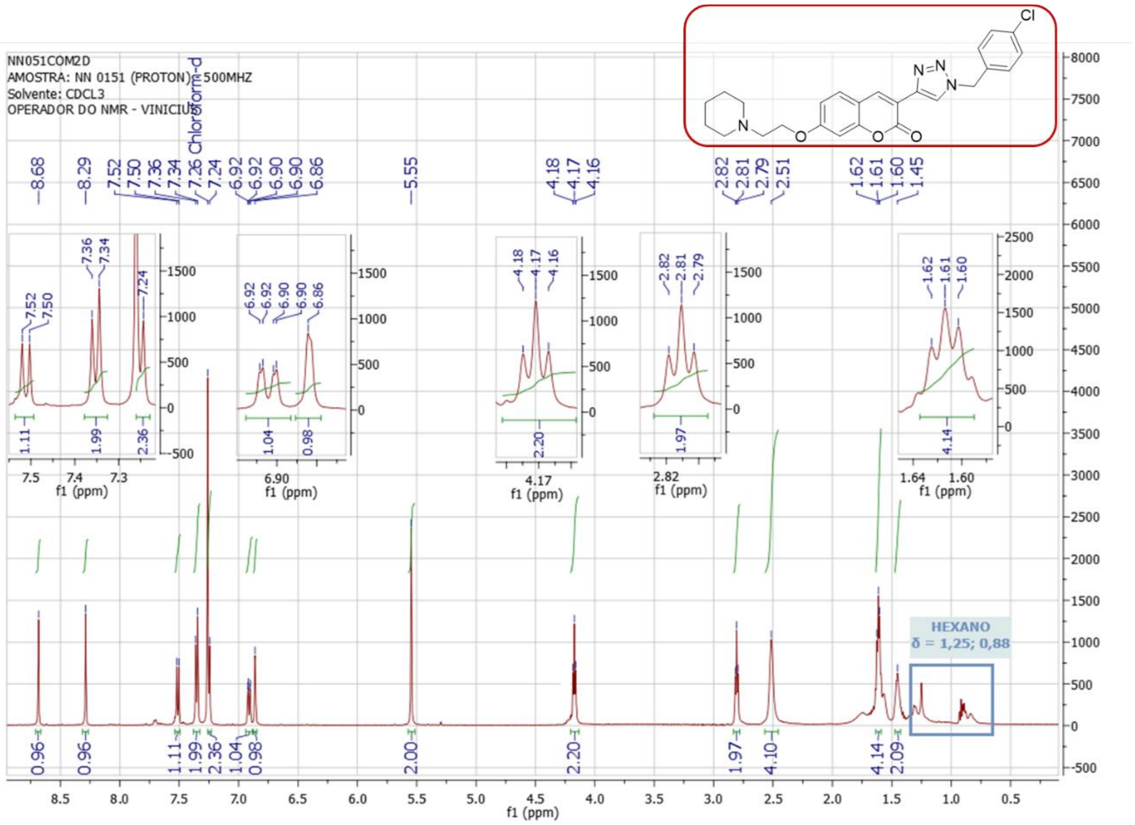

Figure S81. ^{13}C NMR spectra of **1n.**

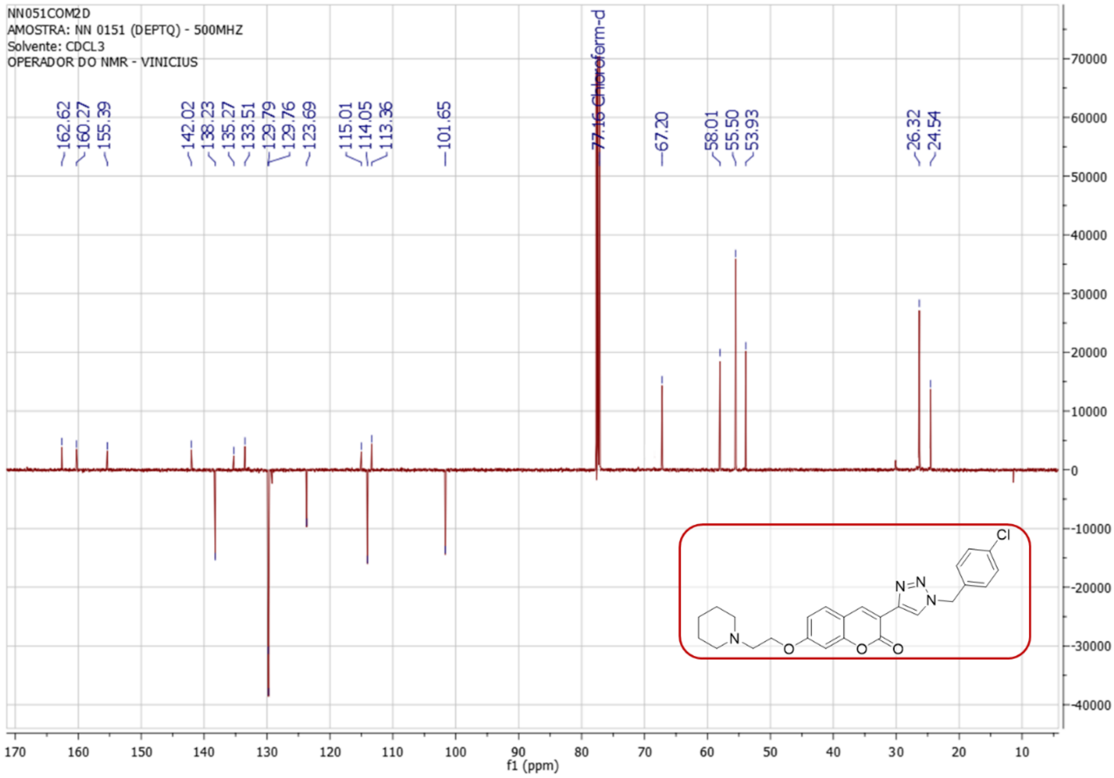

Figure S82. ^{1}H NMR spectra of **1o.**

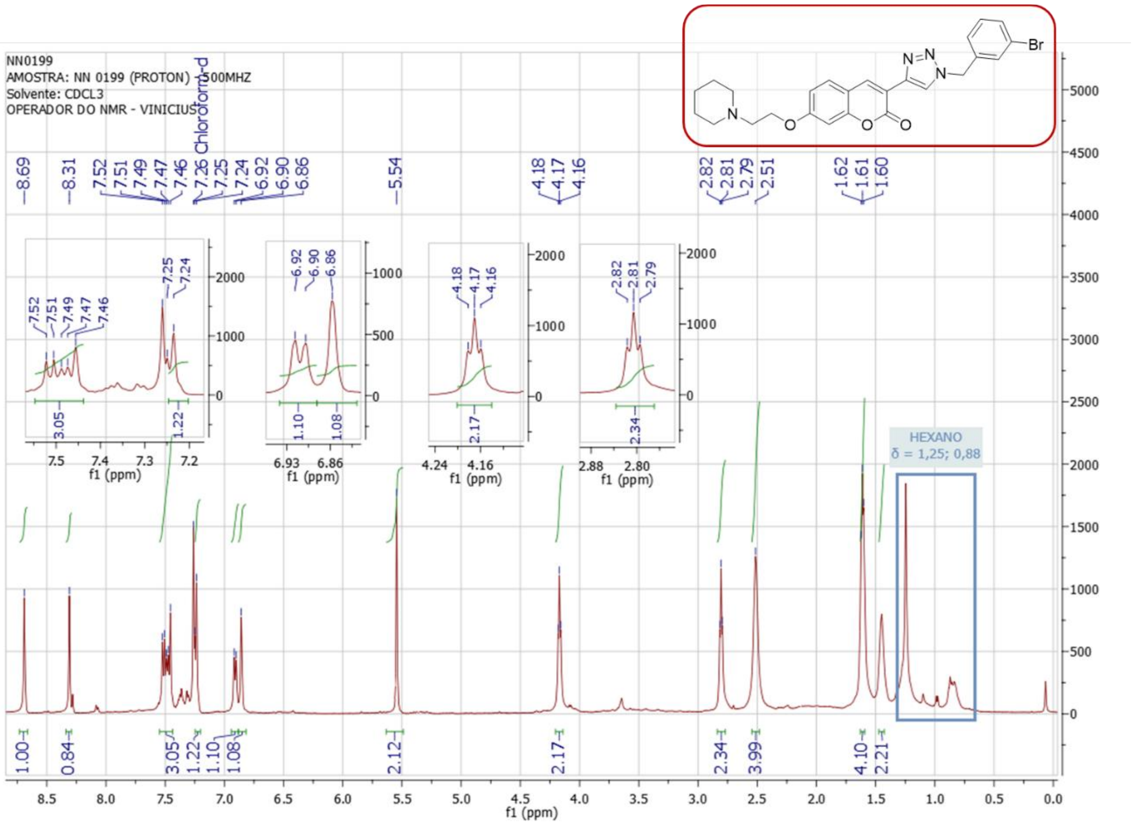

Figure S83. ^{13}C NMR spectra of **1o.**

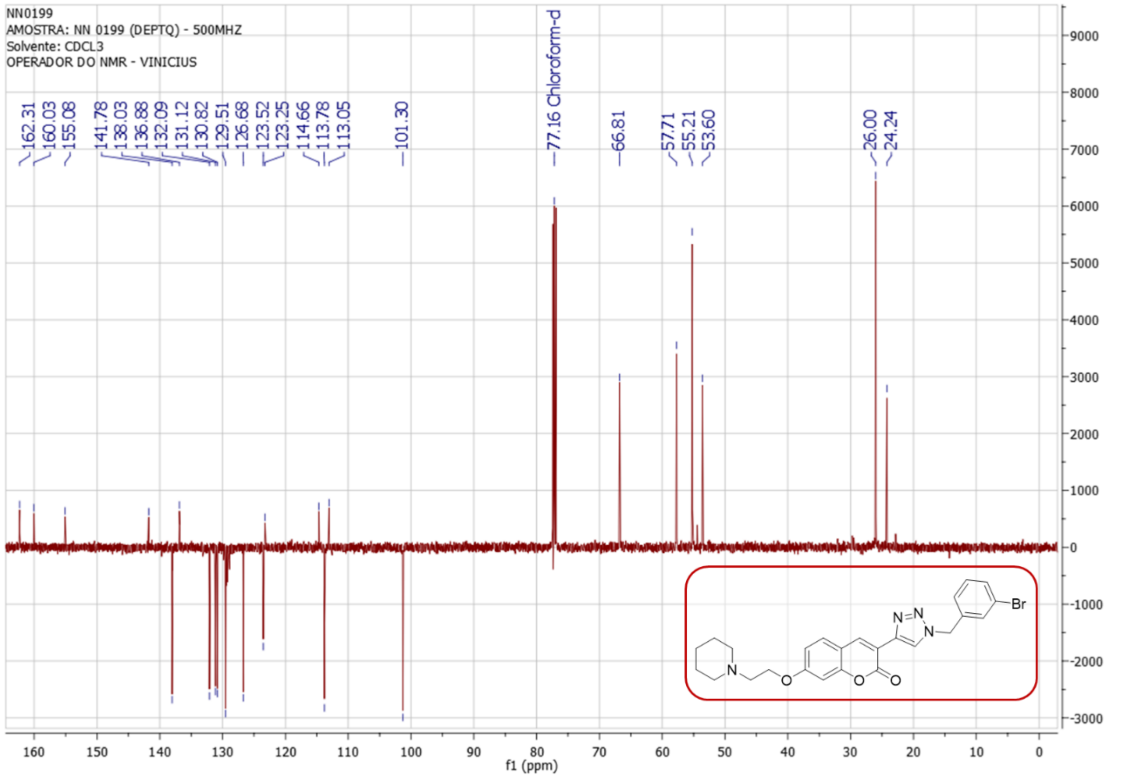

Figure S84. ^{1}H NMR spectra of **1p.**

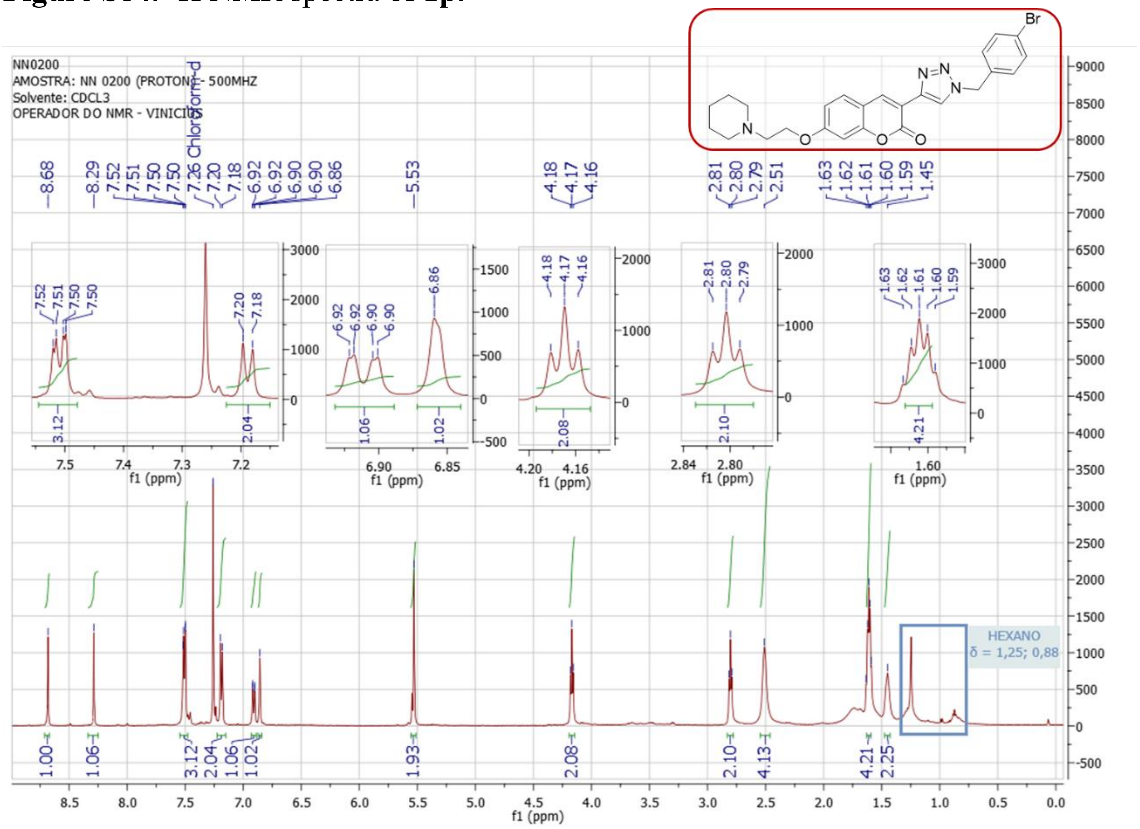

Figure S85. ^{13}C NMR spectra of **1p.**

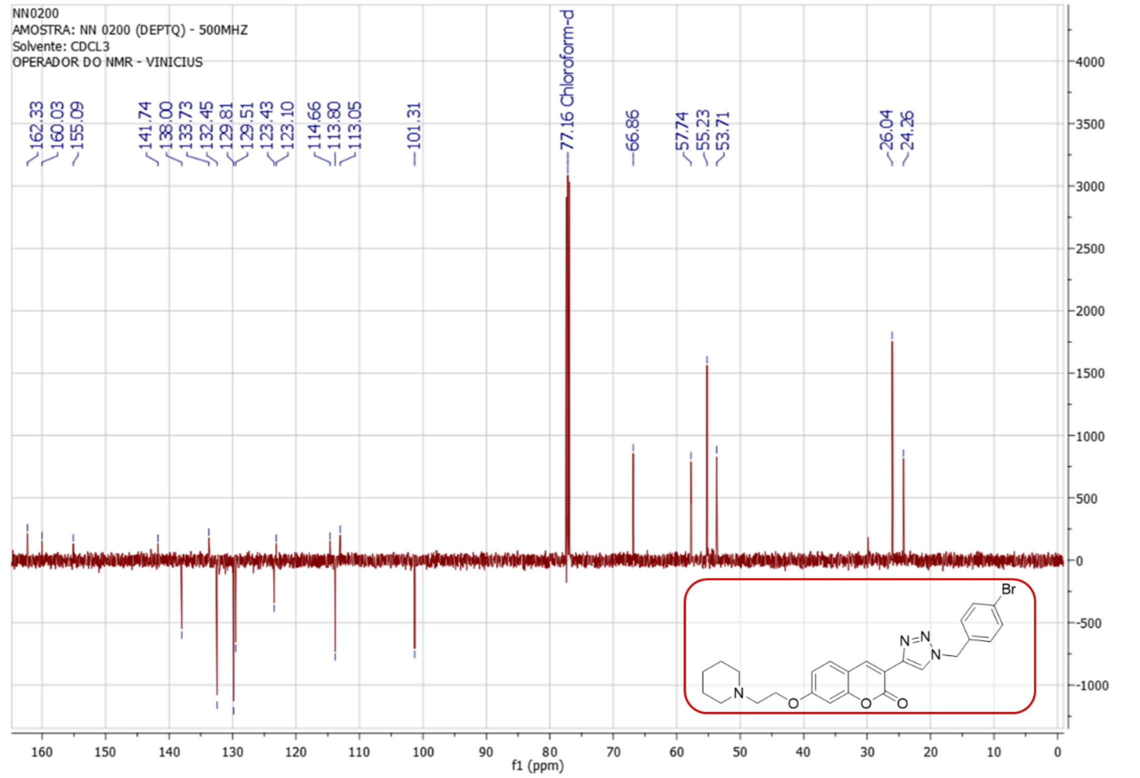

Figure S86. ^{1}H NMR spectra of **1q.**

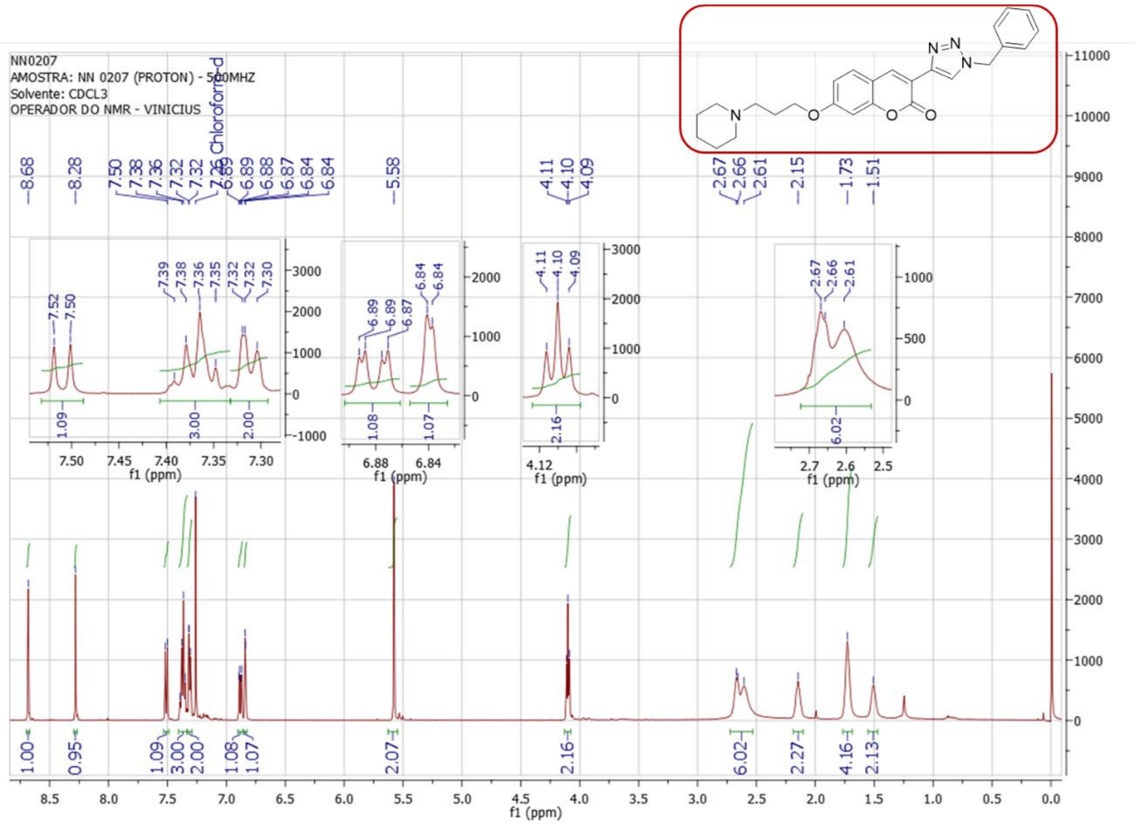

Figure S87. ^{13}C NMR spectra of **1q.**

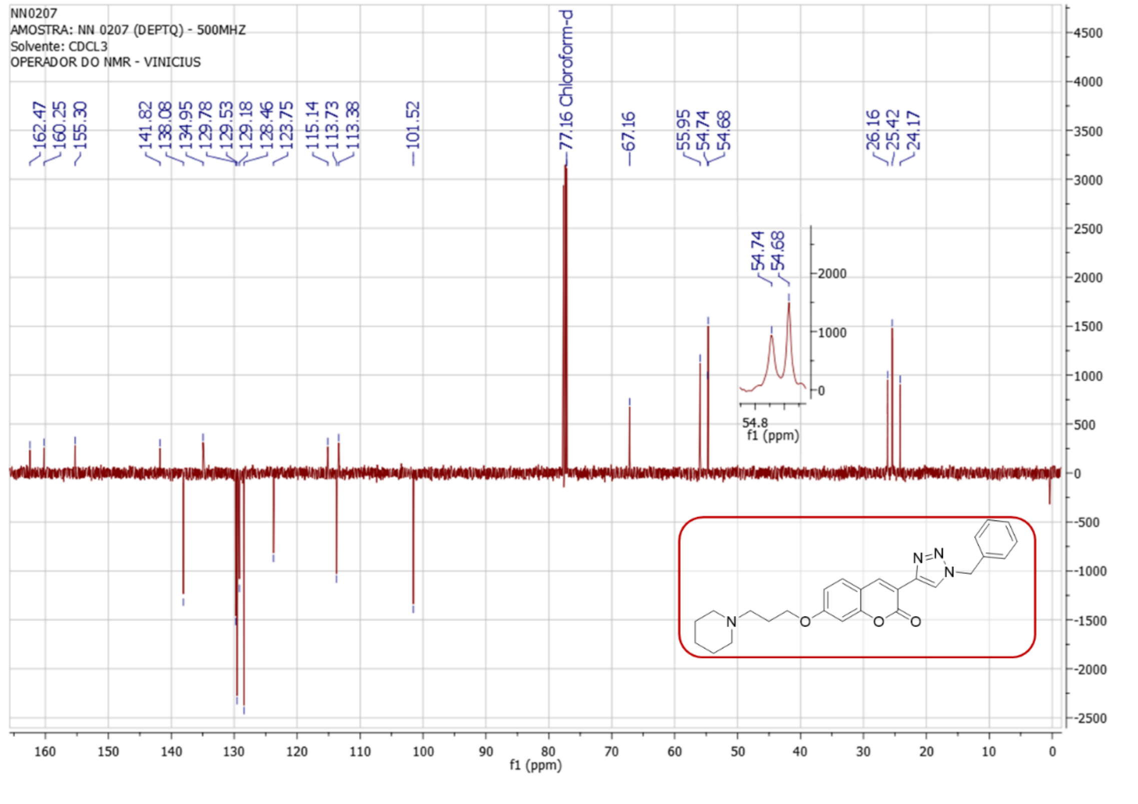

HPLC data

Figure S88. HPLC data for compound **1a.**

HPLC Report

LaDMol·QM

Agilent Technologies

**Data file:**	C:\CHEM32\1\DATA\LUCAS 2024-06-05 13-25-41\001-0101.D		
**Sample name:**	Ph		
**Location:**	Vial 1	**Injection date:**	6/5/2024 1:27:06 PM
**Injection:**	1 of 1	**Injection volume:**	10.000
**Last changed:**	6/5/2024 1:25:07 PM		

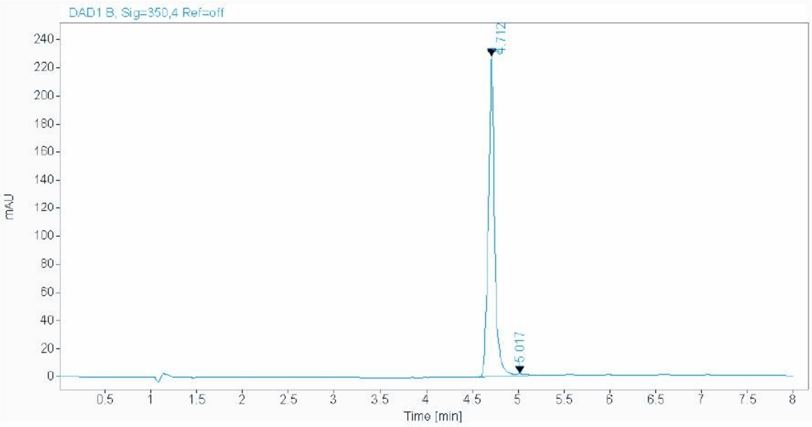

Signal: DAD1 B, Sig=350,4 Ref=off

RT [min]	Type	Width [min]	Area	Height	Area%	Name
4.712	BV	0.0693	1085.0249	228.8973	98.7841	
5.017	VB	0.1066	13.3548	1.6475	1.2159	

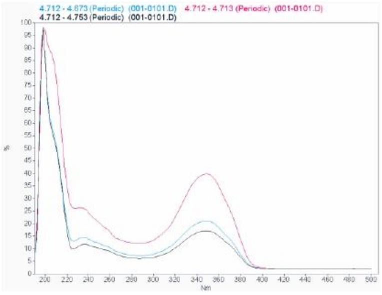

Figure S89. HPLC data for compound **1b.**

HPLC Report

LaDMol·QM

Agilent Technologies

**Data file:**	C:\CHEM32\1\DATA\LUCAS 2024-06-05 14-33-44\011-0101.D		
**Sample name:**	3C-Ph		6/5/2024 2:35:10 PM
**Location:**	Vial 11	**Injection date:**	
**Injection:**	1 of 1	**Injection volume:**	10.000
**Last changed:**	6/5/2024 2:31:59 PM		

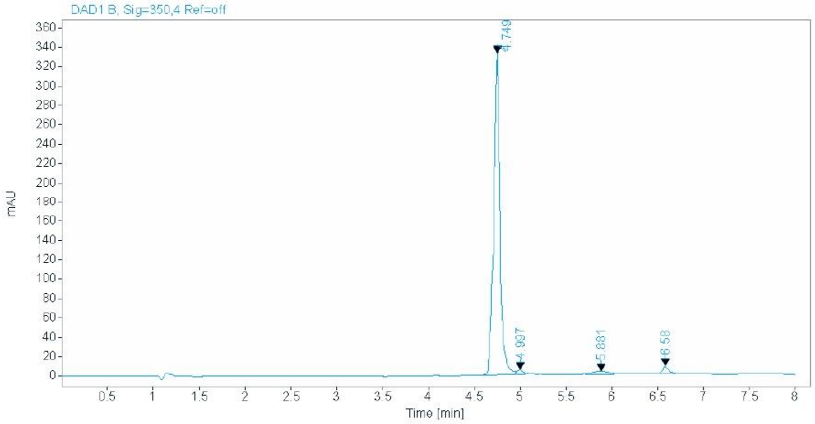

Signal: DAD1 B, Sig=350,4 Ref=off

RT [min]	Type	Width [min]	Area	Height	Area%	Name
4.749	BV	0.0668	1566.8447	333.7283	95.5936	
4.997	MF	0.0629	16.9631	4.4954	1.0241	
5.881	FM	0.1952	35.3970	3.0222	1.6370	
6.580	VV	0.0722	37.1914	7.7175	1.7453	

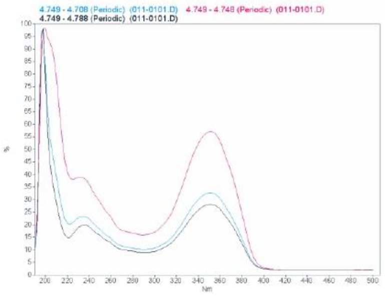

Figure S90. HPLC data for compound **1c.**

HPLC Report

LaDMol·QM

Agilent Technologies

**Data file:**	C:\CHEM32\1\DATA\LUCAS 2024-06-05 14-33-44\012-0201.D		
**Sample name:**	4C-Ph		6/5/2024 2:44:37 PM
**Location:**	Vial 12	**Injection date:**	
**Injection:**	1 of 1	**Injection volume:**	10.000
**Last changed:**	6/5/2024 2:31:59 PM		

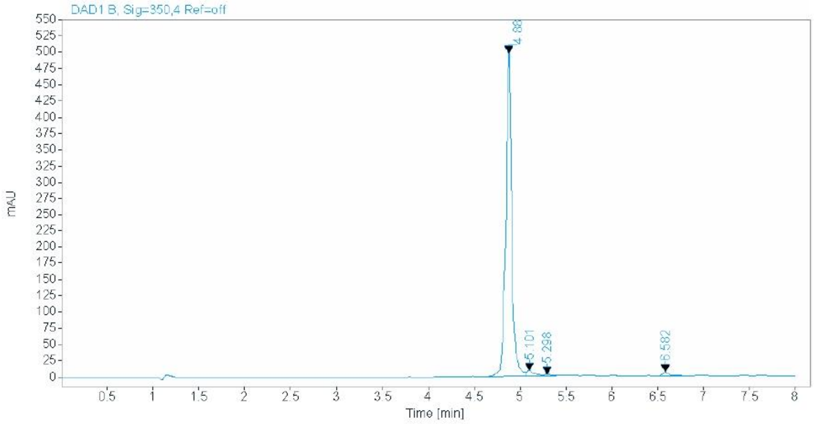

Signal: DAD1 B, Sig=350,4 Ref=off

RT [min]	Type	Width [min]	Area	Height	Area%	Name
4.880	BV	0.0668	2267.4912	501.2003	96.0746	
5.101	VV	0.0846	51.8030	8.5606	2.1949	
5.298	VB	0.0888	16.2035	2.4603	0.6865	
6.582	BB	0.0718	24.6379	5.1516	1.0439	

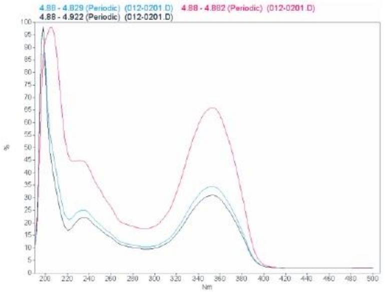

Figure S91. HPLC data for compound **1d.**

HPLC Report

LaDMol·QM | Agilent Technologies

**Data file:**	C:\CHEM32\1\DATA\LUCAS 2024-06-06 13-45-43\008-0801.D		
**Sample name:**	5C-Ph		6/6/2024 1:45:43 PM
**Location:**	Vial 8	**Injection date:**	
**Injection:**	1 of 1	**Injection volume:**	10.000
**Last changed:**	6/6/2024 1:31:59 PM		

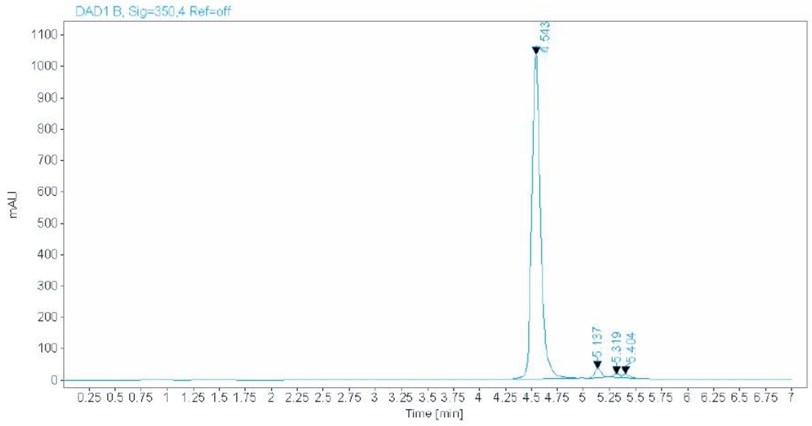

Signal: DAD1 B, Sig=350,4 Ref=off

RT [min]	Type	Width [min]	Area	Height	Area%	Name
4.543	VV	0.0905	6090.0220	1036.9198	95.2056	
5.137	BB	0.0711	130.0835	28.5894	3.4571	
5.319	BV	0.0646	51.2063	11.8037	0.7098	
5.404	VB	0.0685	52.3305	11.6280	0.6275	

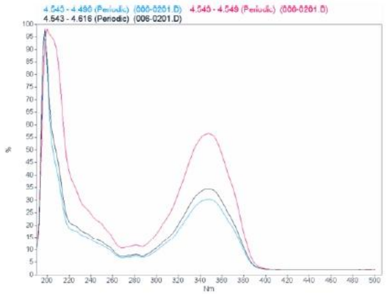

Figure S92. HPLC data for compound **1e.**

HPLC Report

LaDMol·QM

Agilent Technologies

**Data file:**	C:\CHEM32\1\DATA\LUCAS 2024-06-06 14-12-25\012-0601.D		
**Sample name:**	MePip-Ph		6/6/2024 2:12:11 PM
**Location:**	Vial 12	**Injection date:**	
**Injection:**	1 of 1	**Injection volume:**	10.000
**Last changed:**	6/6/2024 1:31:59 PM		

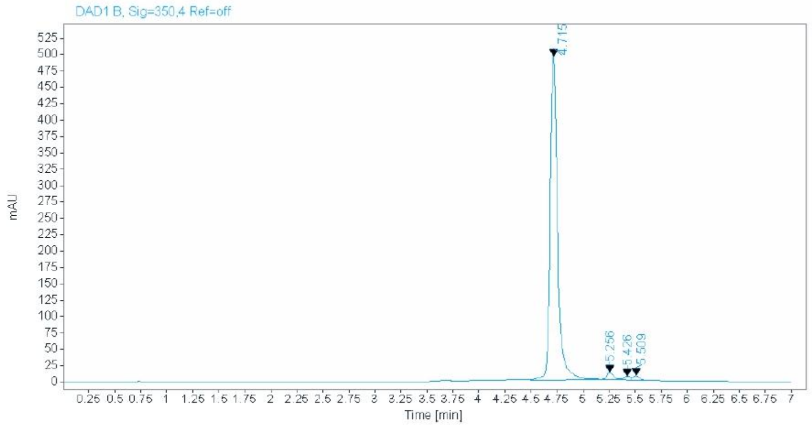

Signal: DAD1 B, Sig=350,4 Ref=off

RT [min]	Type	Width [min]	Area	Height	Area%	Name
4.715	VB	0.0731	2428.9050	496.2606	95.7196	
5.256	BV	0.0673	48.1598	10.9476	2.1039	
5.426	VV	0.0646	25.0995	5.7916	0.9922	
5.509	VB	0.0656	27.4295	6.2021	1.1843	

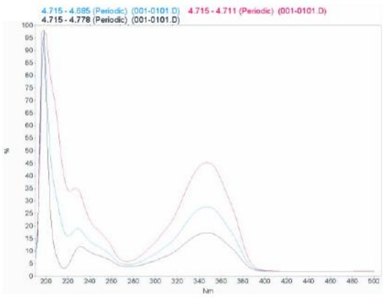

Figure S93. HPLC data for compound **1f.**

HPLC Report

LaDMol·QM

Agilent Technologies

**Data file:**	C:\CHEM32\1\DATA\LUCAS 2024-06-06 13-56-22\009-0301.D		
**Sample name:**	MePiper-Ph		6/6/2024 1:56:22 PM
**Location:**	Vial 9	**Injection date:**	
**Injection:**	1 of 1	**Injection volume:**	10.000
**Last changed:**	6/6/2024 1:31:59 PM		

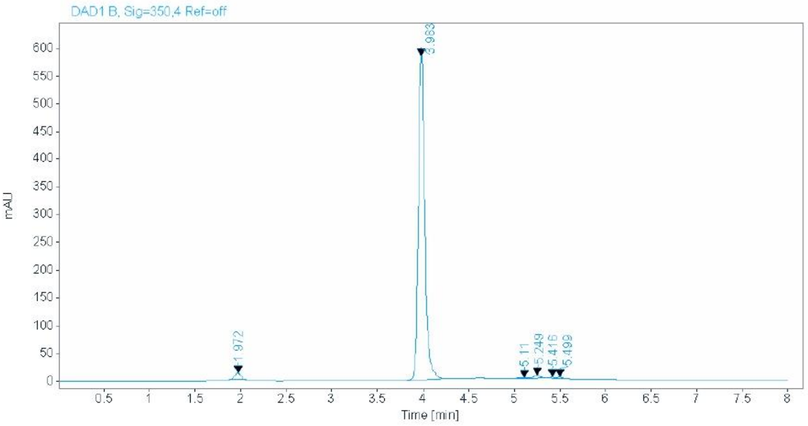

Signal: DAD1 B, Sig=350,4 Ref=off

RT [min]	Type	Width [min]	Area	Height	Area%	Name
1.972	MM	0.0750	49.5685	11.0143	1.7847	
3.983	BB	0.0723	2835.2324	587.3157	96.2636	
5.110	BV	0.1252	12.6786	1.6225	0.4309	
5.249	VB	0.0753	28.4025	5.5882	0.9653	
5.416	BV	0.0570	7.0874	1.9242	0.2409	
5.499	VB	0.0623	9.2554	2.3321	0.3146	

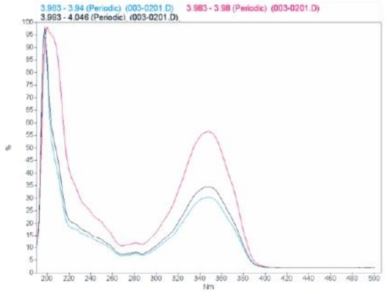

Figure S94. HPLC data for compound **1g.**

HPLC Report

LaDMol·QM

Agilent Technologies

**Data file:**	C:\CHEM32\1\DATA\LUCAS 2024-06-06 14-03-35\011-0101.D		
**Sample name:**	Piper-Ph		6/6/2024 2:03:45 PM
**Location:**	Vial 11	**Injection date:**	
**Injection:**	1 of 1	**Injection volume:**	10.000
**Last changed:**	6/6/2024 1:31:59 PM		

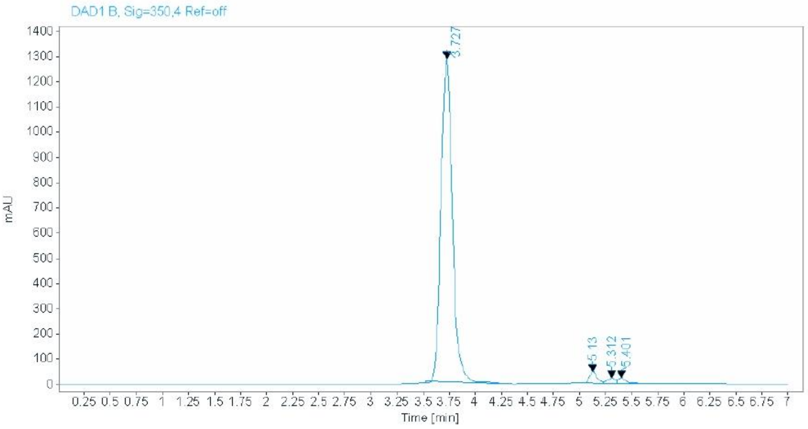

Signal: DAD1 B, Sig=350,4 Ref=off

RT [min]	Type	Width [min]	Area	Height	Area%	Name
3.727	MM	0.1278	9836.2979	1282.6835	95.2880	
5.130	BV	0.0798	226.0117	44.0980	2.2987	
5.312	VV	0.0770	101.4082	19.4048	1.1865	
5.401	VB	0.0817	115.8350	20.5728	1.2268	

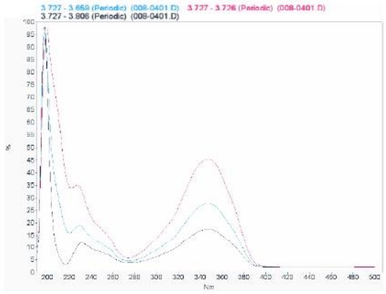

Figure S95. HPLC data for compound **1h.**

HPLC Report

LaDMol·QM

Agilent Technologies

**Data file:**	C:\CHEM32\1\DATA\LUCAS 2024-06-05 13-38-21\002-0101.D		
**Sample name:**	Bz		
**Location:**	Vial 2	**Injection date:**	6/5/2024 1:39:47 PM
**Injection:**	1 of 1	**Injection volume:**	10.000
**Last changed:**	6/5/2024 1:25:07 PM		

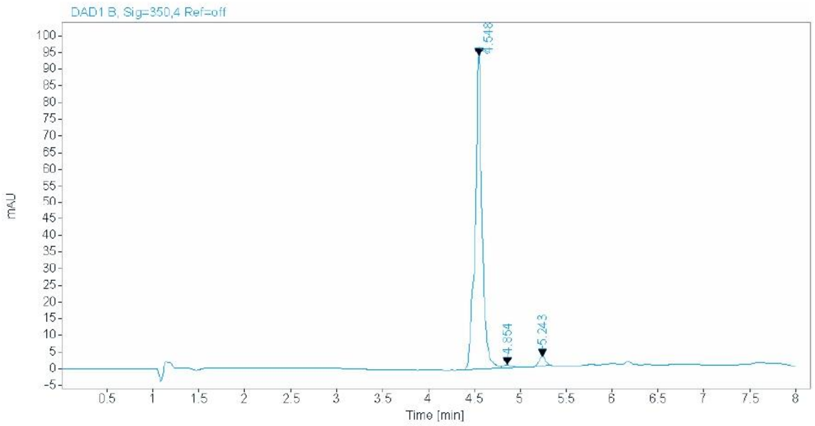

Signal: DAD1 B, Sig=350,4 Ref=off

RT [min]	Type	Width [min]	Area	Height	Area%	Name
4.548	BV	0.0731	496.8281	94.8134	96.0975	
4.854	VB	0.0990	7.3285	1.0061	1.4175	
5.243	MM	0.0794	12.8477	2.6979	2.4850	

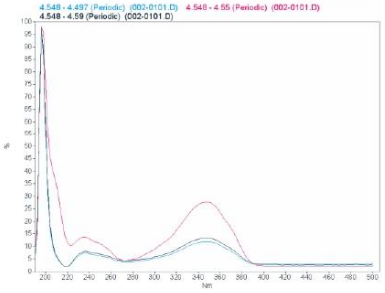

Figure S96. HPLC data for compound **1i.**

HPLC Report

LaDMol·QM

Agilent Technologies

Data file:	C:\CHEM32\1\DATA\LUCAS 2024-06-05 14-55-43\016-0401.D		
Sample name:	o-OCH3		6/5/2024 3:25:43 PM
Location:	Vial 16	Injection date:	
Injection:	1 of 1	Injection volume:	10.000
Last changed:	6/5/2024 2:31:59 PM		

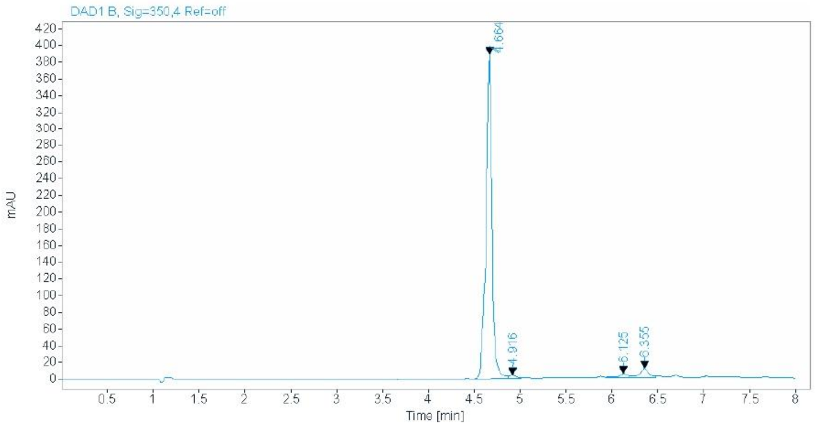

Signal: DAD1 B, Sig=350,4 Ref=off

RT [min]	Type	Width [min]	Area	Height	Area%	Name
4.664	BV	0.0664	1747.1519	389.1972	96.1953	
4.916	VV	0.0753	22.3328	4.2523	0.6273	
6.125	VV	0.0906	29.4656	4.4851	1.0094	
6.355	VV	0.0917	68.4972	10.5541	2.1680	

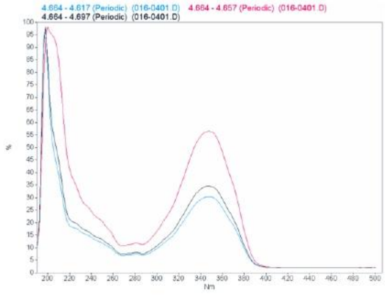

Figure S97. HPLC data for compound **1j.**

HPLC Report

LaDMol·QM

Agilent Technologies

**Data file:**	C:\CHEM32\1\DATA\LUCAS 2024-06-05 13-38-21\004-0301.D		
**Sample name:**	m-OCH3	**Injection date:**	6/5/2024 1:58:46 PM
**Location:**	Vial 4		
**Injection:**	1 of 1	**Injection volume:**	10.000
**Last changed:**	6/5/2024 1:25:07 PM		

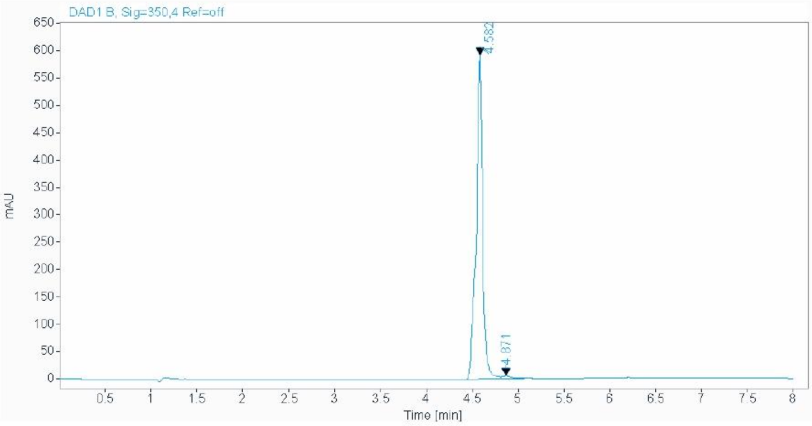

Signal: DAD1 B, Sig=350,4 Ref=off

RT [min]	Type	Width [min]	Area	Height	Area%	Name
4.582	BV	0.0708	2883.1311	592.2029	98.4794	
4.871	VV	0.1003	44.5184	5.8791	1.5206	

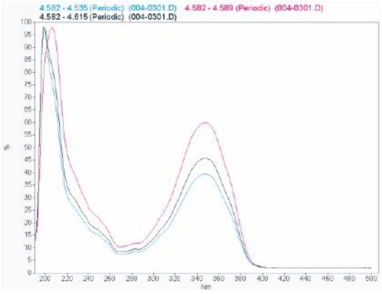

Figure S98. HPLC data for compound **1k.**

HPLC Report

LaDMol·QM

Agilent Technologies

Data file: C:\CHEM32\1\DATA\LUCAS 2024-06-05 13-38-21\003-0201.D

Sample name: p-OCH3

Location: Vial 3

Injection date: 6/5/2024 1:49:14 PM

Injection: 1 of 1

Injection volume: 10.000

Last changed: 6/5/2024 1:25:07 PM

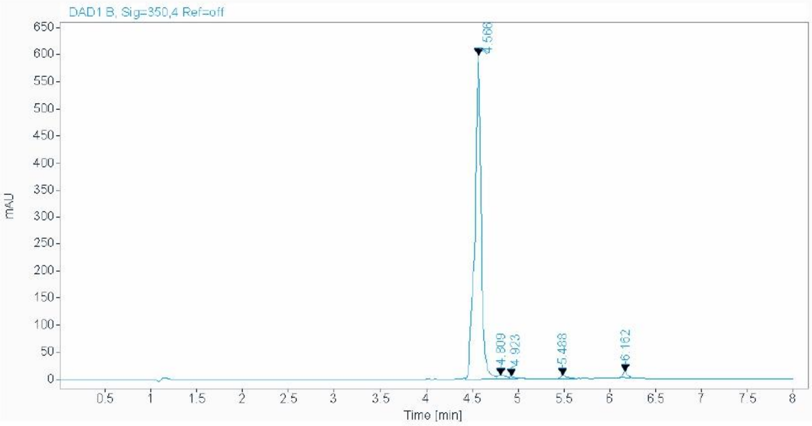

Signal: DAD1 B, Sig=350,4 Ref=off

RT [min]	Type	Width [min]	Area	Height	Area%	Name
4.566	VV	0.0679	2871.7249	599.7174	96.1194	
4.809	VV	0.0869	39.8575	6.2047	1.3341	
4.923	MF	0.0717	16.1325	3.7485	0.5400	
5.488	BB	0.0636	22.6119	5.3197	0.7568	
6.162	MM	0.0580	37.3386	10.7248	1.2498	

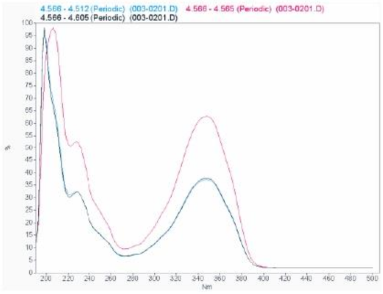

Figure S99. HPLC data for compound **1l.**

HPLC Report

LaDMol·QM

Agilent Technologies

**Data file:**	C:\CHEM32\1\DATA\LUCAS 2024-06-09 13-02-46\001-0101.D		
**Sample name:**	4-F		6/9/2024 1:02:53 PM
**Location:**	Vial 1	**Injection date:**	
**Injection:**	1 of 1	**Injection volume:**	10.000
**Last changed:**	6/9/2024 01:02:46 PM		

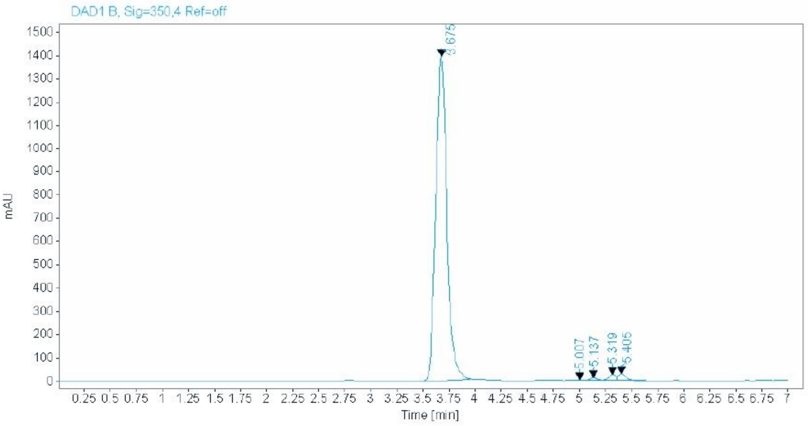

Signal: DAD1 B, Sig=350,4 Ref=off

RT [min]	Type	Width [min]	Area	Height	Area%	Name
3.675	MM	0.1241	10398.3750	1397.0388	97.3369	
5.007	BB	0.0755	5.3104	1.1578	0.0496	
5.137	BV	0.0726	49.7908	10.6474	0.3646	
5.319	VV	0.0691	115.4038	24.4268	0.9769	
5.405	VB	0.0769	147.0186	28.1776	1.2720	

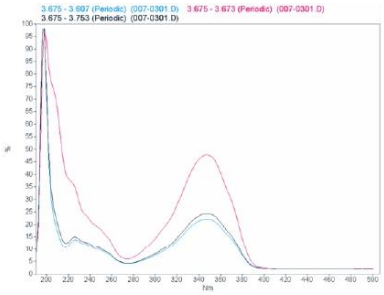

Figure S100. HPLC data for compound **1m.**

HPLC Report

LaDMol·QM

Agilent Technologies

Data file:	C:\CHEM32\1\DATA\LUCAS 2024-06-09 13-15-22\002-0101.D		
Sample name:	3-Cl		6/9/2024 1:15:17 PM
Location:	Vial 2	Injection date:	
Injection:	1 of 1	Injection volume:	10.000
Last changed:	6/9/2024 01:02:46 PM		

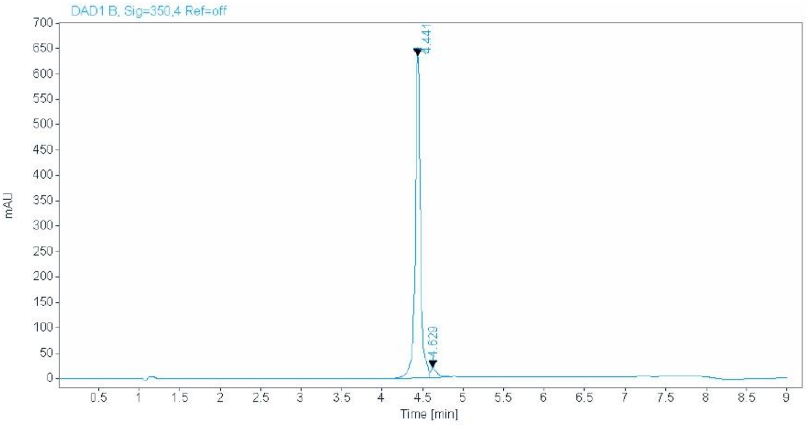

Signal: DAD1 B, Sig=350,4 Ref=off

RT [min]	Type	Width [min]	Area	Height	Area%	Name
4.441	BV	0.0654	2810.7625	637.7972	96.7017	
4.629	VV	0.0757	107.9420	19.7721	3.2983	

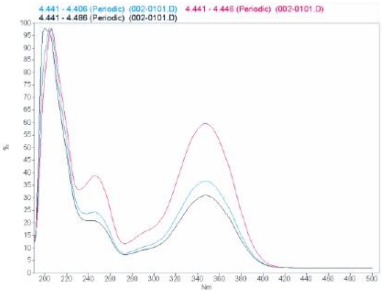

Figure S101. HPLC data for compound **1n.**

HPLC Report

LaDMol·QM

Agilent Technologies

**Data file:**	C:\CHEM32\1\DATA\LUCAS 2024-06-05 14-55-43\015-0301.D		
**Sample name:**	p-Cl	**Injection date:**	6/5/2024 3:16:11 PM
**Location:**	Vial 15		
**Injection:**	1 of 1	**Injection volume:**	10.000
**Last changed:**	6/5/2024 2:31:59 PM		

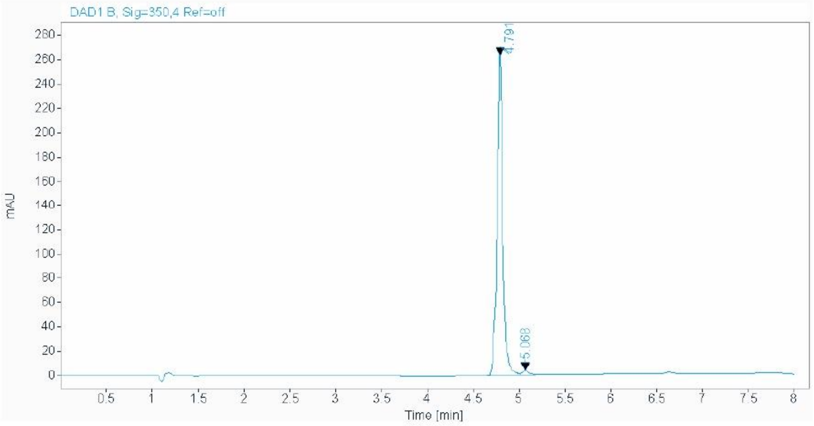

Signal: DAD1 B, Sig=350,4 Ref=off

RT [min]	Type	Width [min]	Area	Height	Area%	Name
4.791	BV	0.0618	1136.7079	266.4100	98.0970	
5.068	VB	0.0823	22.0516	3.7661	1.9030	

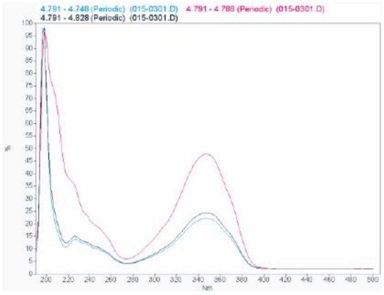

Figure S102. HPLC data for compound **1o.**

HPLC Report

Agilent Technologies

LaDMol·QM

**Data file:**	C:\CHEM32\1\DATA\LUCAS 2024-06-12 16-23-03\007-0701.D		
**Sample name:**	3-Br		6/12/2024 4:22:53 PM
**Location:**	Vial 7	**Injection date:**	
**Injection:**	1 of 1	**Injection volume:**	10.000
**Last changed:**	6/12/2024 3:31:59 PM		

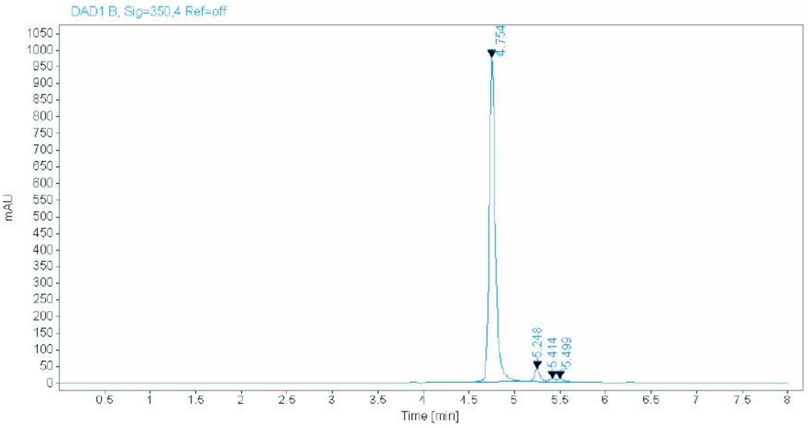

Signal: DAD1 B, Sig=350,4 Ref=off

RT [min]	Type	Width [min]	Area	Height	Area%	Name
4.754	BB	0.0728	4583.3462	975.0970	95.0903	
5.248	BV	0.0637	160.8639	37.7494	3.2308	
5.414	VV	0.0664	39.8527	8.8733	0.8252	
5.499	VB	0.0809	45.5776	7.9513	0.8537	

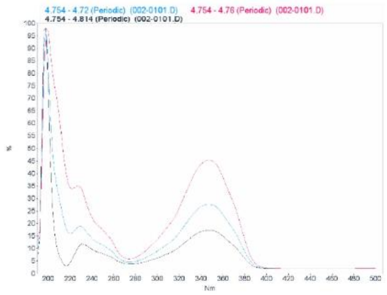

Figure S103. HPLC data for compound **1p.**

HPLC Report

LaDMol·QM

Agilent Technologies

**Data file:**	C:\CHEM32\1\DATA\LUCAS 2024-06-05 14-55-43\017-0501.D		
**Sample name:**	p-Br	**Injection date:**	6/5/2024 3:35:14 PM
**Location:**	Vial 17		
**Injection:**	1 of 1	**Injection volume:**	10.000
**Last changed:**	6/5/2024 2:31:59 PM		

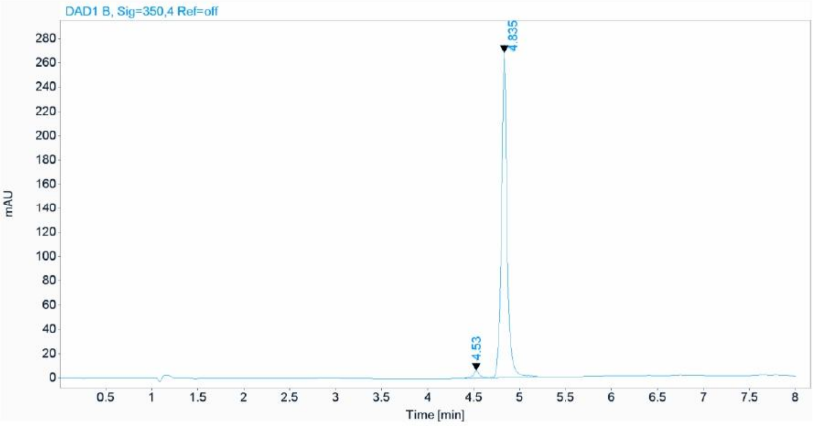

Signal: DAD1 B, Sig=350,4 Ref=off

RT [min]	Type	Width [min]	Area	Height	Area%	Name
4.530	BB	0.0701	29.4476	6.1241	2.5118	
4.835	BB	0.0638	1142.9392	267.8033	97.4882	

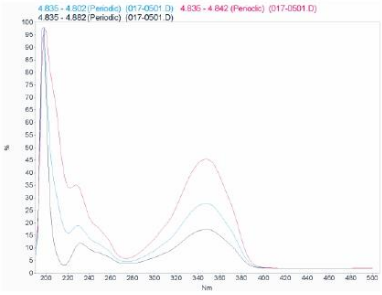

Figure S104. HPLC data for compound **1q.**

HPLC Report

LaDMol·QM

Agilent Technologies

**Data file:**	C:\CHEM32\1\DATA\LUCAS 2024-06-05 14-55-43\014-0201.D		
**Sample name:**	3C-Bz		6/5/2024 3:06:40 PM
**Location:**	Vial 14	**Injection date:**	
**Injection:**	1 of 1	**Injection volume:**	10.000
**Last changed:**	6/5/2024 2:31:59 PM		

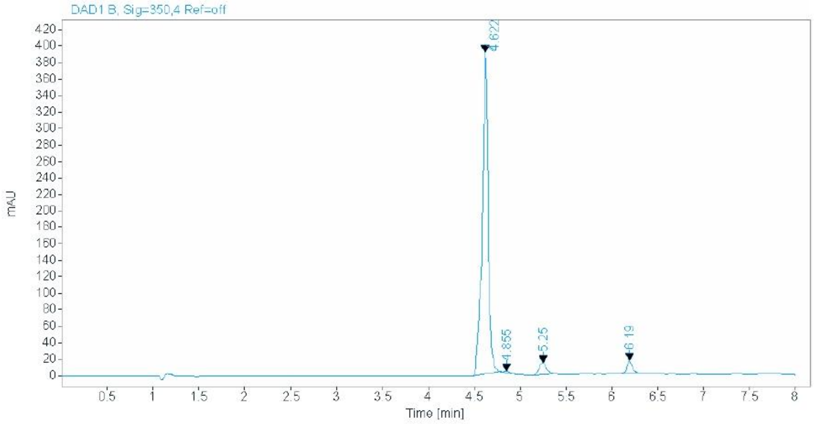

Signal: DAD1 B, Sig=350,4 Ref=off

RT [min]	Type	Width [min]	Area	Height	Area%	Name
4.622	MM	0.0743	1743.8656	391.4312	95.1738	
4.855	MM	0.0743	10.3754	2.3264	0.5496	
5.250	MM	0.0899	76.3946	14.1627	2.4465	
6.190	MM	0.0631	57.2006	15.1074	1.8300	

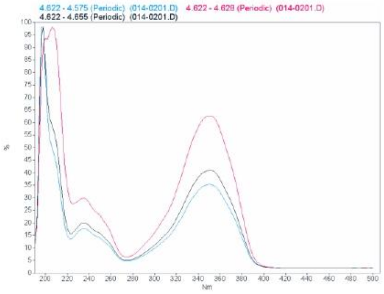

References

(1) De Souza, G. A.; Da Silva, S. J.; Del Cistia, C. D. N.; Pitasse-Santos, P.; Pires, L. D. O.; Passos, Y. M.; Cordeiro, Y.; Cardoso, C. M.; Castro, R. N.; Sant'Anna, C. M. R.; Kümmerle, A. E. Discovery of Novel Dual-Active 3-(4-(Dimethylamino)Phenyl)-7-Aminoalcoxy-Coumarin as Potent and Selective Acetylcholinesterase Inhibitor and Antioxidant. *J. Enzyme Inhib. Med. Chem.* **2019**, *34* (1), 631–637. https://doi.org/10.1080/14756366.2019.1571270.

(2) Ellman, G. L.; Courtney, K. D.; Andres, V.; Featherstone, R. M. A New and Rapid Colorimetric Determination of Acetylcholinesterase Activity. *Biochem. Pharmacol.* **1961**, *7* (2), 88–95. https://doi.org/10.1016/0006-2952(61)90145-9.

(3) Bautista-Aguilera, Ó. M.; Hagenow, S.; Palomino-Antolin, A.; Farré-Alins, V.; Ismaili, L.; Joffrin, P.; Jimeno, M. L.; Soukup, O.; Janočková, J.; Kalinowsky, L.; Proschak, E.; Iriepa, I.; Moraleda, I.; Schwed, J. S.; Romero Martínez, A.; López-Muñoz, F.; Chioua, M.; Egea, J.; Ramsay, R. R.; Marco-Contelles, J.; Stark, H. Multitarget-Directed Ligands Combining Cholinesterase and Monoamine Oxidase Inhibition with Histamine H_3 R Antagonism for Neurodegenerative Diseases. *Angew. Chem. Int. Ed.* **2017**, *56* (41), 12765–12769. https://doi.org/10.1002/anie.201706072.

(4) Bourne, Y.; Grassi, J.; Bougis, P. E.; Marchot, P. Conformational Flexibility of the Acetylcholinesterase Tetramer Suggested by X-Ray Crystallography. *J. Biol. Chem.* **1999**, *274* (43), 30370–30376. https://doi.org/10.1074/jbc.274.43.30370.

(5) Cheung, J.; Rudolph, M. J.; Burshteyn, F.; Cassidy, M. S.; Gary, E. N.; Love, J.; Franklin, M. C.; Height, J. J. Structures of Human Acetylcholinesterase in Complex with Pharmacologically Important Ligands. *J. Med. Chem.* **2012**, *55* (22), 10282–10286. https://doi.org/10.1021/jm300871x.

(6) Nachon, F.; Carletti, E.; Ronco, C.; Trovaslet, M.; Nicolet, Y.; Jean, L.; Renard, P.-Y. Crystal Structures of Human Cholinesterases in Complex with Huprine W and Tacrine: Elements of Specificity for Anti-Alzheimer's Drugs Targeting Acetyl- and Butyryl-Cholinesterase. *Biochem. J.* **2013**, *453* (3), 393–399. https://doi.org/10.1042/BJ20130013.
